# The Mechanical Properties and Microstructural Evolution Mechanism of Carbonation-Cured Loess with Varying MgO Content

**DOI:** 10.3390/ma19102107

**Published:** 2026-05-17

**Authors:** Kaiyuan Yang, Longqi Liu, Zhenhao Fan, Xinting Lu, Changqing Jia, Xingcan Mu, Bin Liu, Jianbin Zheng

**Affiliations:** 1 Transportation Institute, Inner Mongolia University, Hohhot 010020, China; yang15225536396@yeah.net (K.Y.); fanzhenhao1999@yeah.net (Z.F.); 2 Key Laboratory of Green Resource Utilization of Civil Engineering Waste, Universities of Inner Mongolia Autonomous Region, Hohhot 010020, China; 3 Inner Mongolia Engineering Research Center of Testing and Strengthening for Bridges, Inner Mongolia University, Hohhot 010020, China; 4 State Grid (Jinhua) Integrated Energy Service Company, No. 98 Jiefang East Road, Jinhua 321000, China; 5 General Office, Baotou Open University, Baotou 014010, China

**Keywords:** collapsible loess, MgO content, microstructural evolution, unconfined compressive strength

## Abstract

In alignment with global carbon neutrality goals, this study investigates the regulatory role of magnesium oxide (MgO) content on the macro–micro properties of carbonation-cured collapsible loess from the Hohhot region. While MgO carbonation is established for soil stabilization, the quantitative influence of MgO dosage on the specific phase evolution pathways and mechanical enhancement within the unique macro-porous fabric of aeolian loess remains poorly understood. Addressing this, we systematically examined loess specimens amended with varying MgO contents (10% to 30%) over carbonation periods up to 24 h. Unconfined compressive strength (UCS) tests, X-ray diffraction (XRD), and scanning electron microscopy (SEM) were employed to correlate mechanical performance with mineralogical and microstructural evolution. Results indicate that MgO content acts as a primary regulator of the carbonation process. Higher MgO dosages substantially increased CO_2_ uptake, resulting in a significant relative mass gain—up to more than a two-fold difference between the highest and lowest content samples—and culminated in a compressive strength of 10.48 MPa for the 30% MgO specimen. Microstructural analysis revealed a distinct temporal evolution interpreted to be governed by MgO-mediated supersaturation levels. Initially, Mg(OH)_2_ agglomerates provided early strength, which was subsequently enhanced by the formation of a three-dimensional framework of nesquehonite, followed by the development of an interlocking skeletal network of hydromagnesite crystals. These carbonate phases enhanced loess strength via a combination of pore infilling, particle cementation, and the construction of a reinforcing micro-skeleton. This work elucidates the link between MgO content and the microstructural evolution of carbonated loess, providing new insights for the synergistic integration of soil stabilization and carbon sequestration in loess regions. The findings offer a valuable reference for engineering applications in collapsible soil environments under the context of sustainable development.

## 1. Introduction

As a special engineering geological material, collapsible loess is prone to cause severe engineering problems such as differential foundation settlement and pavement cracking due to its inherent hydro-collapsibility and metastable macro-porous fabric [1]. These challenges are particularly acute in the Hohhot region, where the loess layer is notably thick and subject to intense freeze–thaw cycles and concentrated summer rainfall, necessitating effective and durable soil stabilization strategies.

The MgO carbonation technique presents a viable low-carbon alternative to conventional cement-based stabilization for such problematic soils. The efficacy of this technique is fundamentally governed by the MgO content, which exerts a decisive regulatory influence on the multi-stage reaction process. The MgO content not only modulates the rate of its hydration to form brucite (Mg(OH)_2_) [2] but also critically influences the subsequent metathesis reaction between Mg(OH)_2_ and carbon dioxide. While a high MgO content accelerates the initial hydration rate, it may simultaneously impair overall reaction efficiency due to particle agglomeration and the spatial hindrance imposed by the layered structure of precipitated carbonation products on CO_2_ diffusion [3]. Consequently, the impact of MgO content on the stabilization effect is inherently multidimensional, requiring a comprehensive understanding of the kinetic characteristics at each reaction stage. In-depth investigation into the influence of varying MgO dosages on the mechanical properties and microstructural evolution of collapsible loess is therefore of significant scientific importance and engineering application value [4].

Magnesium oxide carbonation stabilization technology, as a low-carbon and sustainable soil improvement method, has been systematically studied in the treatment of various types of soils. Research has primarily focused on the stabilization mechanisms of red clay [5] and contaminated soils [6], with gradual expansion into applications in subgrade filling engineering.

In the remediation of heavy metal-contaminated soils, Li Wentao et al. [2] proposed a method based on reactive MgO carbonation technology for treating manganese (Mn)- and cadmium (Cd)-contaminated soils. This technique utilizes the carbonation reaction between MgO and CO_2_ to achieve CO_2_ sequestration while facilitating the transformation of Cd into stable CdCO_3_. It effectively reduces the leaching concentrations of Cd and Mn to meet environmental standards and imparts favorable mechanical properties to the stabilized matrix. Zhang Yunhui et al. [7] compared the effectiveness of CaO and reactive MgO-activated ground granulated blast furnace slag (GGBS) in stabilizing zinc (Zn)-contaminated clay slurry. Their findings demonstrated that the MgO-GGBS system significantly enhances the unconfined compressive strength of the stabilized body and effectively immobilizes Zn, outperforming ordinary Portland cement (OPC). In contrast, the CaO-GGBS system resulted in inferior strength and stabilization efficacy due to the formation of calcium zinc hydroxide (CaZn_2_(OH)_6_·2H_2_O), which inhibits slag hydration. Microstructural analysis indicated that the predominance of calcium silicate hydrate (C-S-H) in the hydration products of the MgO-GGBS system is the primary factor contributing to its superior mechanical and stabilization performance.

In the reinforcement of soft clay and waste soils, Ji Henggang et al. [8] developed supersulfated cement (SSC) using MgO and building gypsum (BG) as composite activators for stabilizing waste soft clay (SC). At a mass ratio of m(GGBS):m(MgO):m(BG) = 6:2:2, the stabilized soil exhibited a 36.5–49.3% increase in 91-day unconfined compressive strength and a 39.1% strength retention rate after wet-dry cycles. The hydration products, predominantly C-S-H gel and ettringite (AFt), contributed to a dense and stable microstructure. Kong Xianghui et al. [9] employed reactive MgO, desulfurization gypsum (DG), and steel slag (SS) for synergistic stabilization of dredged sediments. They identified an optimal mix proportion (MgO 7.62%, DG 11.31%, SS 5.80%). This system improves soil structure through the encapsulation, cementation, and filling effects of hydration products, significantly enhancing mechanical properties and effectively immobilizing heavy metals.

For expansive soils, Wolfgang Jan Zucha et al. [10] investigated the stabilizing effect of an MgO based cementitious binder (MB) on smectite containing soils. Their research revealed that MB promotes the transformation of smectite into magnesium hydroxy interlayered smectite (Mg-HIS) within hours, significantly inhibiting its swelling-shrinkage characteristics. The mechanism involves the insertion of Mg^2+^ into the interlayer domains, yielding superior performance compared to traditional lime flocculation. Li Zhongyao et al. [11] utilized a composite of polyvinyl alcohol (PVA) and reactive MgO for sandy soil improvement. Their study showed that the composite improvement outperformed the use of PVA alone, with strength increasing over time. However, excessively high MgO content may lead to the formation of microcracks.

In the development of low-carbon building materials, Hu Chuanlin et al. [12] studied an MgO calcined clay cementitious system, significantly enhancing material performance via an initial CO_2_ stirring process. This process promotes the formation of highly reactive magnesium carbonate micro particles, which provide nucleation sites for hydration and react with calcined clay to form carbonate-type hydrotalcite, thereby improving the microstructure.

Furthermore, advancements in MgO-based cementitious materials have been driven by material innovations and process optimizations proposed by various researchers. Unluer and Al-Tabbaa [13] introduced the incorporation of hydrated magnesium carbonate to accelerate MgO carbonation, notably enhancing the CO_2_ sequestration capacity of blocks. Panesar et al. [14] investigated a MgO–slag–cement ternary system, achieving strength improvement and carbon emission reduction under accelerated curing conditions. Vandeperre et al. [15], through microstructural analysis, found that high MgO content leads to the formation of a composite structure filled and cemented by magnesium hydroxide and C-S-H gel. Liska et al. [16]. evaluated pressed block manufacturing parameters, demonstrating that MgO based blocks can achieve simultaneous mechanical enhancement and carbon emission reduction under appropriate processing conditions.

Domestic and international studies have confirmed that magnesium oxide carbonation stabilization technology offers advantages such as rapid strength development and low-carbon environmental friendliness in the modification of various soils and building materials. However, systematic research on its application in collapsible loess has received relatively limited attention, and the quantitative relationship between MgO content and the evolution of the loess-specific pore structure during carbonation has been seldom addressed. Building upon existing achievements and integrating the unique structural characteristics of loess, future research should investigate: (1) The relationship between carbon sequestration and mechanical enhancement under varying MgO contents; (2) The influence of microscopic phase transformations on macroscopic mechanical properties; and (3) The correlation between macroscopic crack formation and microstructural evolution.

Based on this foundation, this study focuses on collapsible loess collected from the transitional zone between the Inner Mongolia Plateau and the Loess Plateau. Controlled specimens with different MgO contents were prepared and subjected to carbonation stabilization treatment. Through systematic analysis of surface characteristics, basic physical properties, microstructural evolution, and unconfined compressive strength, this research comprehensively investigates the mechanisms and patterns of MgO content influencing collapsible loess. The aim is to provide a solid theoretical basis for engineering practice.

## 2. Experimental Materials and Program

### 2.1. Introduction to Sampling Site, Raw Soil and MgO Specimens

The test soil was collected along the route of Lime Kiln Village, National Highway G209, Helinger County, Hohhot City, Inner Mongolia. This area is located on the northeastern edge of the Loess Plateau, where Quaternary wind-deposited collapsible loess is widely distributed, exhibiting typical characteristics such as loose and porous structure, well-developed vertical joints, and susceptibility to disintegration upon contact with water. The loess layer in this region is notably thick, with low natural moisture content and a high collapsibility coefficient [17,18]. Such loess is prone to induce engineering issues like uneven foundation settlement and road surface cracking in road construction, significantly compromising engineering safety and durability.

**Figure 1:**
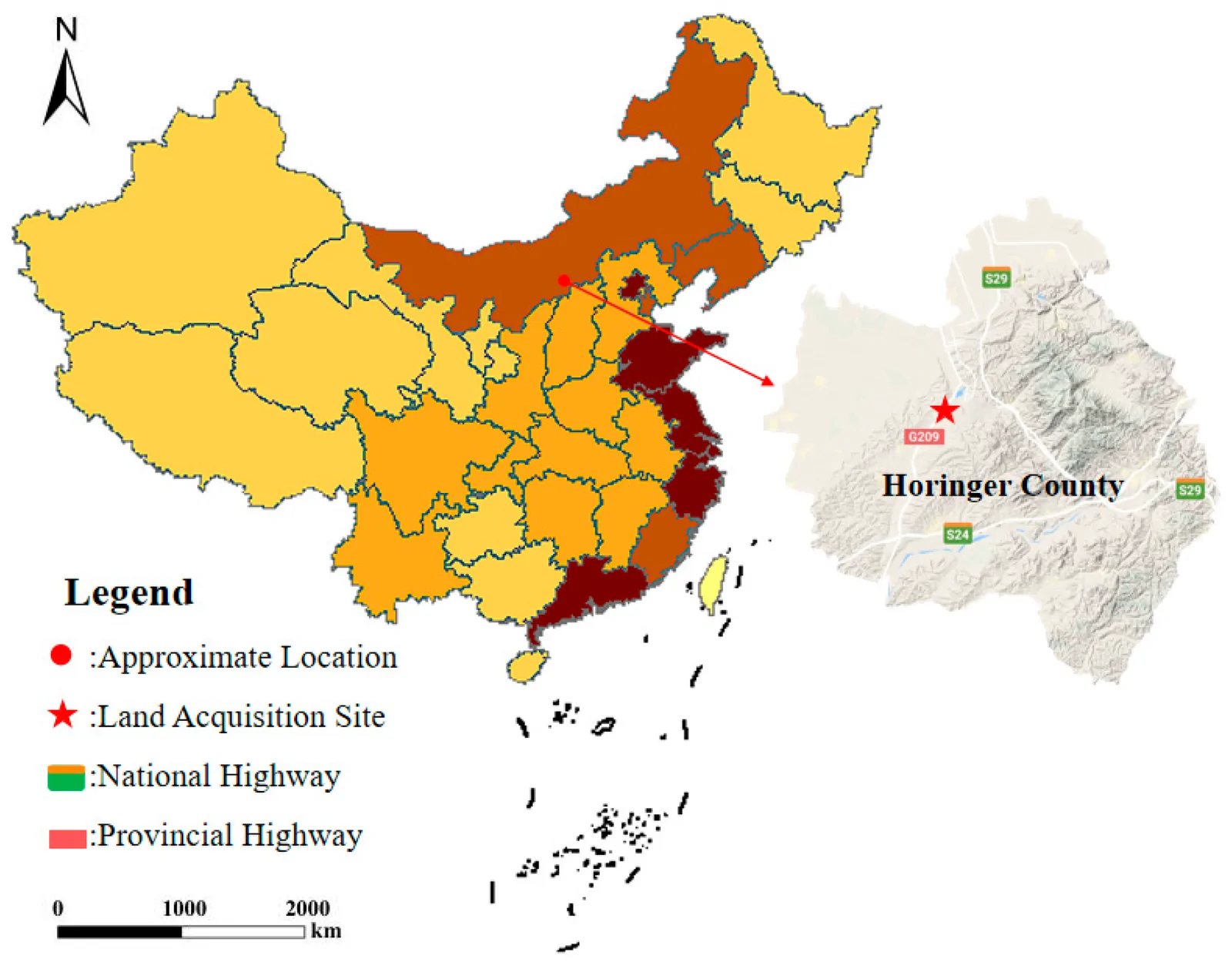
Soil sampling location.

The sampling site at Lime Kiln Village (Figure 1) is situated at an elevation of approximately 1200 m, within a mid-temperate continental monsoon climate zone. Winters are cold and prolonged (with extreme lows reaching −30 °C), while summers are short and hot. The annual temperature variation exceeds 40 °C, and intense freeze–thaw cycles exacerbate the physical weathering of the loess structure. The annual precipitation is about 398.2 mm, with over 70% concentrated between June and August. Short-duration heavy rainfall easily triggers loess collapse. Simultaneously, the region experiences prevalent northwesterly winds with an average annual wind speed of 4.5 m/s, leading to significant wind erosion and dust transport [19]. These processes result in loose surface soil texture and reduced shear strength. The coupling effect of the aforementioned climatic and hydrological conditions further aggravates the engineering geological problems of the loess, posing severe challenges to road subgrade stability [20]. Therefore, investigating improvement techniques for collapsible loess, particularly the regulatory mechanism of MgO carbonation stabilization on its mechanical properties, is of great significance for enhancing the long-term stability of road engineering in this region.

The collapsibility coefficient is a critical geotechnical indicator used to evaluate the deformation potential of soil upon wetting. Laboratory oedometer tests are conducted on undisturbed specimens with a diameter of 79.8 mm and a height of 20 mm. An initial seating pressure of 1 kPa was applied, and the gauge reading was zeroed. The specimen was then subjected to staged vertical loads of 50, 100, 150, and 200 kPa, and the stabilized height under natural moisture content (hp) was recorded at the 200 kPa level. Subsequently, the specimen was inundated with water and allowed to saturate under the sustained 200 kPa pressure until stabilization, at which point the post-inundation height ( ${h_{p}}^{\prime }$ ) was measured. The collapsibility coefficient (*δ_s_*) [21] was calculated using Equation (1):

(1) $$\delta_{s}=\frac{h_{p}-{h_{p}}^{\prime }}{h_{0}}\times 100\%$$

In the formula: *h_p_* represents the stabilized height of the specimen (maintaining natural moisture content and structure) under a specific applied pressure after compression; ${h_{p}}^{\prime }$ denotes the stabilized height of the same specimen after immersion in water and saturation, following additional compression under the same pressure; *h*_0_ is the original height of the specimen. To ensure reproducibility, three replicate specimens were prepared and tested under identical conditions.

The mean collapsibility coefficient obtained was 0.06475. According to the classification criteria for loess collapsibility (where 0.015–0.03 indicates slight, 0.03–0.07 indicates moderate, and >0.07 indicates severe collapsibility), the loess used in this study is classified as moderately collapsible.

### 2.2. Experimental Program

**Figure 2:**
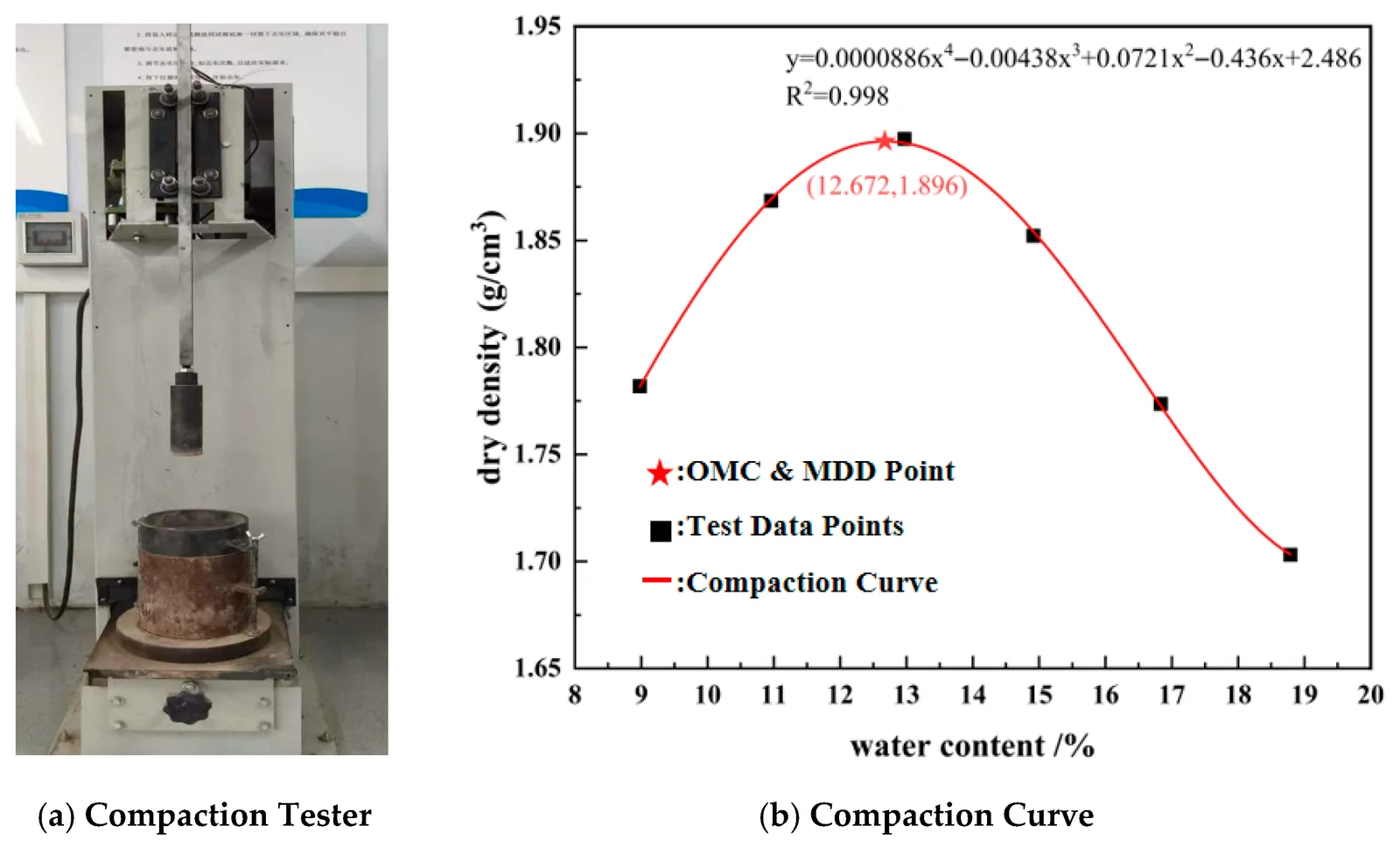
Photos of the compaction test and curves of optimal dry density and optimal moisture content.

To determine the compaction parameters of the collapsible loess, the Standard Proctor compaction test was conducted in strict accordance with the Chinese national standard (JTG 3430-2020) “Test Methods of Soils for Highway Engineering”. A light compaction apparatus (Figure 2a) from Tianjin Luda Construction Instrument Co., Ltd., Tianjin 300000, China, was employed in this study. The test utilized a 2.5 kg hammer dropped from a height of 30 cm, with the soil compacted in three layers and 27 blows applied per layer, corresponding to a compaction energy of approximately 600 kN·m/m^3^.

Six specimens with varying moisture contents were prepared. The wet density was measured for each specimen, and the corresponding dry density (*ρ_d_*) was calculated using the measured moisture content. The dry density values were plotted against the moisture content, and a second-order polynomial curve was fitted to the data points. From this fitted curve, the optimum moisture content was determined to be 12.662%, and the maximum dry density was determined to be 1.895 g/cm^3^ (Figure 2b). These values were subsequently used as target parameters for the preparation of all stabilized soil specimens in this study.

**Figure 3:**
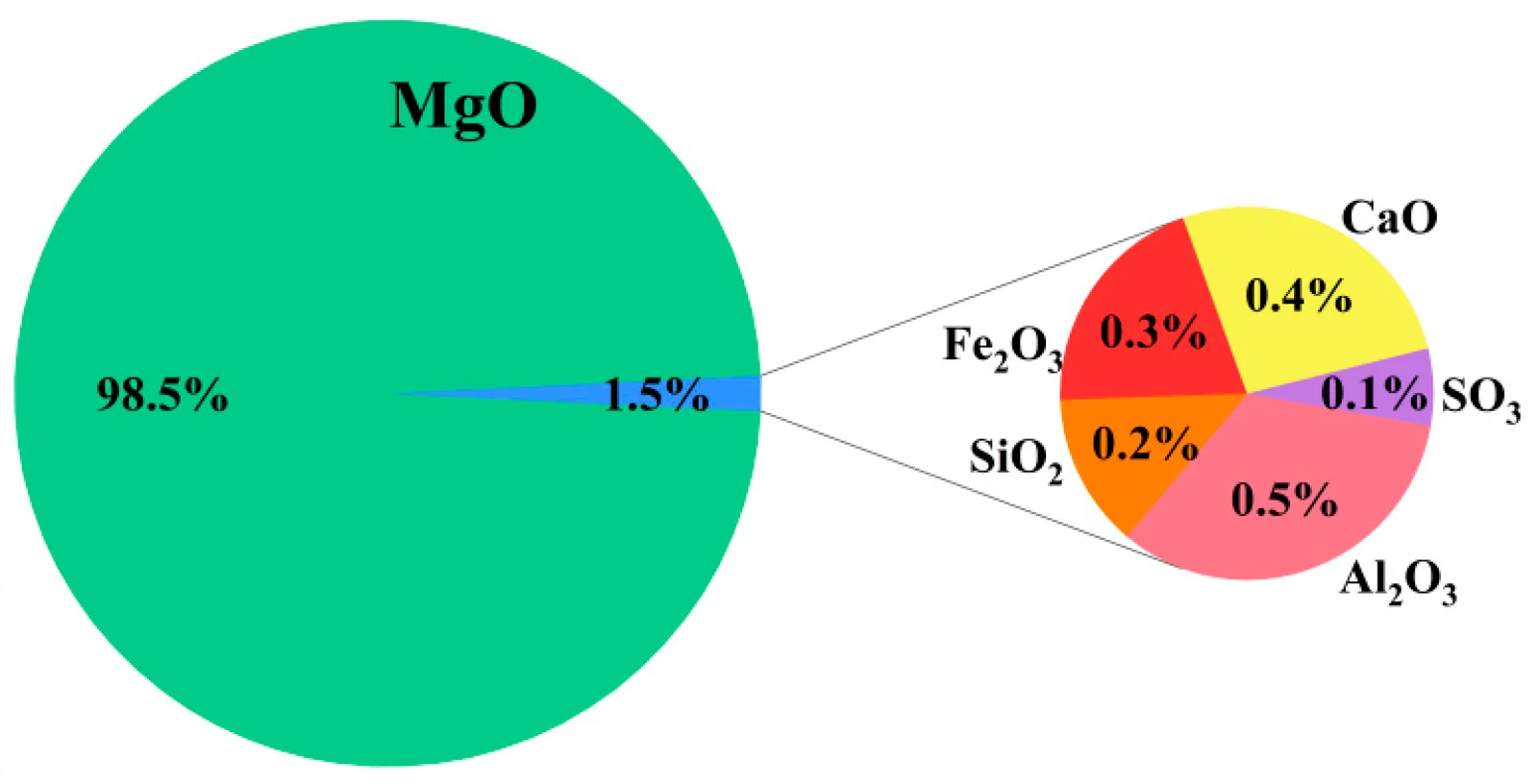
Mass Percentage of Chemical Components of MgO.

The basic physical indices of the loess are shown in Table 1 below:

The MgO powder used in the experiment was supplied by Tianjin Chemical Reagent Co., Ltd., (Tianjin 300000, China). Its chemical composition is provided in Figure 3:

#### Experimental Program

**Figure 4:**
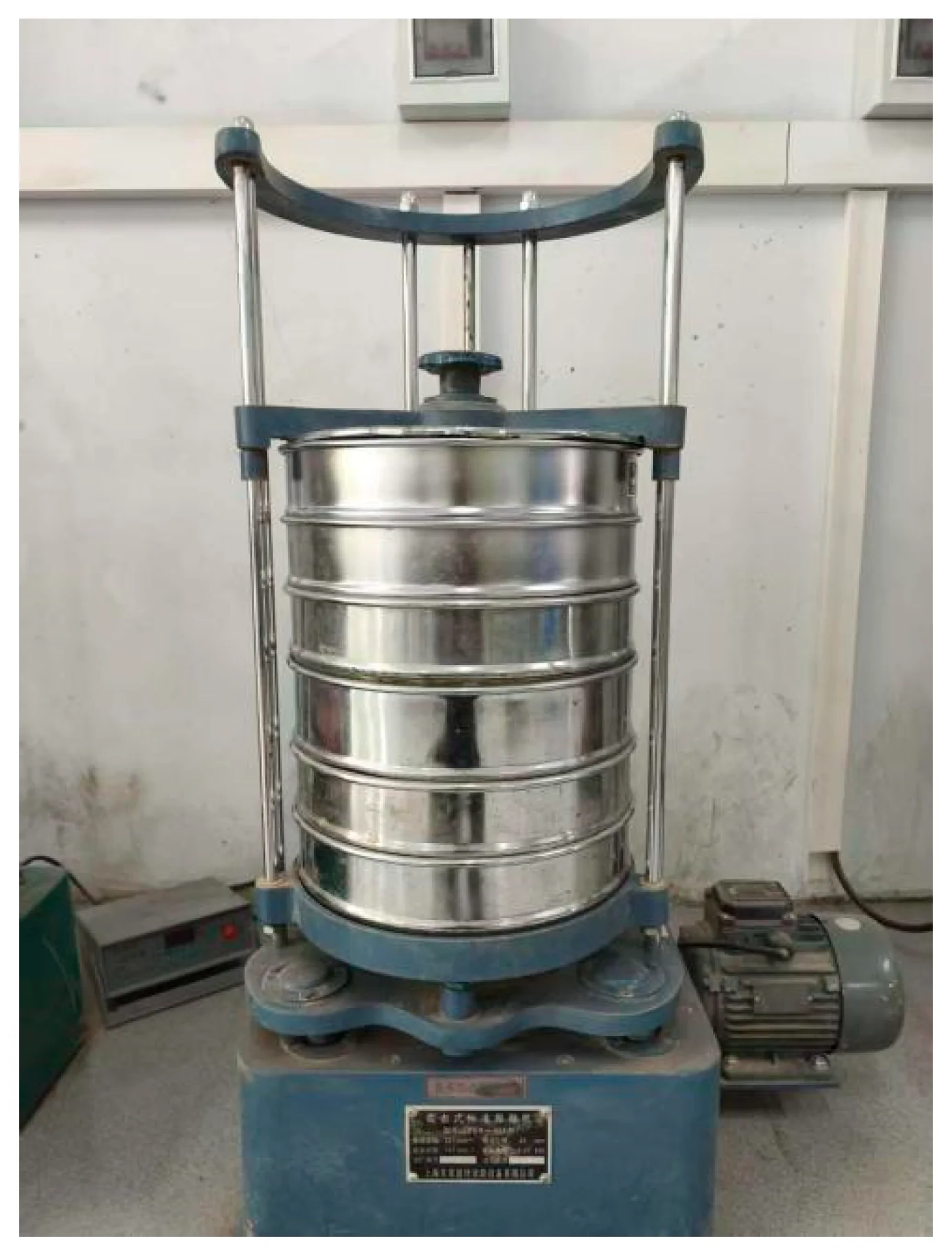
Sieve test apparatus.

**Figure 5:**
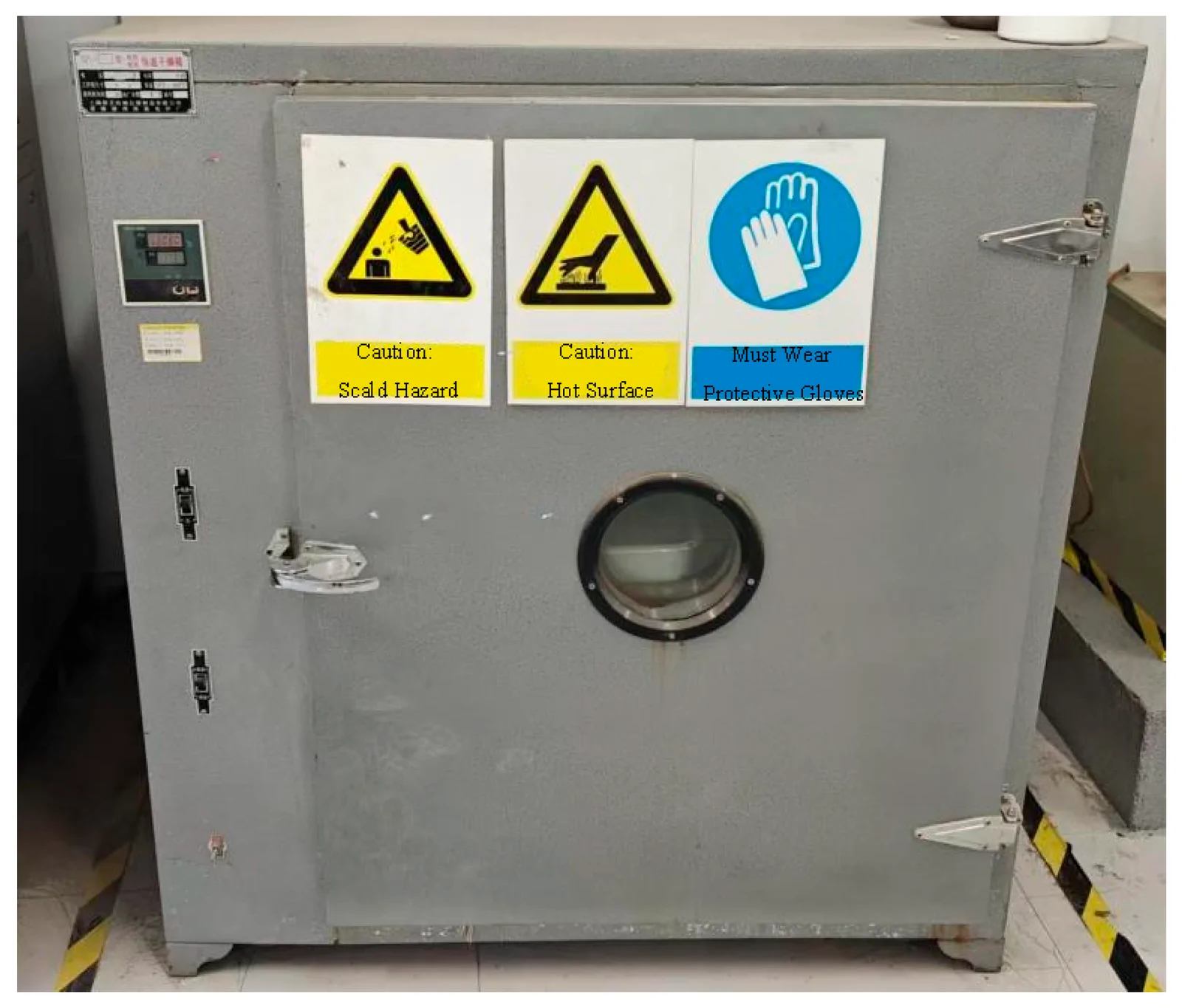
View of the thermostatic drying oven.

This study aims to investigate the effects of different MgO contents (10%, 15%, 20%, 25%, and 30% by dry mass of soil) on the properties of collapsible loess. Prior to specimen fabrication, the raw loess was mechanically crushed and passed through a 2 mm sieve (Shanghai Leiyun Experimental Instrument Manufacturing Co., Ltd. Shanghai 200000, China) to remove coarse fragments and ensure particle size uniformity (Figure 4). The sieved soil was then placed in an electric thermostatic oven (Shanghai Leiyun Experimental Instrument Manufacturing Co., Ltd., Shanghai 200000, China) and dried at 105 °C for a minimum of 24 h until a constant mass was achieved (Figure 5), thereby eliminating the influence of initial moisture on the subsequent hydration and carbonation reactions.

For each batch, the dried soil was weighed and combined with the designated mass of MgO powder. The dry constituents were manually mixed for approximately 5 min using a spatula and stainless-steel bowl until the mixture exhibited a uniform color with no visible agglomerations of MgO. Subsequently, the required mass of distilled water was gradually added in three equal increments. After each water addition, the mixture was stirred continuously. The total wet mixing duration was maintained at 8–10 min. The end-point of mixing was determined visually by the achievement of a homogeneous, cohesive paste devoid of any dry powder pockets or color striations.

**Figure 6:**
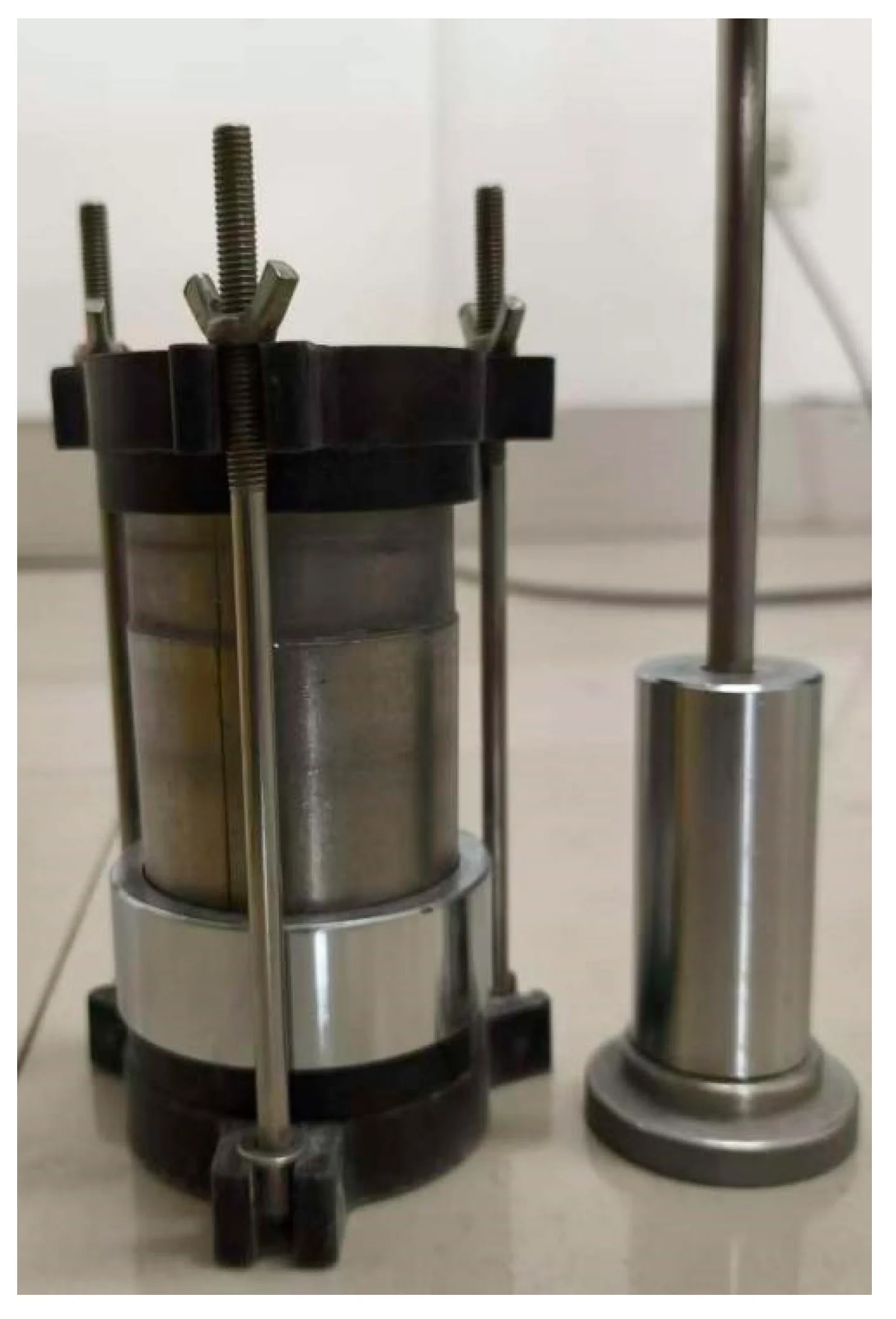
The 50 mm × 100 mm specimen mold.

**Figure 7:**
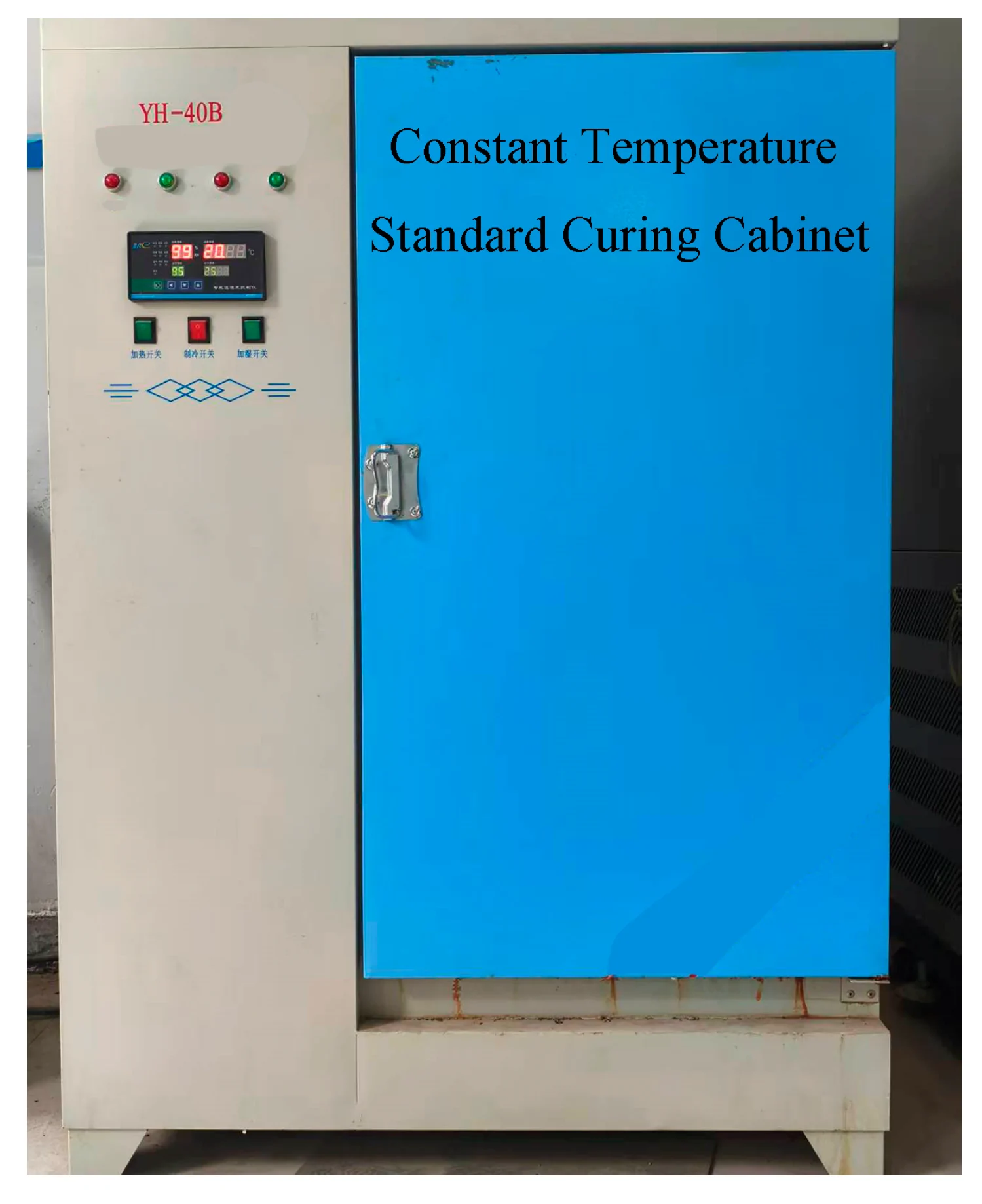
Constant temperature and humidity curing chamber.

The mixture was compacted in a 50 mm × 100 mm cylindrical mold (Nanjing Tuoce Instrument Equipment Co., Ltd., Nanjing 211100, China) using a multi-layer compaction method to achieve the target structural integrity (Figure 6) Immediately after compaction, the specimens were carefully demolded and sealed with plastic wrap to prevent moisture evaporation and uncontrolled carbonation. The sealed specimens were then placed in a constant temperature and humidity curing chamber (Tianjin Jianyi Test Instrument Factory, Tianjin 300000, China) and cured for 12 h under controlled conditions of 20 ± 2 °C and ≥95% relative humidity (Figure 7). This standardized curing regime was adopted to facilitate the complete and uniform hydration of MgO to form brucite (Mg(OH)_2_), which serves as the essential precursor for the subsequent carbonation reaction, while simultaneously minimizing microcracking induced by desiccation.

**Figure 8:**
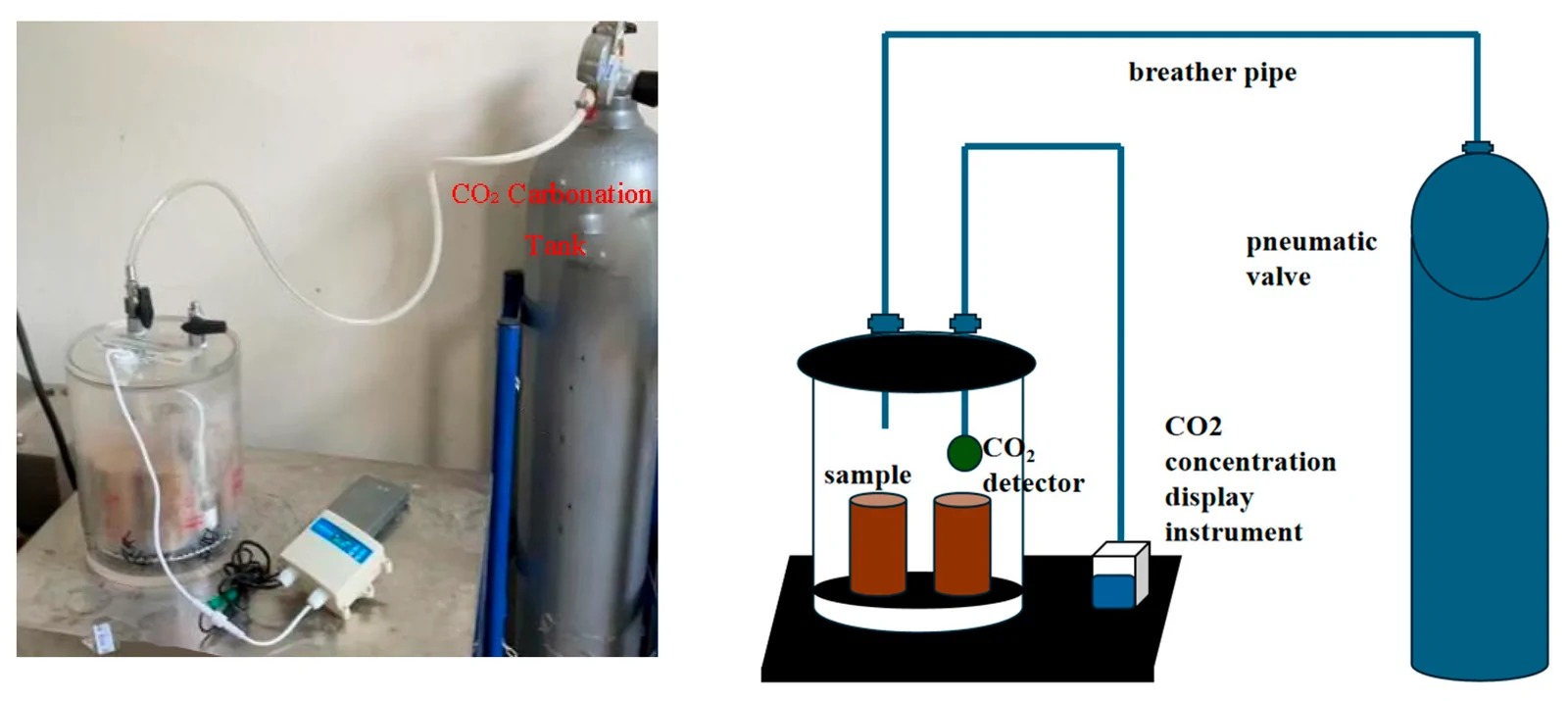
Physical intention and schematic diagram of carbonization device.

Following the 12 h moist curing period, the diameter, height, and mass of each specimen were precisely measured and recorded. The specimens were then placed inside a custom-built transparent acrylic carbonation chamber (Figure 8), which consisted of a modified vacuum desiccator. A circular metal mesh was positioned at the bottom of the chamber to elevate the specimens above any water condensate generated during the reaction. High-purity CO_2_ gas (≥99.9% purity, supplied by Tumed Left Banner Helin Gas Plant, Hohhot 010200, China) was introduced into the chamber from a 40 L cylinder via a flexible tube that extended to the bottom of the vessel, utilizing the principle of upward air displacement to purge ambient air. A CO_2_ sensor was placed in the mid-upper region of the chamber and connected to an external monitor to continuously track the gas concentration. The CO_2_ flow rate was manually regulated to ensure that the internal concentration was maintained at ≥50% throughout the test duration. The ambient laboratory temperature and relative humidity during carbonation were 18–28 °C and 50–60%, respectively.

Carbonation durations were set at discrete intervals of 0 h, 6 h, 12 h, 18 h, and 24 h. Upon completion of each designated carbonation period, specimens were removed from the chamber. Mass change measurement and volumetric expansion analysis were conducted immediately. The specimens were then subjected to a 7-day curing period, followed by unconfined compressive strength (UCS) testing (the unconfined compressive strength test adopts Model WAW-300B microcomputer-controlled universal testing machine, manufactured by Tianshui Hongshan Testing Machine Co., Ltd., Tianshui 741000, China).

Unconfined compressive strength tests were performed at a shear deformation rate of 1%/min [22], with compressive strength data recorded for each specimen [23]. Changes in mass and volume were calculated to analyze internal transformations during carbonation across different samples. CO_2_ concentration data were recorded, and weighted average values were computed to evaluate carbonation rates over distinct time intervals.

Upon completion of each carbonation stage, representative specimens with MgO contents of 10%, 20%, and 30% were selected for detailed mineralogical and microstructural analysis. Approximately 10 g of powdered material was extracted from the core region of each specimen to avoid surface effects. The powders were dried in an electric thermostat oven at 35 °C for 24 h to remove residual free water while preserving the integrity of the hydrated carbonate phases.

X-ray Diffraction (XRD): Phase identification was performed using a Rigaku SmartLab X-ray diffractometer (Rigaku Corporation, Tokyo 196-8666, Japan) operating with Cu Kα radiation ( λ = 1.5406 Å). The X-ray tube was operated at 40 kV and 40 mA. Diffraction patterns were collected over a 2θ range of 10° to 50° at a scanning rate of 2°/min with a step size of 0.02°. Qualitative phase analysis was conducted by comparing the acquired patterns with reference data from the International Centre for Diffraction Data database.

Scanning Electron Microscopy (SEM): Microstructural morphology was examined using a TESCAN MIRA field-emission scanning electron microscope (TESCAN ORSAY HOLDING, a.s., Kohoutovice 623 00, Czech). The dried powder samples were affixed to aluminum stubs with double-sided carbon adhesive tape and sputter-coated with gold to mitigate surface charging. Imaging was performed in secondary electron mode at an accelerating voltage of 15 kV.

## 3. Analysis and Discussion of Experimental Results

### 3.1. Analysis of Carbonation Reaction Kinetics

**Figure 9:**
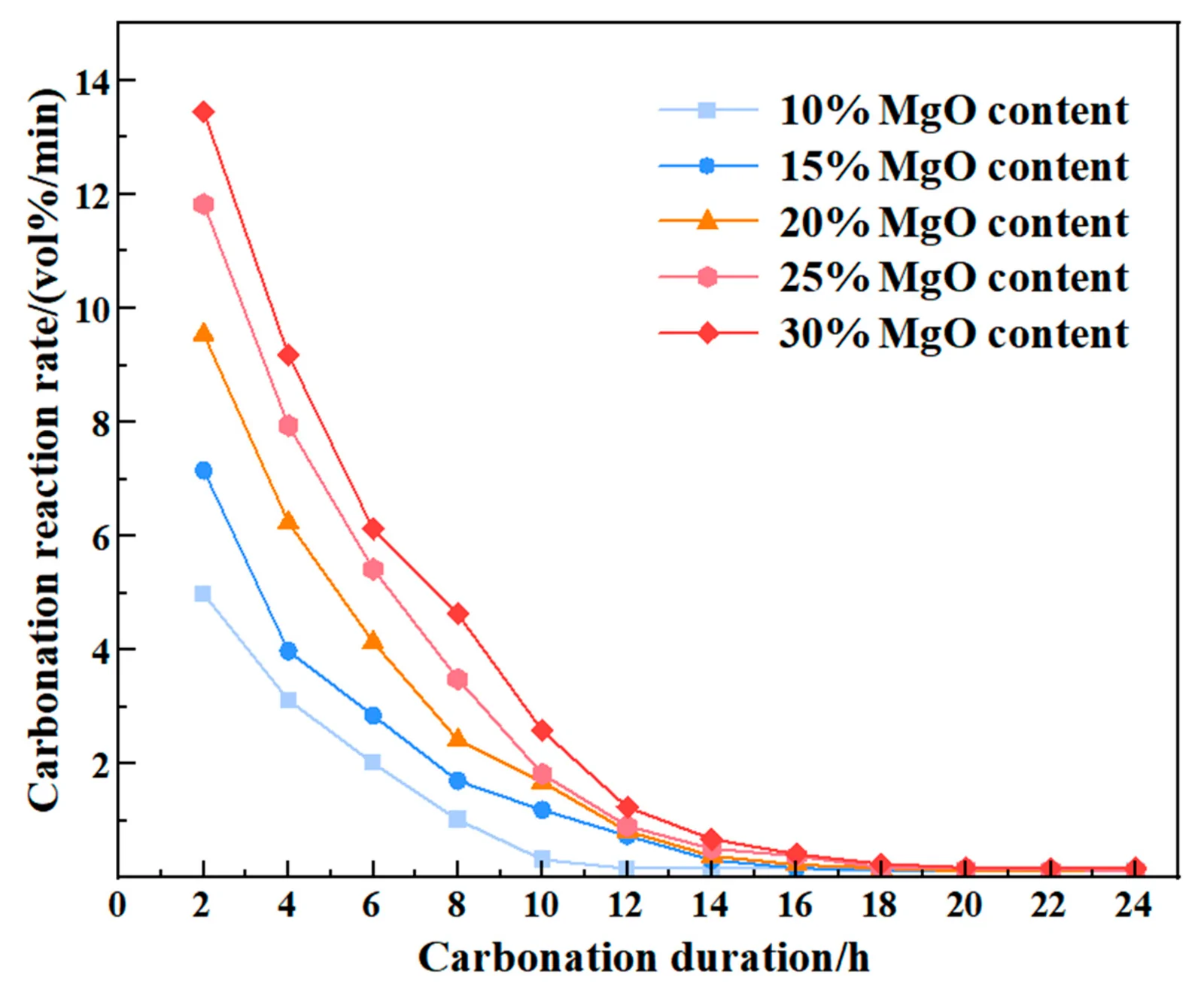
The Carbonation reaction rate of 10–30% MgO samples.

As a key variable in the carbonation reaction system, MgO content significantly influences the reaction rate and process stability [24]. Experimental data, as shown in Figure 9, indicate that during the 0–18 h reaction stage, the carbonation rate of all specimens exhibited a time-dependent decay trend, accompanied by a notable content-gradient effect. Specifically, under identical reaction durations, MgO content showed a positive correlation with the reaction rate. Specimens with 30% MgO content displayed the highest carbonation rate, confirming the accelerating effect of higher MgO dosages on the carbonation process.

Notably, beyond 18 h, the carbonation rate curve exhibited a precipitous attenuation and gradually stabilized, particularly in high-content specimens (20–30%). Microstructural observations revealed that this phenomenon can be attributed to two primary mechanisms: (1) In high-MgO-content specimens, the surface layer preferentially completed carbonation, generating substantial secondary minerals such as nesquehonite (MgCO_3_·3H_2_O), dypingite (Mg_5_(CO_3_)_4_(OH)_2_·5H_2_O), and hydromagnesite (Mg_5_(CO_3_)_4_(OH)_2_·4H_2_O). These crystalline precipitates progressively filled the interparticle and mesoscopic pores, thereby obstructing the diffusion channels for CO_2_ contents [25]; (2) As the reaction progressed, unreacted MgO within the specimen cores was gradually consumed, diminishing the chemical driving force and allowing the carbonation process to approach a state of near-equilibrium.

In summary, high-MgO-content specimens exhibited superior reaction kinetics during the initial carbonation stage (0–12 h) due to enriched active sites. However, with prolonged reaction time, the rate attenuation amplitude was significantly greater than in low-content systems. This phenomenon reveals a nonlinear coupling mechanism among MgO content, pore structure, and reaction kinetics, providing a theoretical basis for optimizing carbonation stabilization processes. A similar time-dependent constraint was noted by Li et al. [26] in the context of MgO-carbonated waste silt, where the unconfined compressive strength increased with MgO content and appropriate carbonation duration.

### 3.2. Volumetric Expansion of Soils with Different MgO Contents

**Figure 10:**
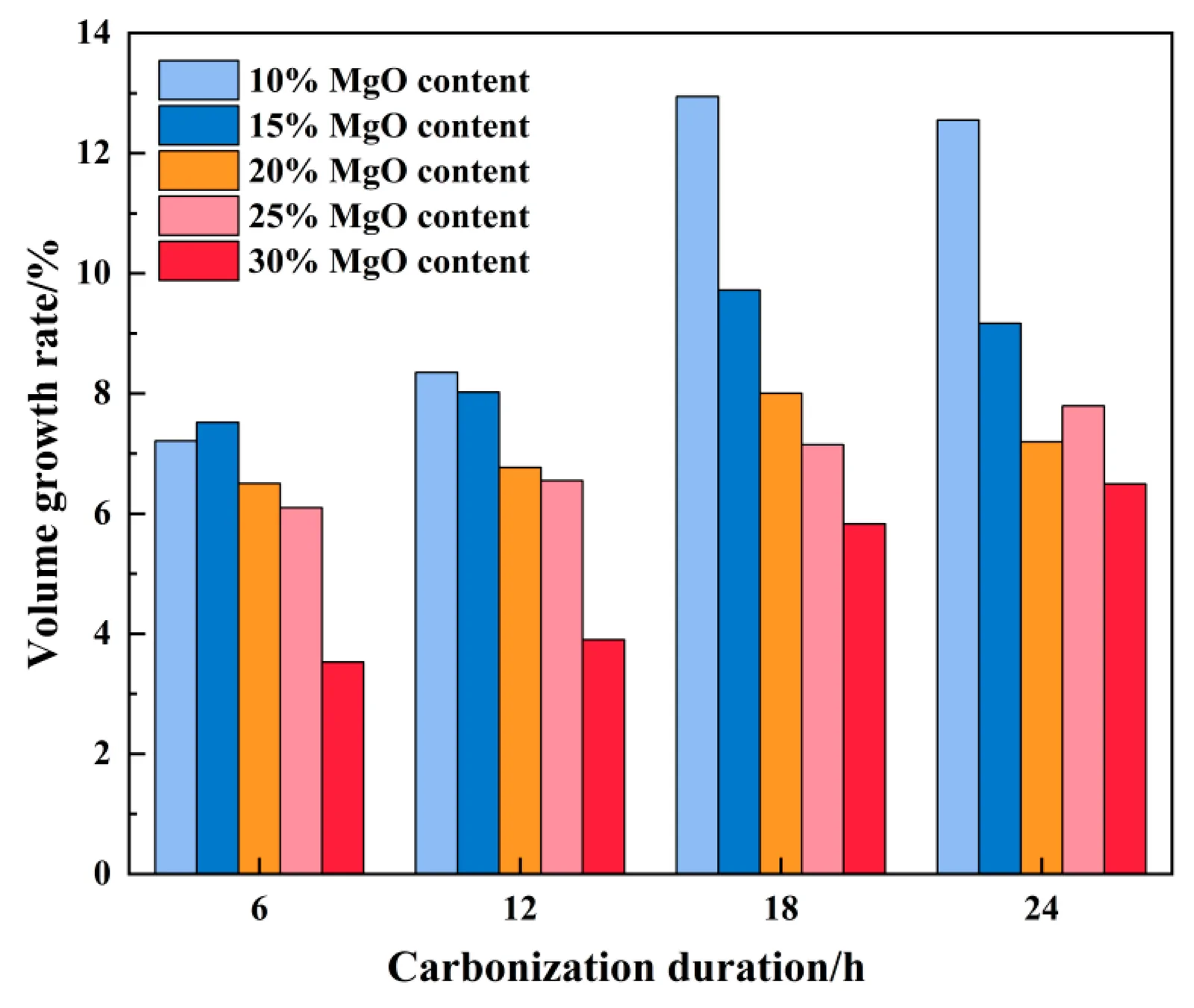
The volume growth rate of samples with different MgO contents.

Figure 10 illustrates the evolution of volumetric expansion rates for specimens with varying MgO dosages over the carbonation period. During the initial 6–18 h reaction interval, the volumetric expansion of all specimens tended to increase progressively with time. Notably, specimens with higher MgO content generally exhibited comparatively lower volumetric expansion rates during this phase. As the reaction approached 24 h, a divergence in behavior was observed: specimens with lower MgO contents (10–20%) showed a tendency toward stabilization or a slight decrease in expansion rate, whereas those with higher MgO contents (25–30%) continued to display a modest upward trend.

This phenomenon is primarily attributed to differences in MgO content. During the hydration stage, the Mg(OH)_2_ generated in high-content specimens filled the pores, increasing the volumetric baseline. Consequently, despite their faster carbonation reaction and greater product formation, the volumetric expansion rate was relatively lower. Additionally, the agglomeration of Mg(OH)_2_ partially restricted the full progression of the carbonation reaction.

As the reaction continued, the carbonation process in low-MgO-content specimens gradually approached saturation, leading to a decline in the volumetric expansion rate after reaching its peak. In contrast, high-content specimens sustained ongoing carbonation, thereby maintaining a higher volumetric expansion rate.

### 3.3. Analysis of Mass Increase in Soil Samples with Different MgO Contents

**Figure 11:**
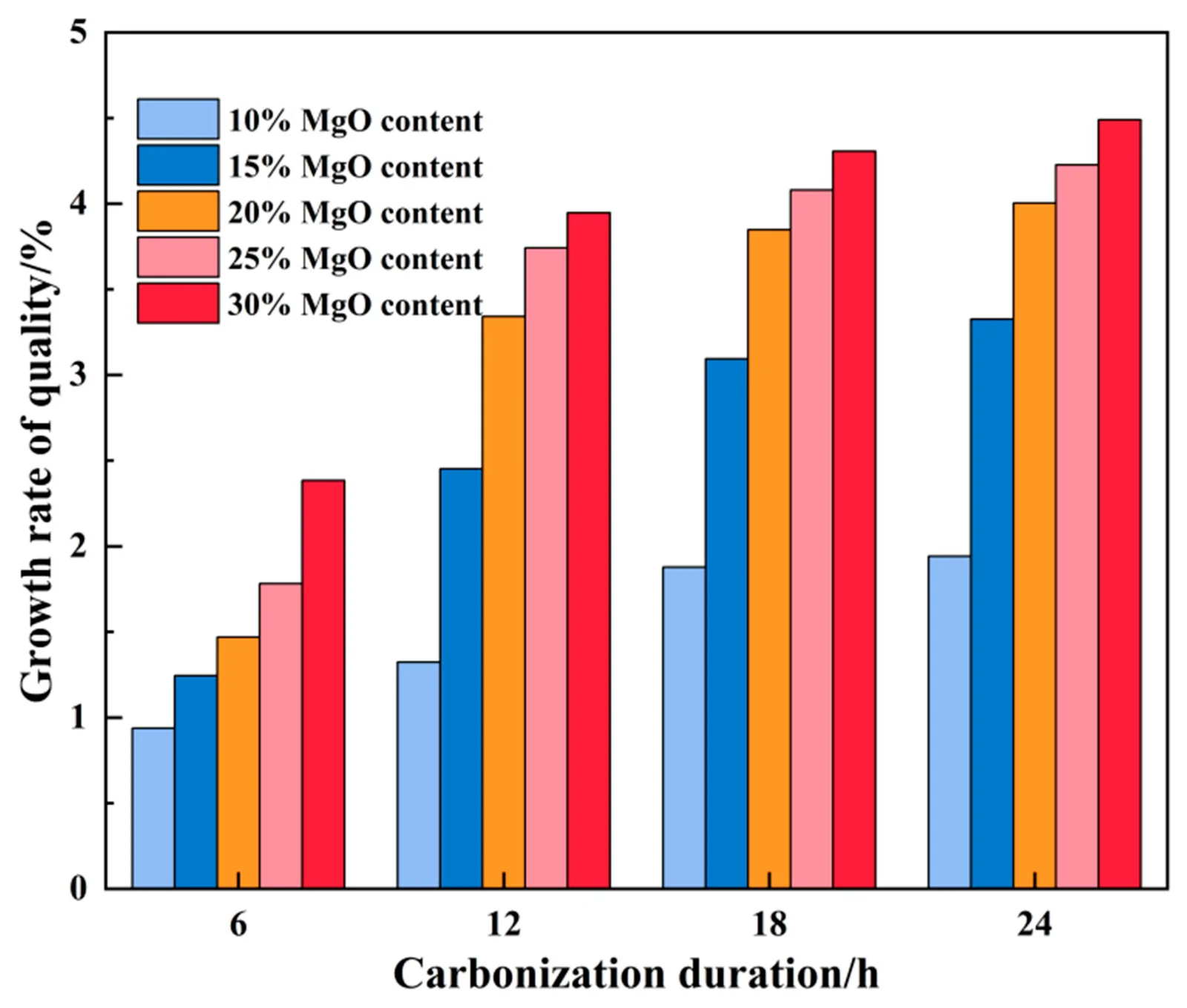
Mass growth rate of samples with different MgO contents.

As illustrated in Figure 11, the net mass growth rate of the specimens exhibited a clear increasing trend with higher MgO content. After 12 h of carbonation, the difference in mass growth rate was more pronounced among specimens with lower MgO dosages (10%, 15%, and 20%), while the differences among those with 20%, 25%, and 30% MgO appeared relatively modest, suggesting a gradual saturation in net mass accumulation at higher dosages.

It should be noted that the observed mass change represents the combined outcome of multiple concurrent processes, including MgO hydration, water redistribution, and CO_2_ incorporation. Therefore, the mass growth rate is presented as a macroscopic indicator of overall reaction progression rather than a direct measure of CO_2_ uptake.

In summary, within the low-to-medium MgO content range, net mass accumulation increased notably with MgO dosage. Beyond approximately 20% MgO, the incremental gain in mass tended to diminish, indicating a transition toward reaction-limited or diffusion-limited conditions.

### 3.4. Estimation of Carbonation Completion Time and Weighted Strength Parameter

**Figure 12:**
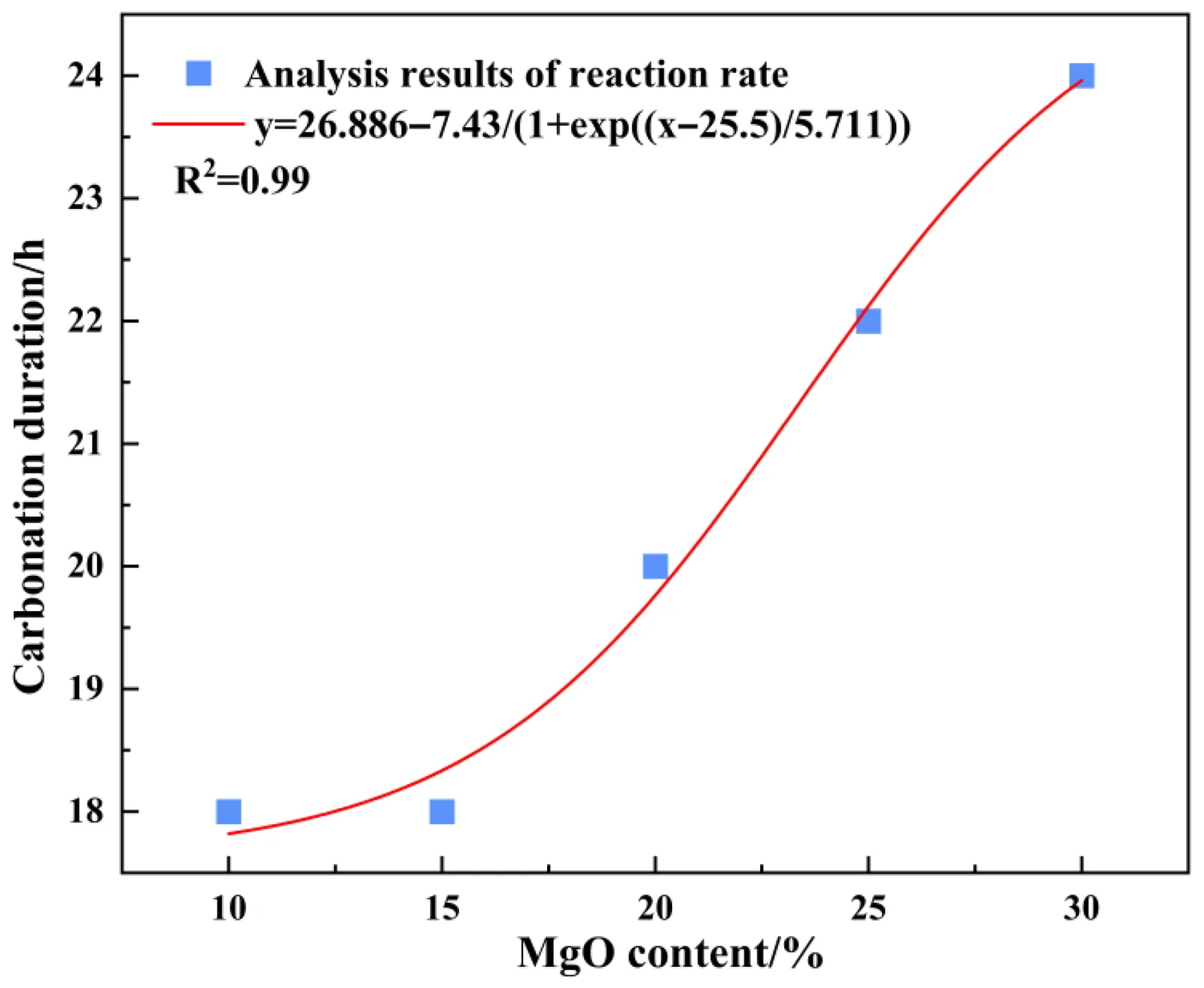
Plot of weighted average carbonation duration vs. MgO content.

For practical engineering purposes, it is useful to estimate the time required for the accelerated carbonation process to approach completion under the experimental conditions. Based on the reaction rate analysis presented in Section 3.1, the carbonation rate exhibited a consistent time-dependent decay across all MgO dosages. As a practical criterion, the carbonation reaction was considered to be nearing completion when the instantaneous carbonation rate dropped below 0.15 vol%/min. Applying this threshold to the rate curves shown in Figure 6, the estimated times to reach this state were approximately 18 h for the 10% and 15% MgO specimens, and 20 h, 22 h, and 24 h for the 20%, 25%, and 30% MgO specimens, respectively. These values are summarized in Figure 12.

The unconfined compressive strength (UCS) of the specimens was measured at discrete carbonation intervals (0, 6, 12, 18, and 24 h). The complete set of experimental UCS data is presented in Figure 9. As expected, the UCS values increased significantly with both increasing MgO content and extended carbonation duration, reflecting the progressive formation and accumulation of cementitious carbonate phases.

**Figure 13:**
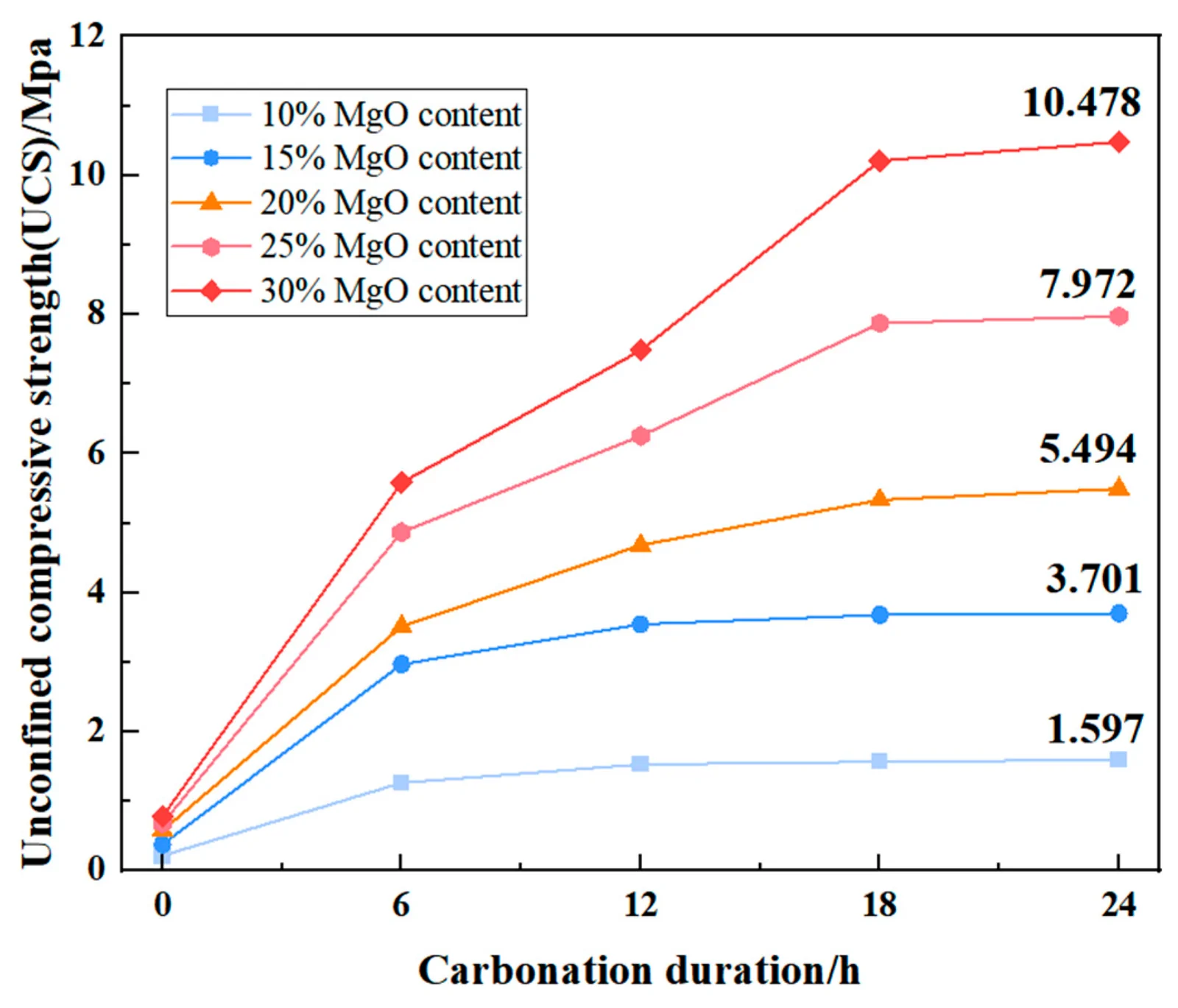
Unconfined compressive strength of MgO with different contents.

The unconfined compressive strength (UCS) of the specimens was evaluated at discrete carbonation intervals of 0, 6, 12, 18, and 24 h. The full set of experimental UCS data is presented in Figure 13. As anticipated, UCS values exhibited a substantial increase with both higher MgO content and prolonged carbonation duration, a trend attributable to the progressive formation and accumulation of cementitious carbonate phases. This observation is consistent with the findings of Song et al. [27], who demonstrated that activated MgO carbonation effectively enhances the strength characteristics of clayey soils through the precipitation of hydrated magnesium carbonates.

Based on a weighted analysis of unconfined compressive strength with respect to carbonation duration, the following equation was established (2).

(2) qu′=qu+1(qu−2qu)1(Ta−T1)6

Herein, ${q_{u}}^{\prime }$ represents the weighted unconfined compressive strength; *q_u_*^1^ denotes the unconfined compressive strength at the preceding time point; *q_u_*^2^ signifies the unconfined compressive strength at the subsequent time point; *T_a_* stands for the weighted carbonation duration; and *T*_1_ indicates the carbonation duration at the preceding time point.

This weighted strength parameter provides a practical index for comparing the strength development efficiency across different MgO dosages under the specific accelerated carbonation regime employed in this study.

While the weighted UCS parameter offers a useful empirical metric, it does not fully capture the stress–strain behavior or failure mechanisms of the stabilized soil. To further interpret the experimental UCS trends and to provide a predictive framework for potential engineering applications, a series of finite element simulations were undertaken using the commercial software Abaqus 2022. The following section (Section 3.5) describes the development and validation of this numerical model.

### 3.5. Fitting & Predicting UCS of Specimens with Different MgO Contents (Abaqus-Based)

**Figure 14:**
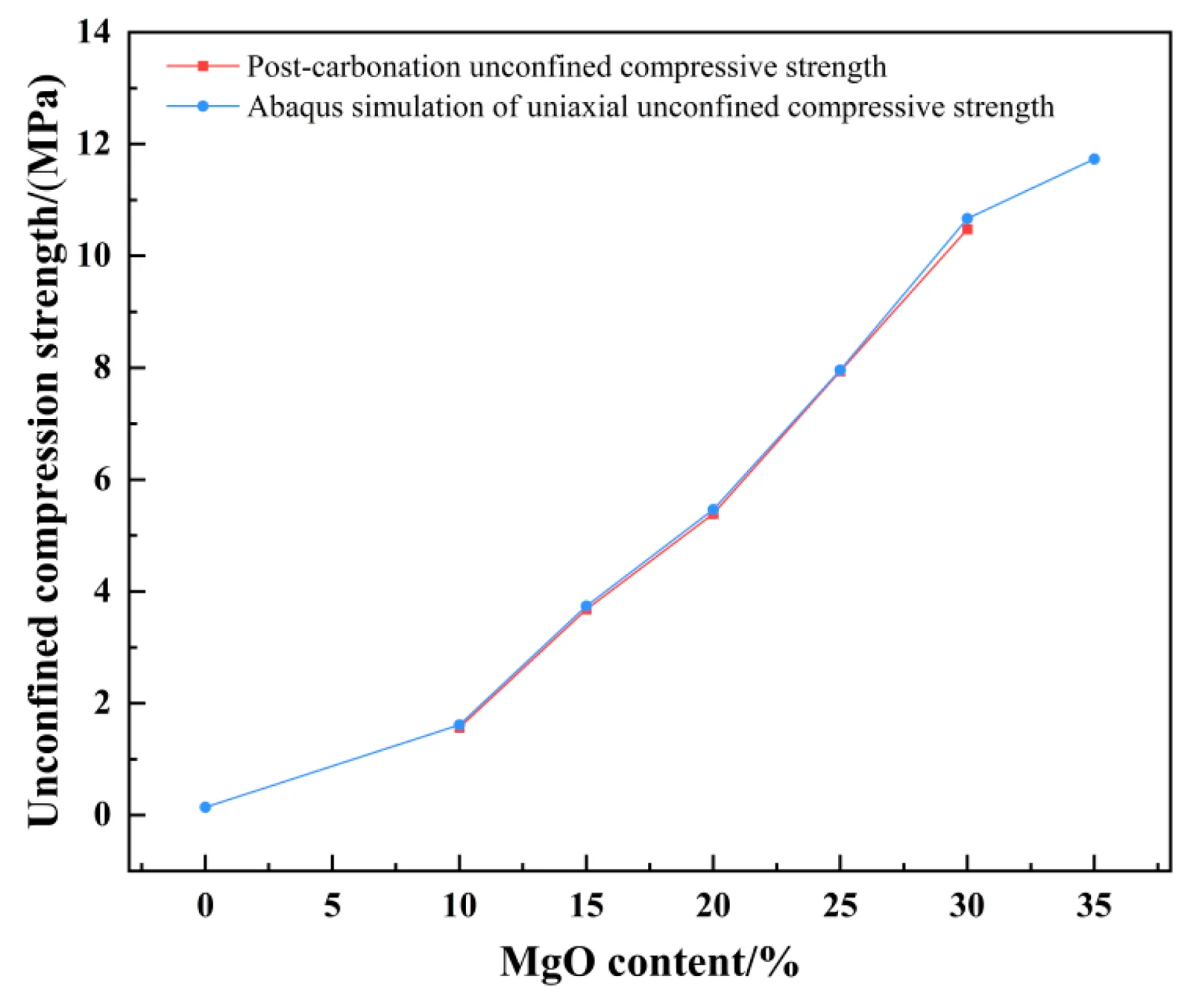
Based on realistic conditions, the simulation results for the unconfined compressive strength were derived using Abaqus.

To extend the investigation of practical conditions, this study employed the Abaqus finite element software to conduct numerical simulations based on prior experimental results obtained from specimens with MgO contents ranging from 10% to 30%. By precisely matching the model dimensions to the experimental specimens and integrating constitutive parameters from public database models for loess and stabilized soil [28], the unconfined compressive strength responses under similar stress conditions were obtained through multiple validations and fitting iterations. Leveraging this validated numerical framework, the performance of a specimen with 35% MgO content was further predicted. The corresponding simulation results are illustrated in Figure 14.

**Figure 15:**
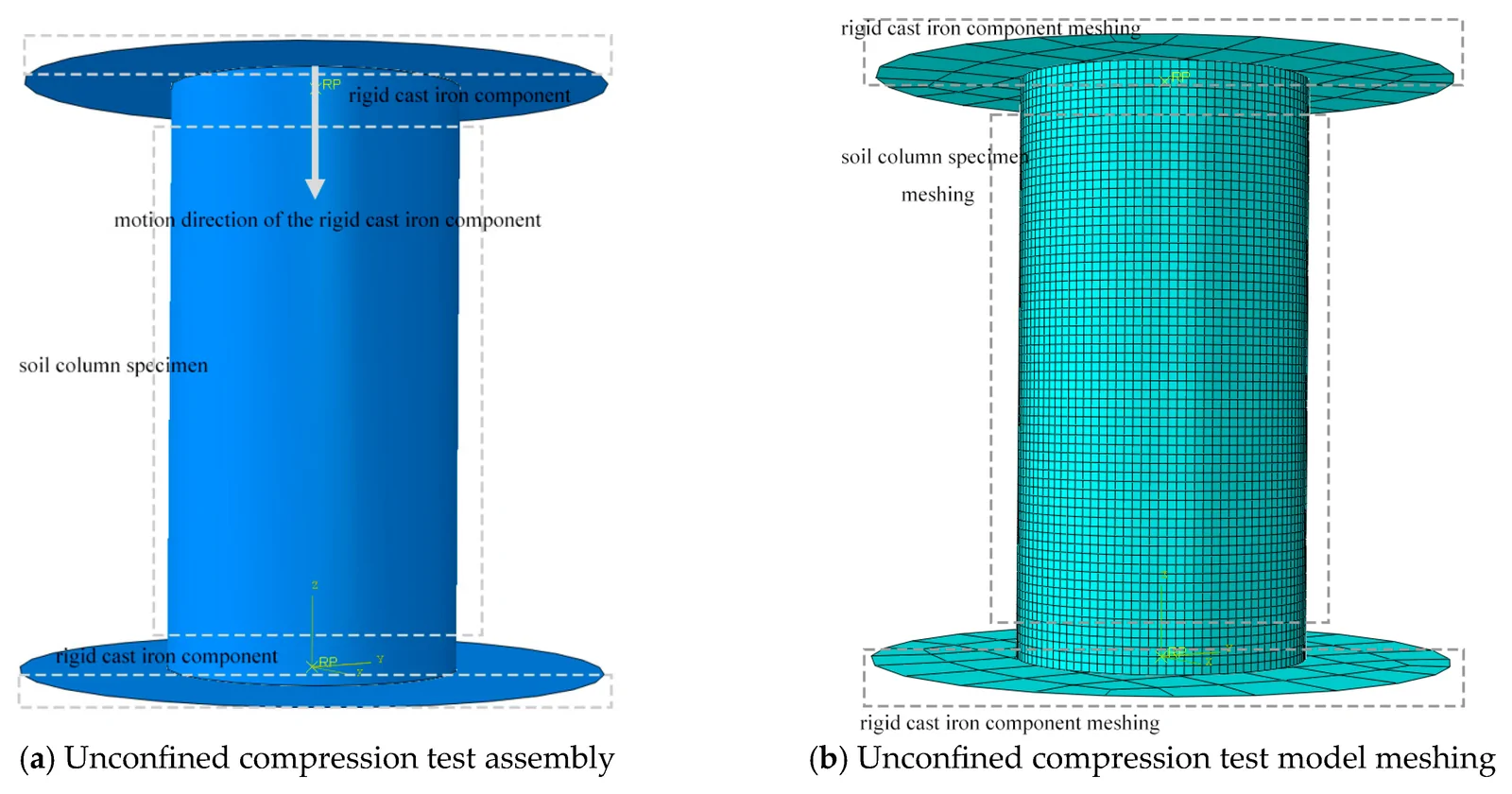
Assembly Model and Mesh Generation.

To ensure that the numerical simulations based on Abaqus accurately reflect the mechanical behavior of loess carbonation curing with different MgO contents, this study established an integrated simulation platform combining Abaqus and Fortran. User subroutines were employed to implement element failure behavior during material damage. The specific steps for model establishment and computational setup are as follows:

First, based on the experimentally measured mass growth rates of specimens with different MgO contents (10%, 15%, 20%, 25%, and 30%) under complete carbonation (1.942%, 3.326%, 4.003%, 4.226%, and 4.489%, respectively), the assumed densities of the carbonated specimens for each mix ratio were calculated. The densities reported in Table 2 were initially calculated under the assumption of constant specimen volume equal to the mold volume (V_0_ = 196.35 cm^3^), using the formula $\rho_{\mathrm{nom}}=m_{carb}/V_{0}$ , where *m_carb_* is the measured mass after carbonation. Considering the differential volume changes in specimens induced by the carbonation reaction, this study adopted a grouped modeling strategy, establishing independent geometric models for specimens within different MgO content ranges to more realistically reflect their dimensional effects. The basic physical parameters set for the plain soil and specimens with different MgO contents are as Table 2 follows:

During the model construction phase, a cylindrical geometry was adopted to represent the soil specimen, with two rigid loading plates positioned at its top and bottom. A tie constraint was applied between the plates and the soil body to simulate the close contact between the loading plates and the specimen in an actual unconfined compression test, as shown in Figure 15a below. The upper rigid plate was subjected to a constant-rate axial downward displacement, with the rate set to 1% of the specimen height per minute, to achieve quasi-static loading conditions.

To ensure computational accuracy and efficiency, structured meshing was applied to the soil domain as illustrated in Figure 15b. A swept meshing technique was employed, generating hexahedral elements using the neutral axis algorithm. A global element size of 0.0015 m was adopted for all simulations.

Mesh Sensitivity Verification: A mesh convergence study was performed for the 30% MgO specimen to verify the adequacy of the selected element size. Three mesh densities were evaluated: a coarse mesh with an element size of approximately 0.0030 m, the adopted medium mesh of 0.0015 m, and a fine mesh of approximately 0.0010 m. The simulated peak unconfined compressive strength (UCS) values for the three configurations were compared. The relative difference in peak UCS between the medium (0.0015 m) and fine (0.0010 m) meshes was less than 2.5%, indicating that the solution had effectively converged at the medium mesh density. In contrast, the coarse mesh (0.0030 m) overestimated the peak strength by approximately 8% relative to the fine mesh reference. Considering both solution accuracy and computational efficiency, the 0.0015 m element size was retained for all subsequent simulations. This configuration balances numerical stability with acceptable convergence behavior.

**Figure 16:**
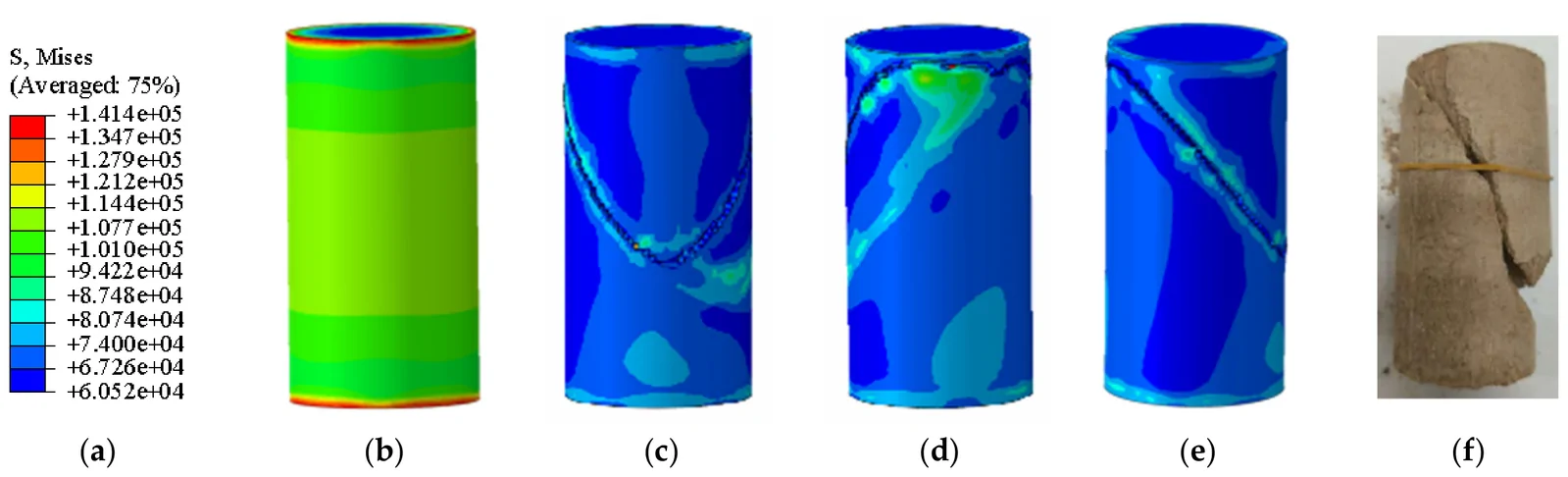
Photograph of the 10% MgO specimen and the corresponding Abaqus stress nephogram. (**a**) Abaqus Contour Plot Legend, (**b**) Elastic limit state specimen, (**c**) Specimen failure front face, (**d**) Specimen failure rear face, (**e**) Specimen failure side face, (**f**) Actual specimen photo.

**Figure 17:**
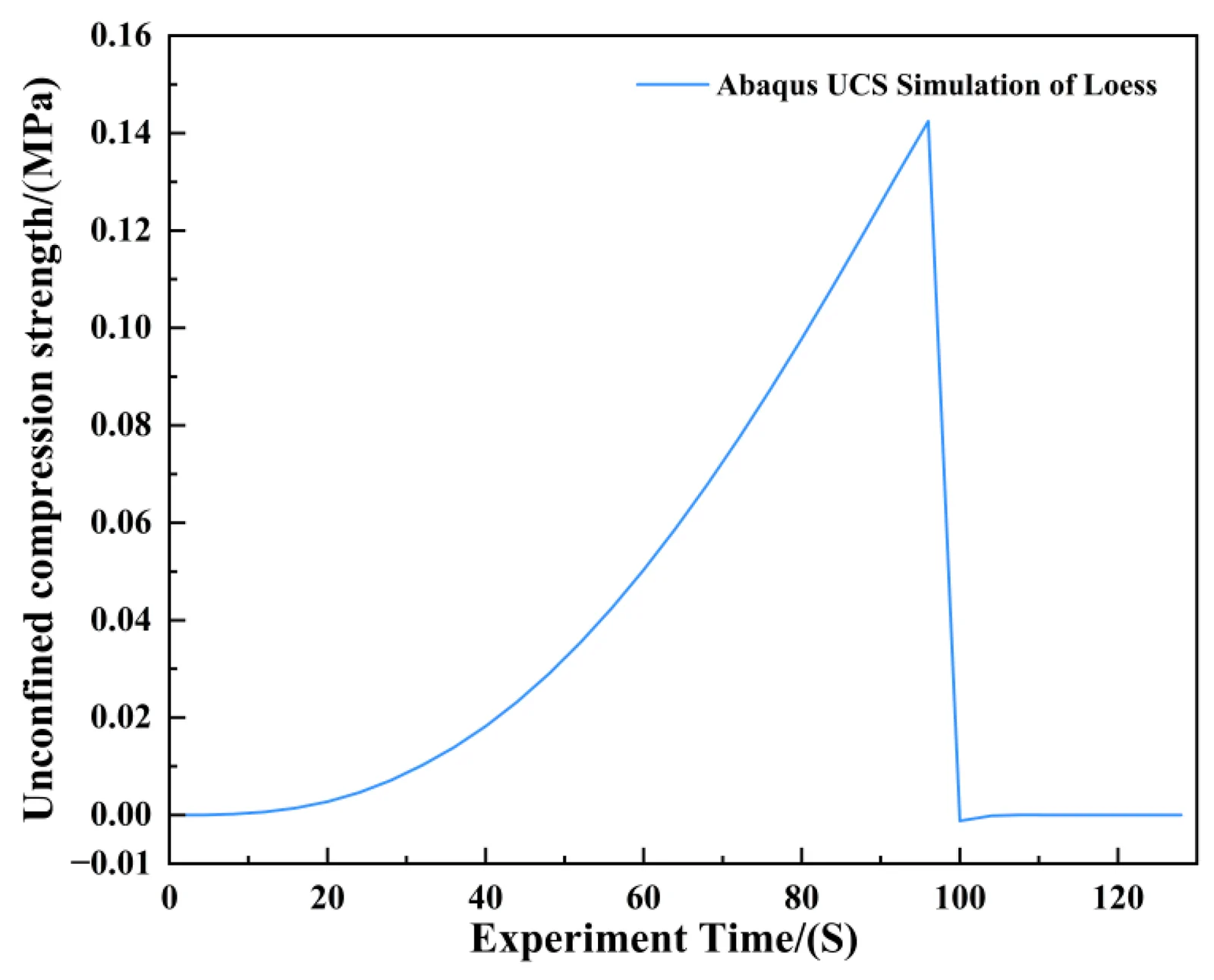
Unconfined Compressive Strength of Plain Soil.

Figure 16 presents a photograph of the unconfined uniaxial test for plain soil alongside the corresponding Abaqus stress nephogram. The stress nephogram reveals no significant stress concentration in the specimen at the critical state; however, distinct cracks developed upon exceeding this state. According to the Abaqus output, the ultimate unconfined compressive strength of the plain soil was 0.141 MPa (Figure 17). A comparison between the stress nephogram post-failure and the specimen photograph shows a large failure cross-sectional area, accounting for over 35% of the total area.

**Figure 18:**
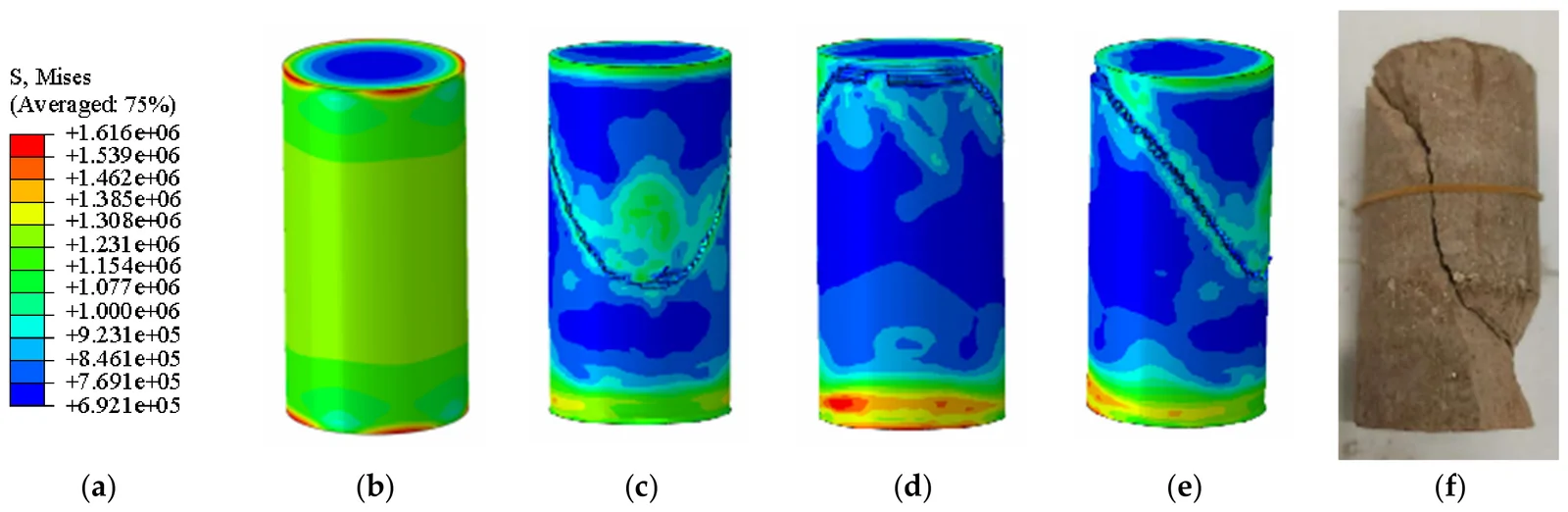
Photographs of the untreated soil specimen and the corresponding Abaqus stress contour plot. (**a**) Abaqus Contour Plot Legend, (**b**) Elastic limit state specimen, (**c**) Specimen failure front face, (**d**) Specimen failure rear face, (**e**) Specimen failure side face, (**f**) Actual specimen photo.

**Figure 19:**
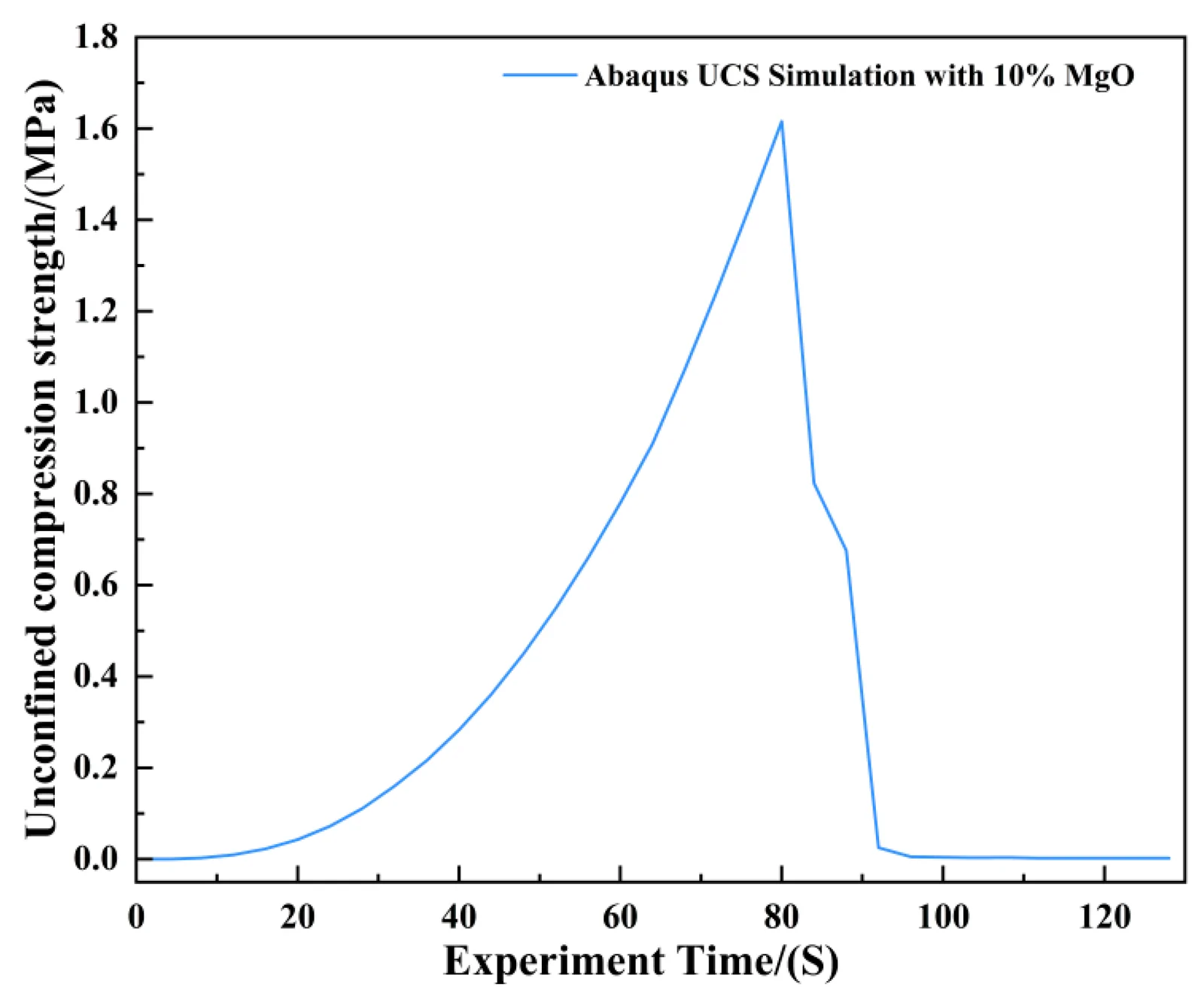
Unconfined Compressive Strength of the 10% MgO Specimen.

Figure 18 presents the photograph from the unconfined uniaxial test and the Abaqus stress nephogram for the specimen with 10% MgO content. The stress nephogram indicates that the critical and failure states of this specimen are broadly similar to those of the plain soil; however, its ultimate unconfined compressive strength is significantly higher, reaching 1.616 MPa (Figure 19). The failure cross-sectional area is substantial, accounting for over 30% of the total area.

**Figure 20:**
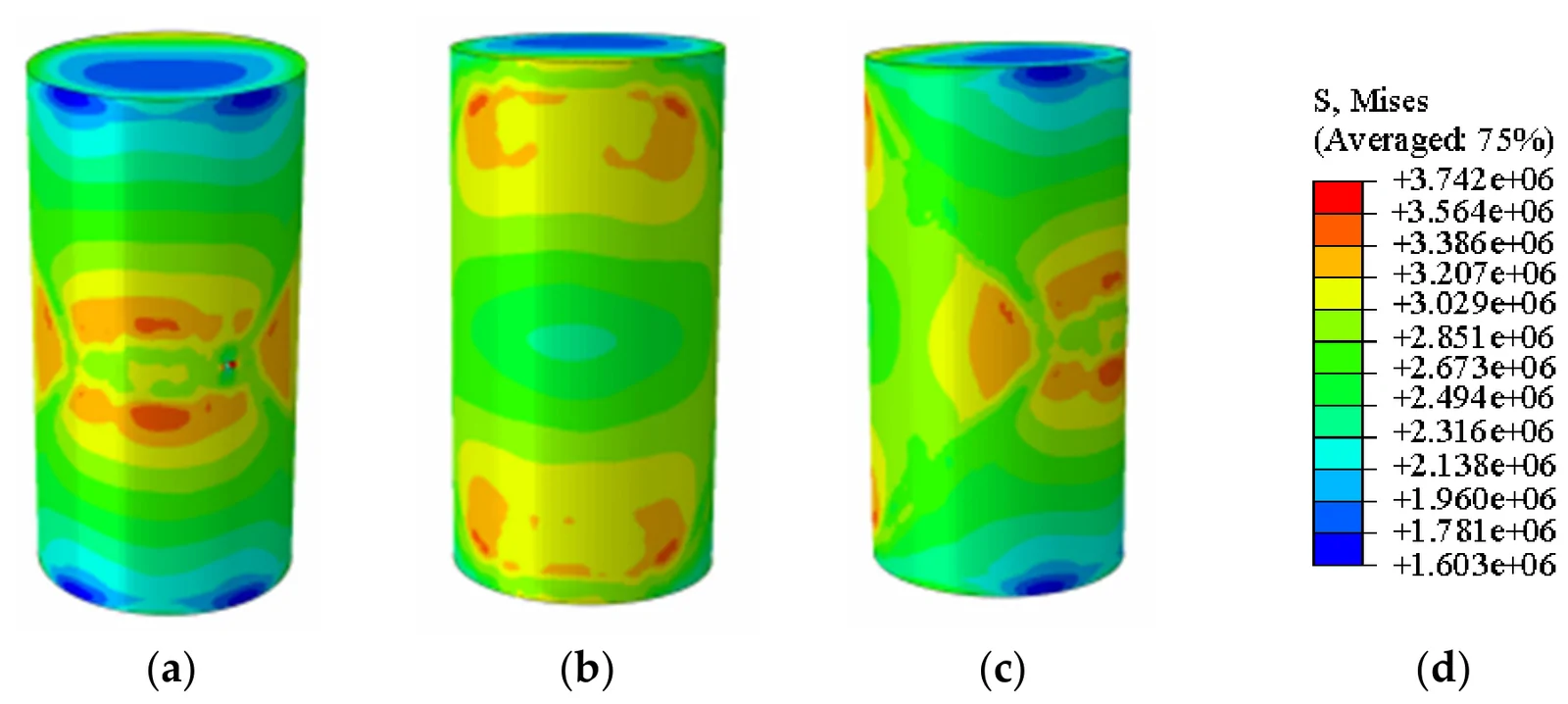
Simulated Critical State of the 15% MgO Specimen. (**a**) Critical State Front Face, (**b**) Critical State Rear Face, (**c**) Critical State Side Face, (**d**) Abaqus Contour Plot Legend.

**Figure 21:**
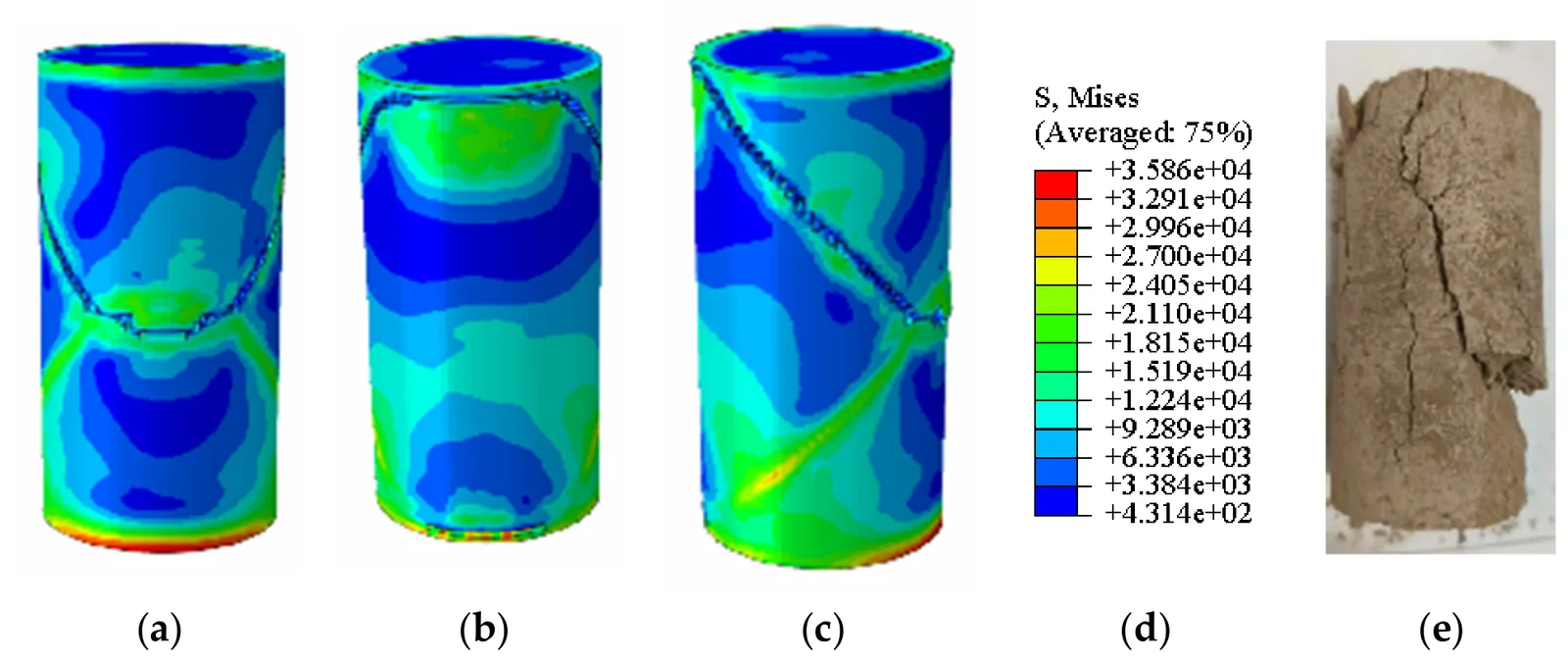
Photograph of the 15% MgO specimen and its simulated failure state. (**a**) Specimen failure front face, (**b**) Specimen failure rear face, (**c**) Specimen failure side face, (**d**) Abaqus Contour Plot Legend, (**e**) Actual specimen photo.

**Figure 22:**
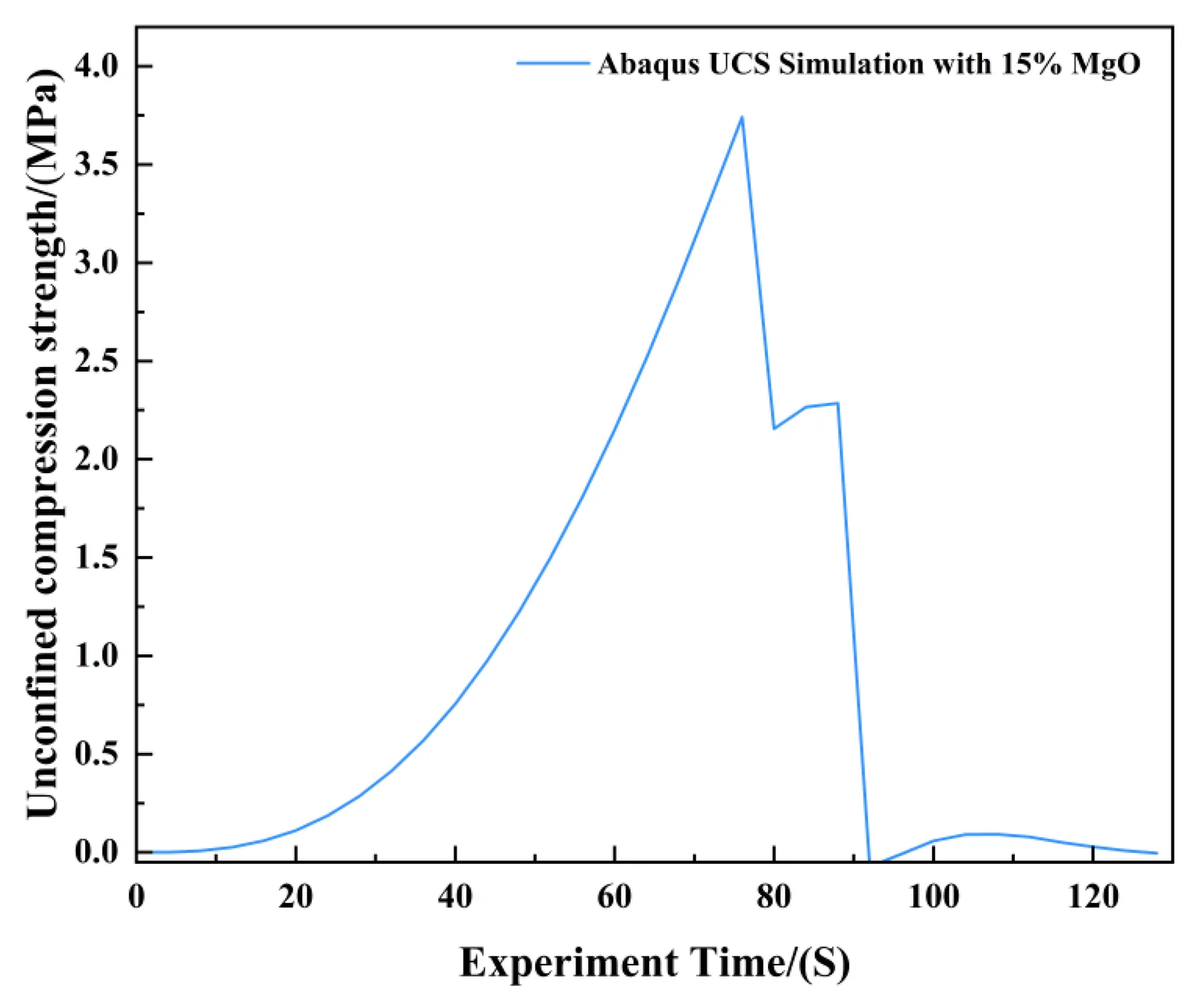
Unconfined Compressive Strength of the 15% MgO Specimen.

Figure 20 and Figure 21 present the photographic documentation of the unconfined uniaxial test and the corresponding Abaqus stress nephogram for the 15% MgO specimen. Compared to the plain soil and 10% MgO specimens, this specimen exhibits discernible stress concentration zones at the critical state, with the stress nephogram demonstrating higher symmetry. Upon failure, the upper stress concentration zone developed into a fracture surface, while the lower stress concentration zone remained as the primary load-bearing section post-fracture, showing prominent features in the nephogram. The simulation results indicate that the fracture surface is located in the middle-lower section of the specimen. The unconfined compressive strength of this specimen was determined to be 3.74 MPa (Figure 22).

**Figure 23:**
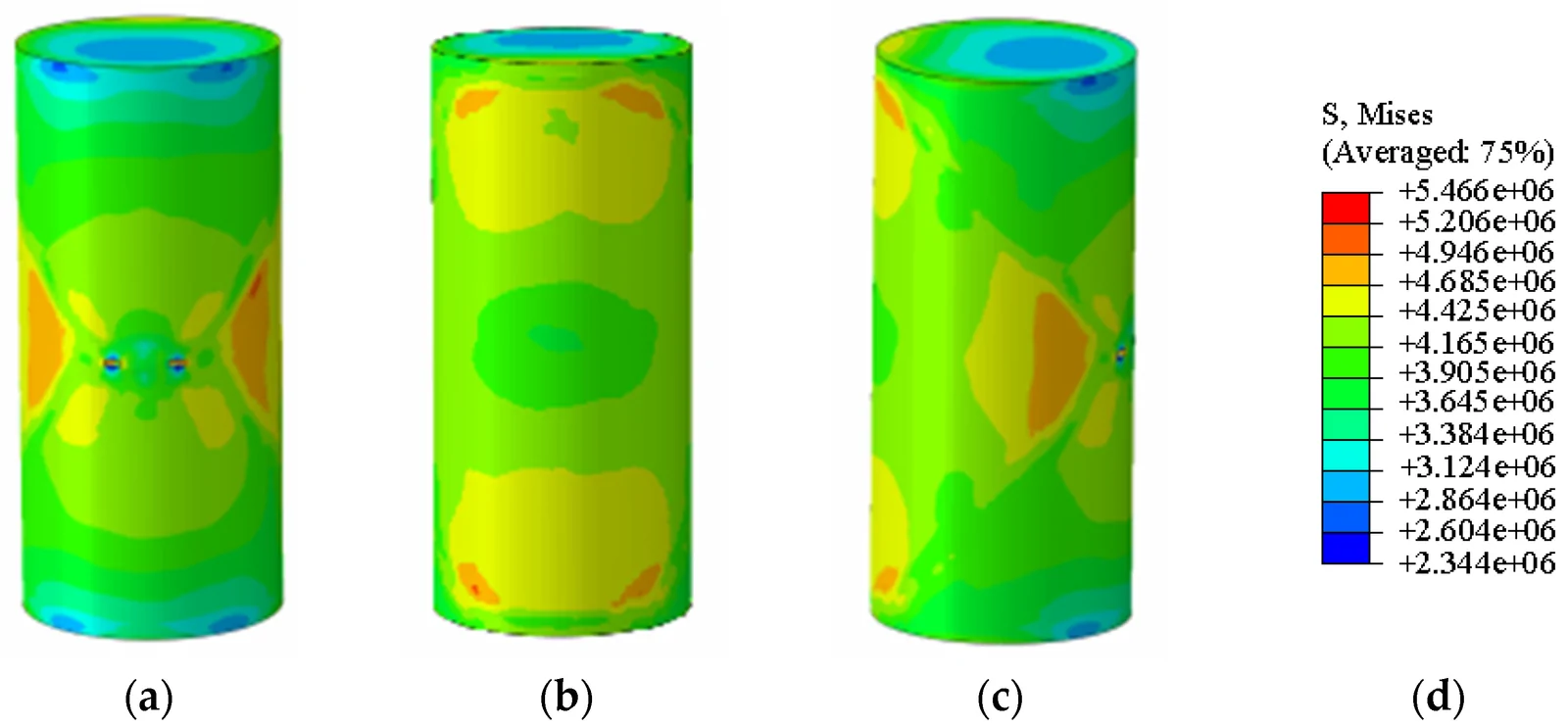
Simulated Critical State of the 20% MgO Specimen. (**a**) Critical State Front Face, (**b**) Critical State Rear Face, (**c**) Critical State Side Face, (**d**) Abaqus Contour Plot Legend.

**Figure 24:**
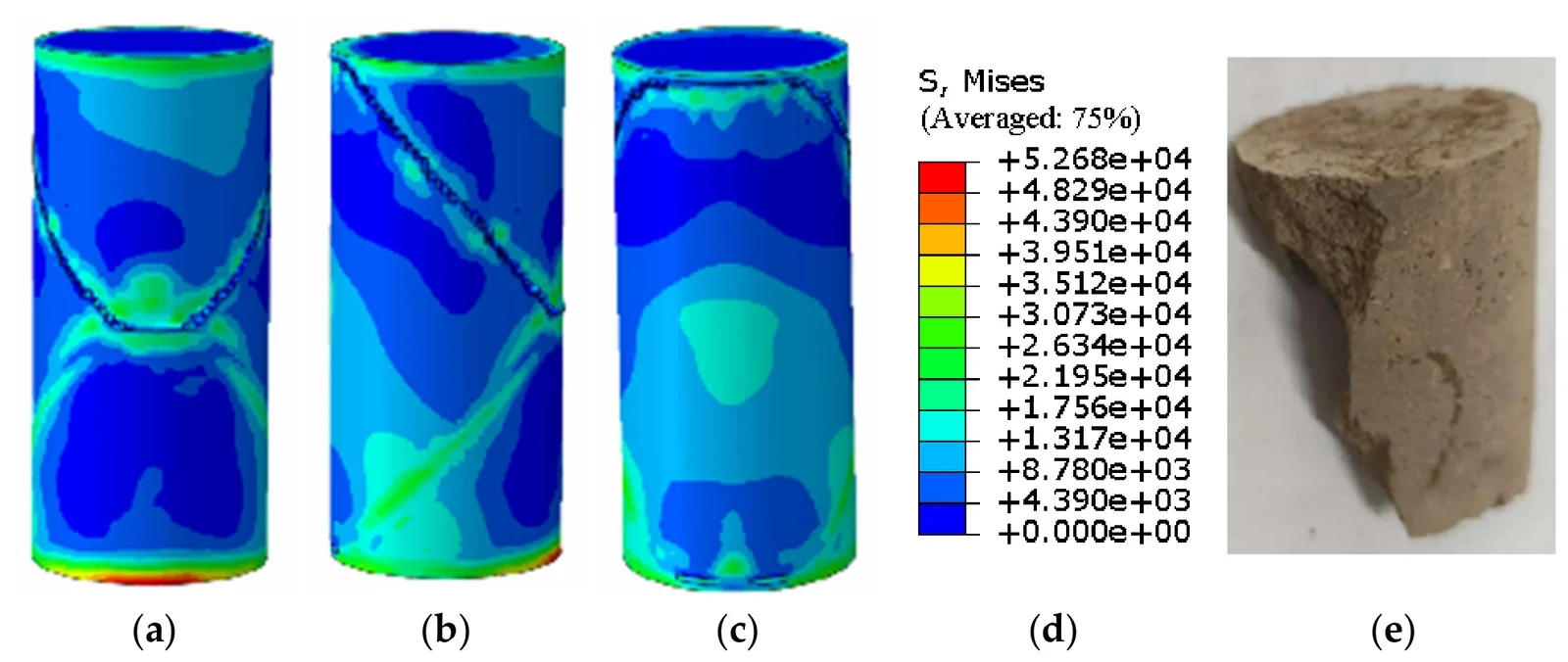
Photograph of the 20% MgO specimen and its simulated failure state. (**a**) Specimen failure front face, (**b**) Specimen failure rear face, (**c**) Specimen failure side face, (**d**) Abaqus Contour Plot Legend, (**e**) Actual specimen photo.

**Figure 25:**
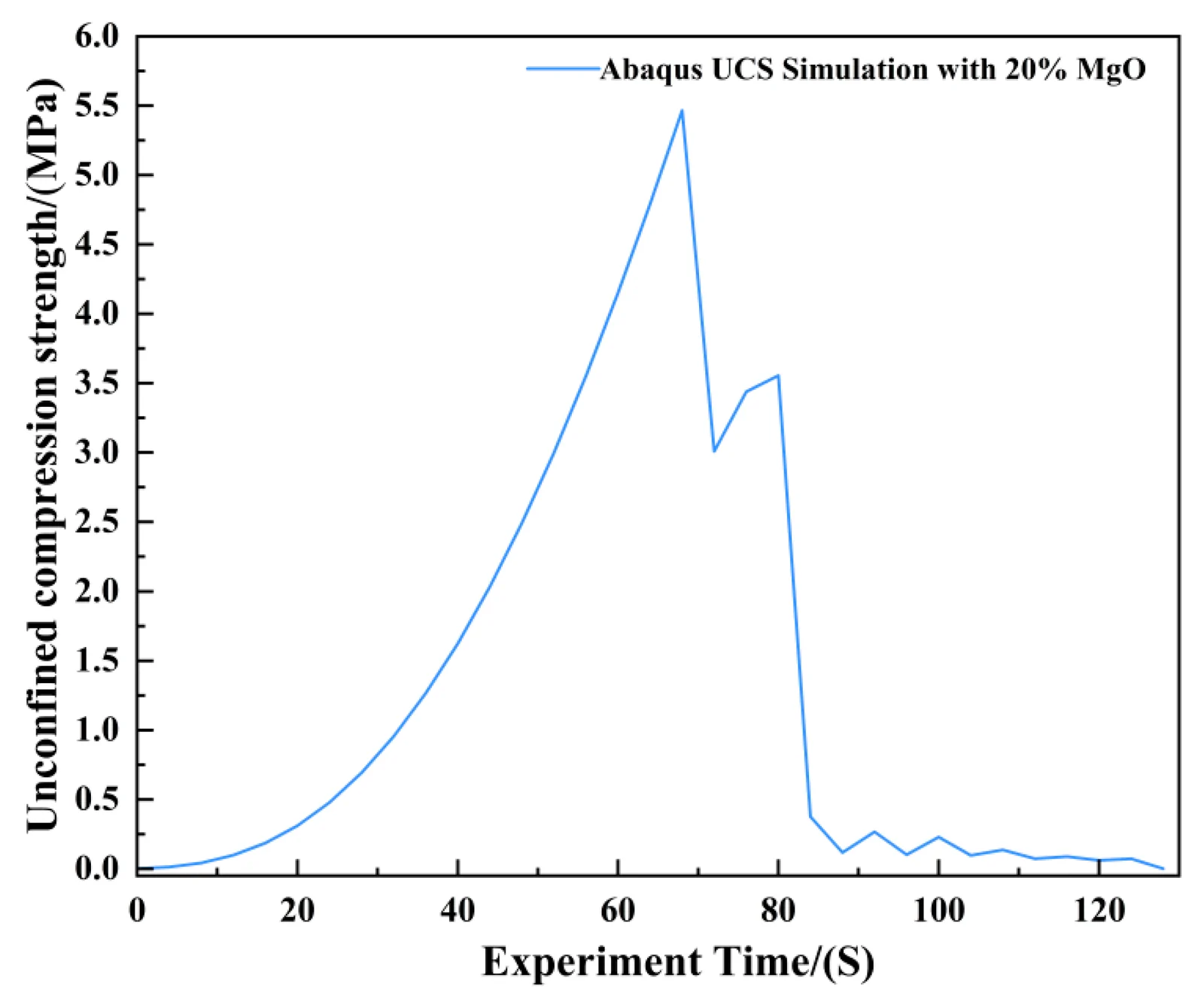
Unconfined Compressive Strength of the 20% MgO Specimen.

**Figure 26:**
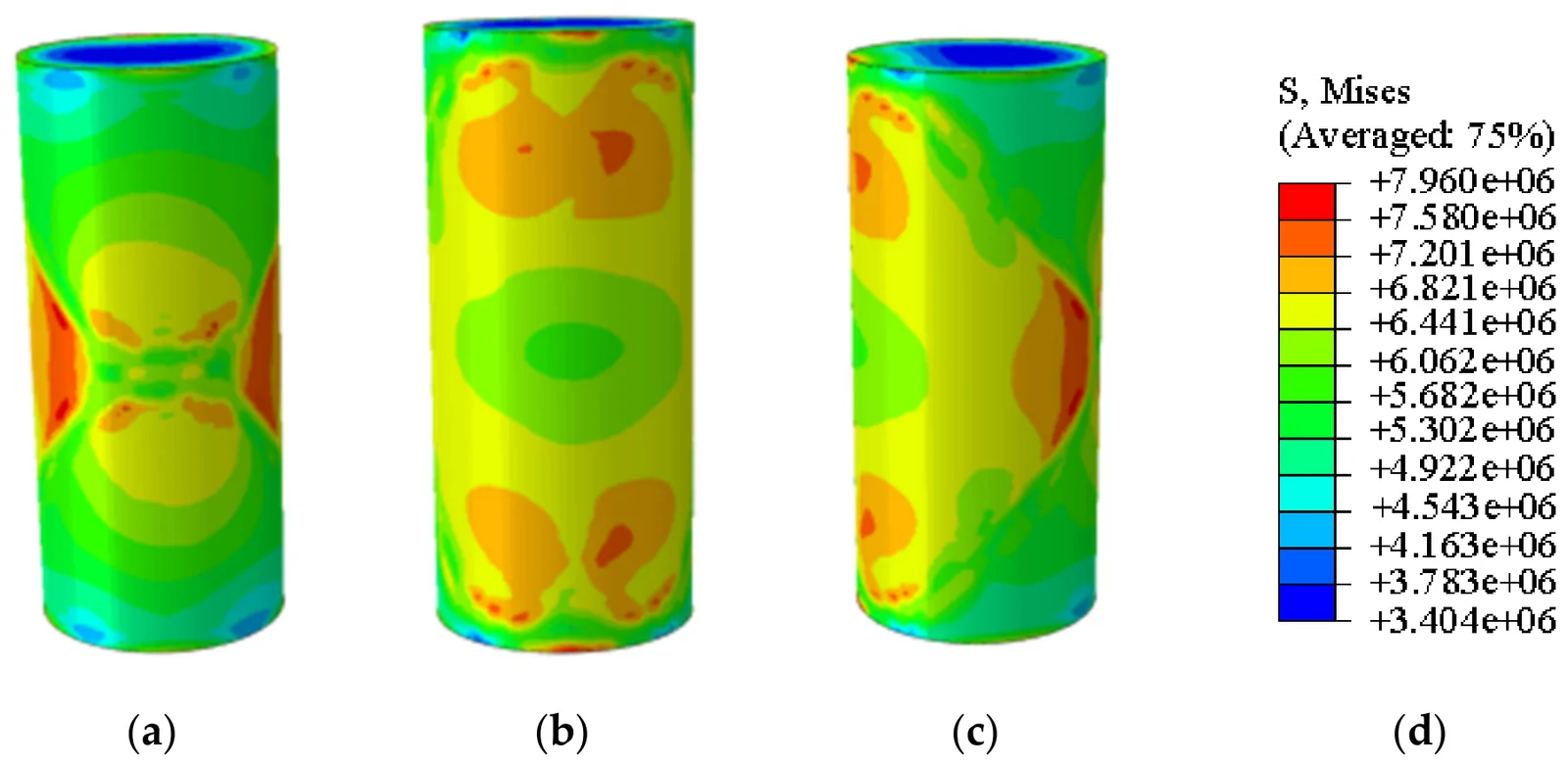
Simulated Critical State of the 25% MgO Specimen. (**a**) Critical State Front Face, (**b**) Critical State Rear Face, (**c**) Critical State Side Face, (**d**) Abaqus Contour Plot Legend.

**Figure 27:**
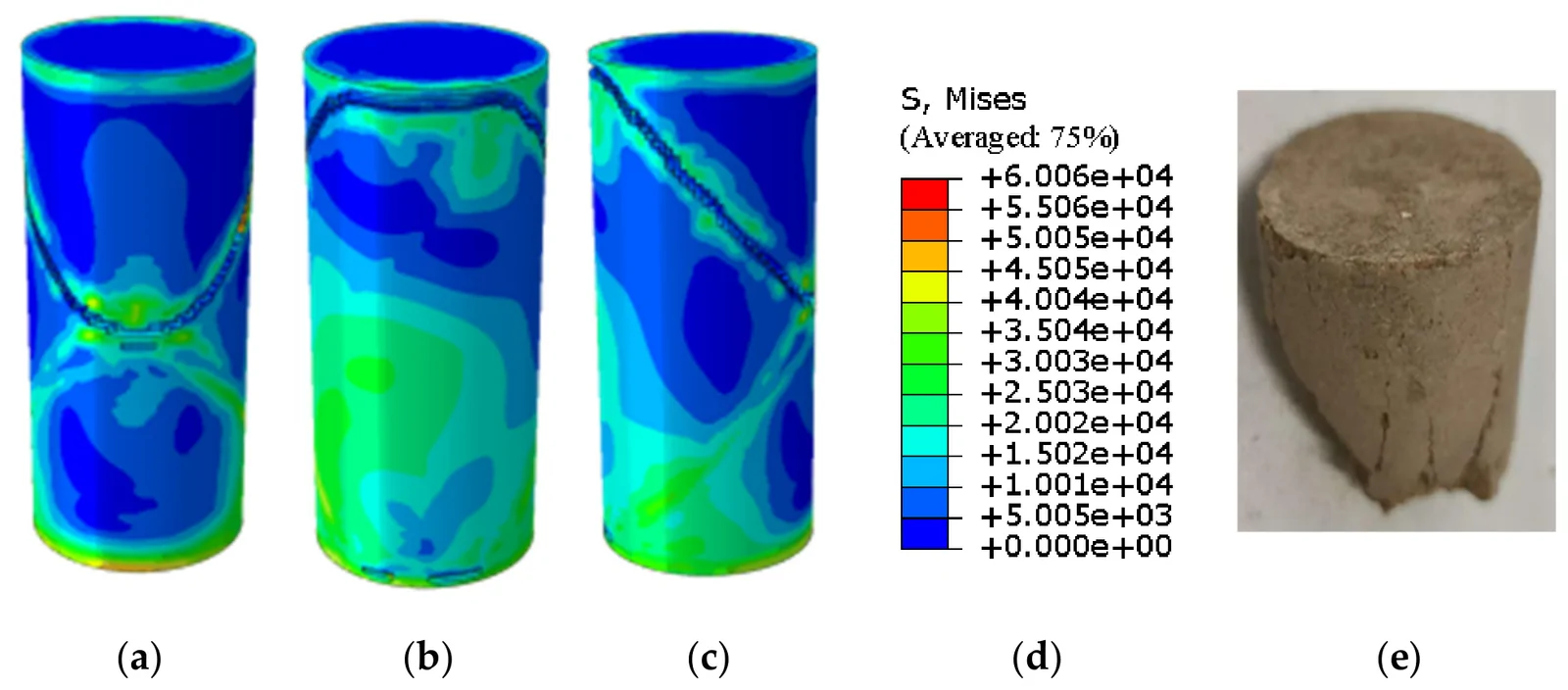
Photograph of the 25% MgO specimen and its simulated failure state. (**a**) Specimen failure front fac, (**b**) Specimen failure rear face, (**c**) Specimen failure side face, (**d**) Abaqus Contour Plot Legend, (**e**) Actual specimen photo.

**Figure 28:**
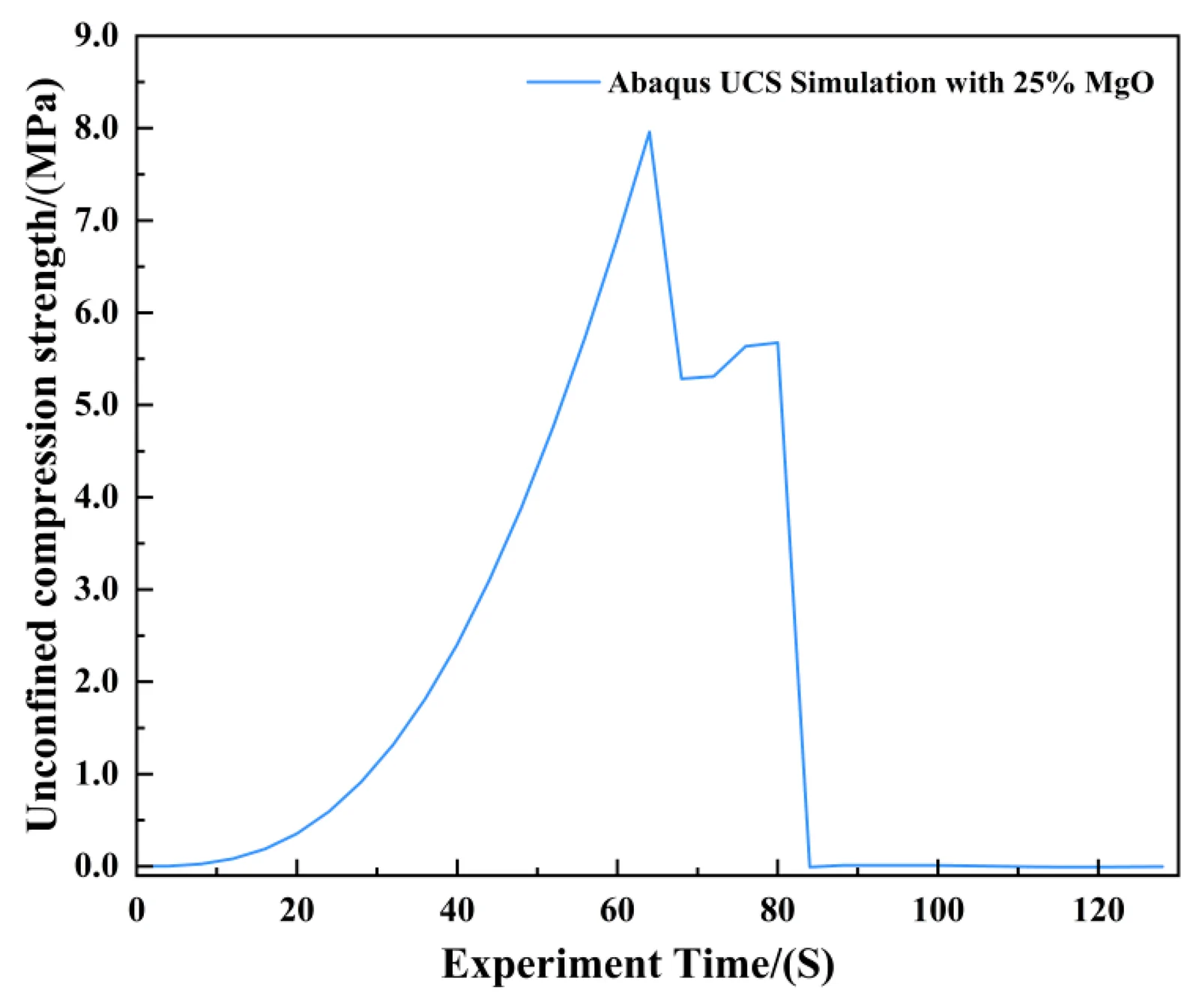
Unconfined Compressive Strength of the 25% MgO Specimen.

**Figure 29:**
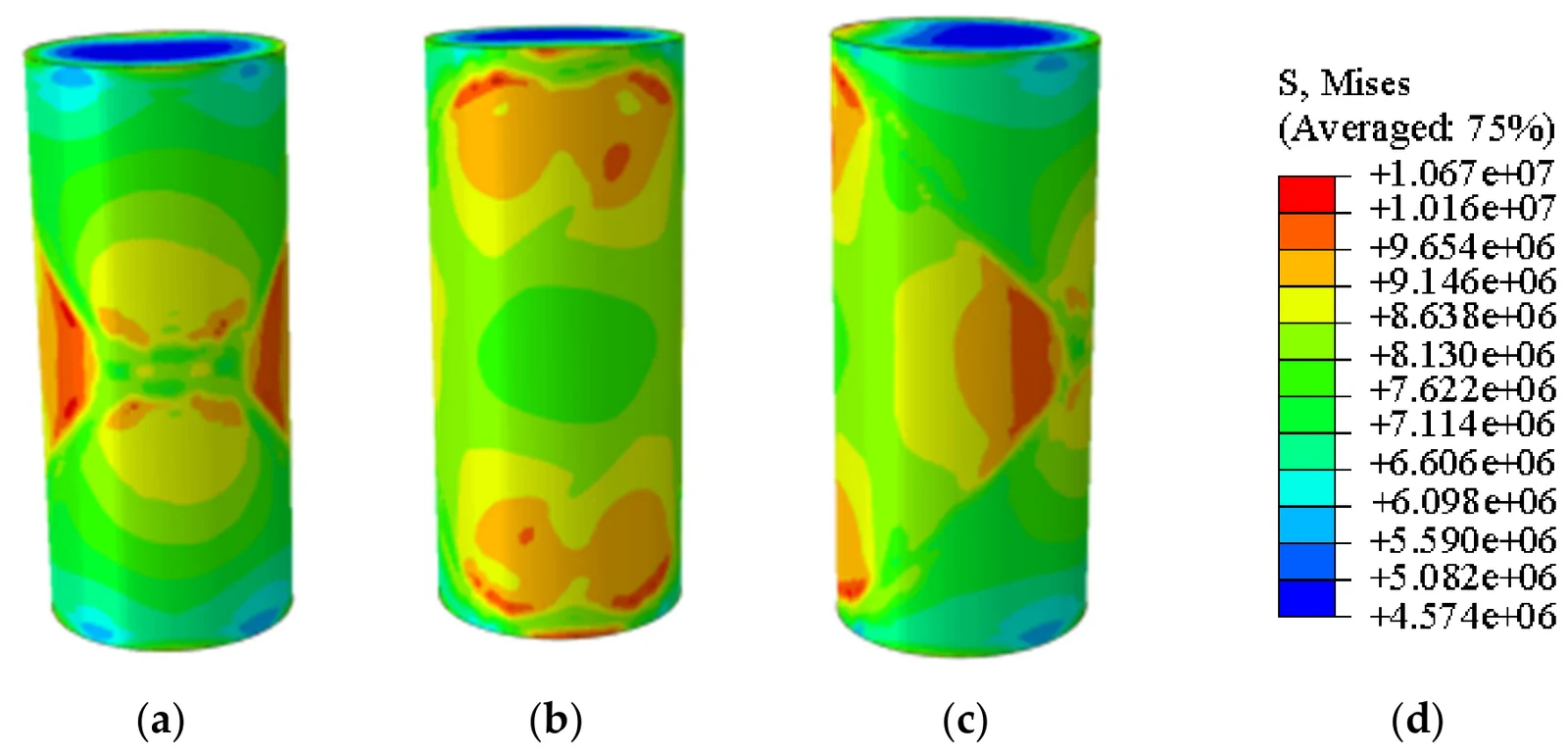
Simulated Critical State of the 30% MgO Specimen. (**a**) Critical State Front Face, (**b**) Critical State Rear Face, (**c**) Critical State Side Face, (**d**) Abaqus Contour Plot Legend.

**Figure 30:**
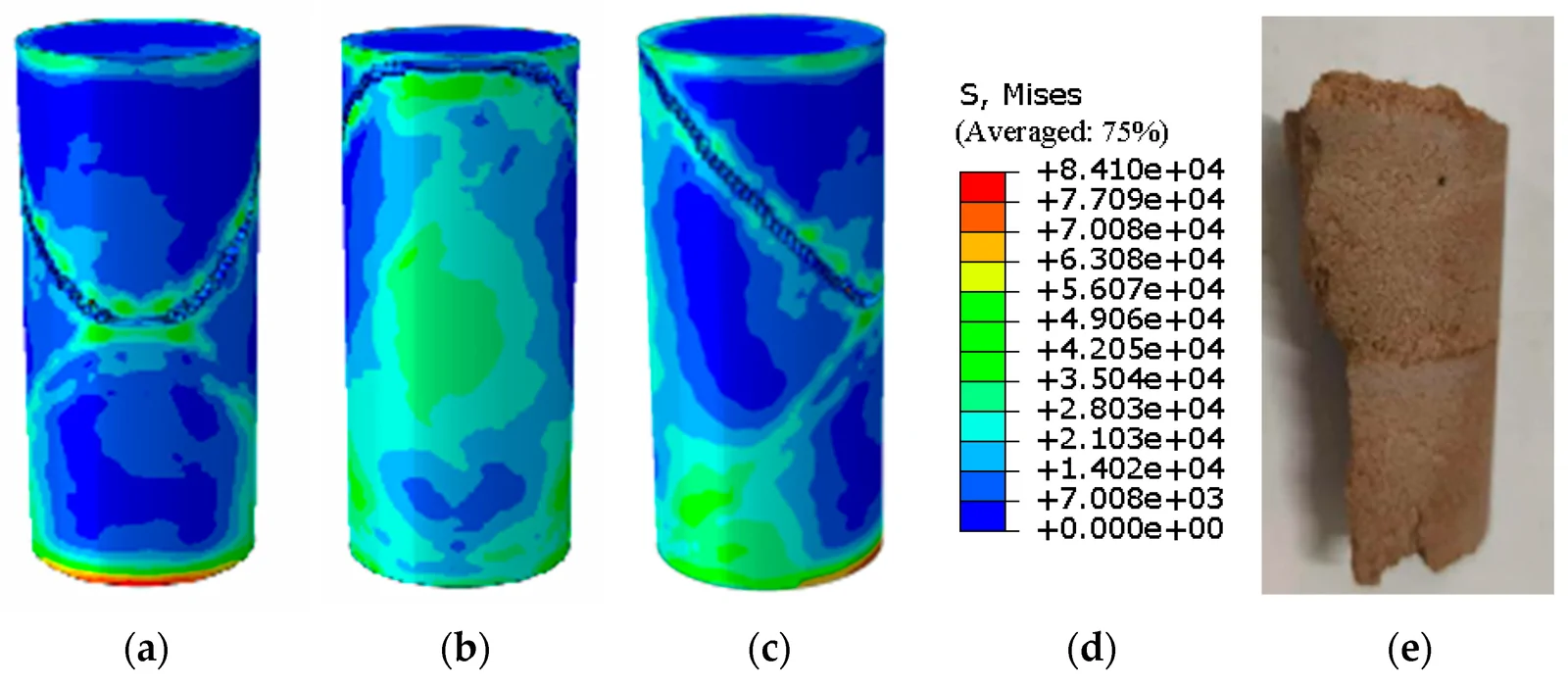
Photograph of the 30% MgO specimen and its simulated failure state. (**a**) Specimen failure front face, (**b**) Specimen failure rear face, (**c**) Specimen failure side face, (**d**) Abaqus Con-tour Plot Legend, (**e**) Actual specimen photo.

**Figure 31:**
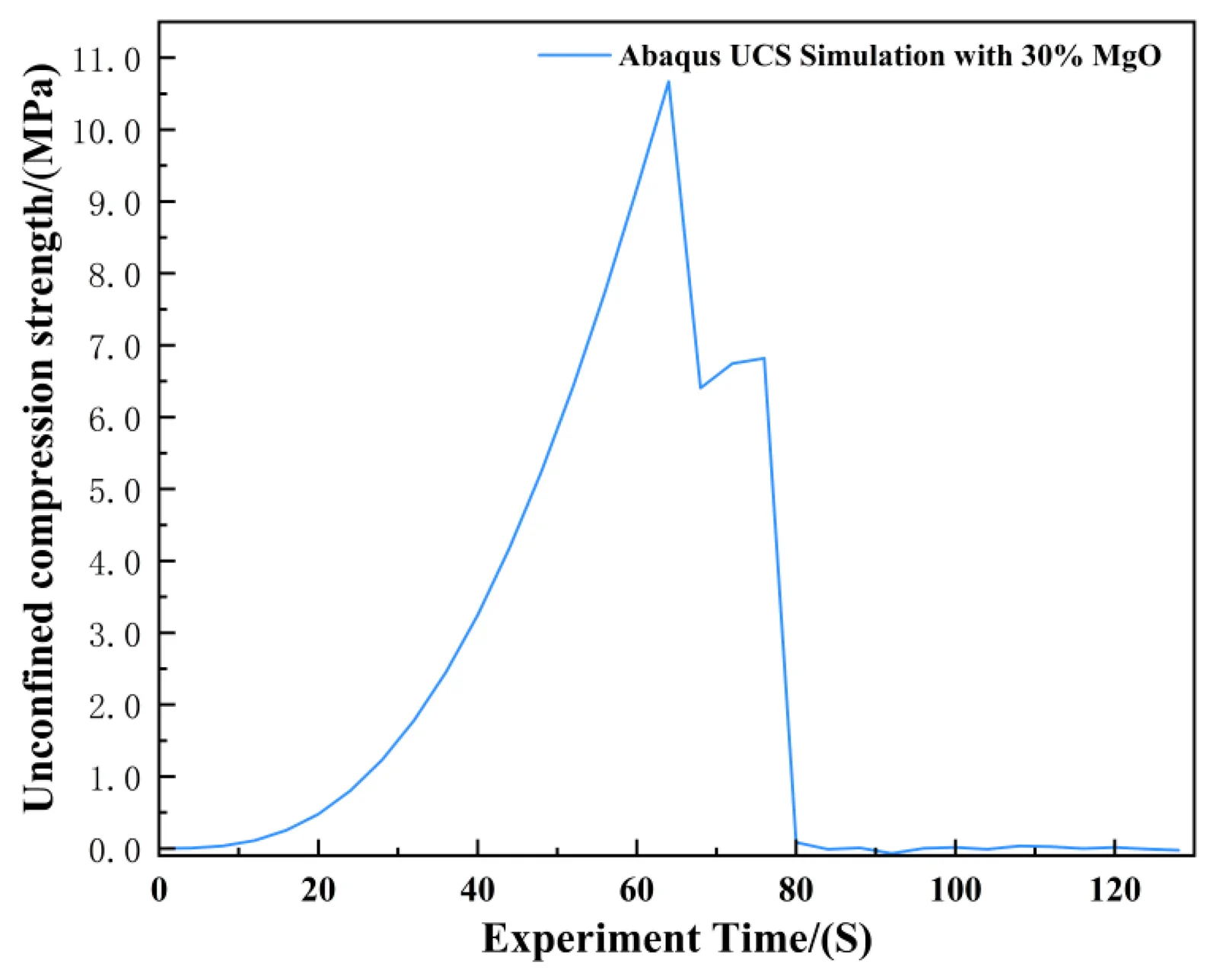
Unconfined Compressive Strength of the 30% MgO Specimen.

The agreement between the Abaqus-simulated UCS values and the experimental measurements was evaluated quantitatively (Figure 23, Figure 24, Figure 25, Figure 26, Figure 27, Figure 28, Figure 29, Figure 30 and Figure 31). For the 10%, 15%, 20%, 25%, and 30% MgO specimens, the simulated peak UCS values were 1.616 MPa, 3.742 MPa, 5.466 MPa, 7.960 MPa, and 10.670 MPa, respectively. The corresponding experimental values were 1.568 MPa, 3.681 MPa, 5.388 MPa, 7.939 MPa, and 10.478 MPa. The relative errors between simulation and experiment were +3.06%, +1.66%, +1.45%, +0.26%, and +1.83%, respectively, yielding a mean absolute percentage error (MAPE) of 1.65% (Table 3). This close quantitative correspondence confirms that the calibrated finite element model accurately reproduces the measured UCS response across the full range of MgO dosages investigated.

**Figure 32:**
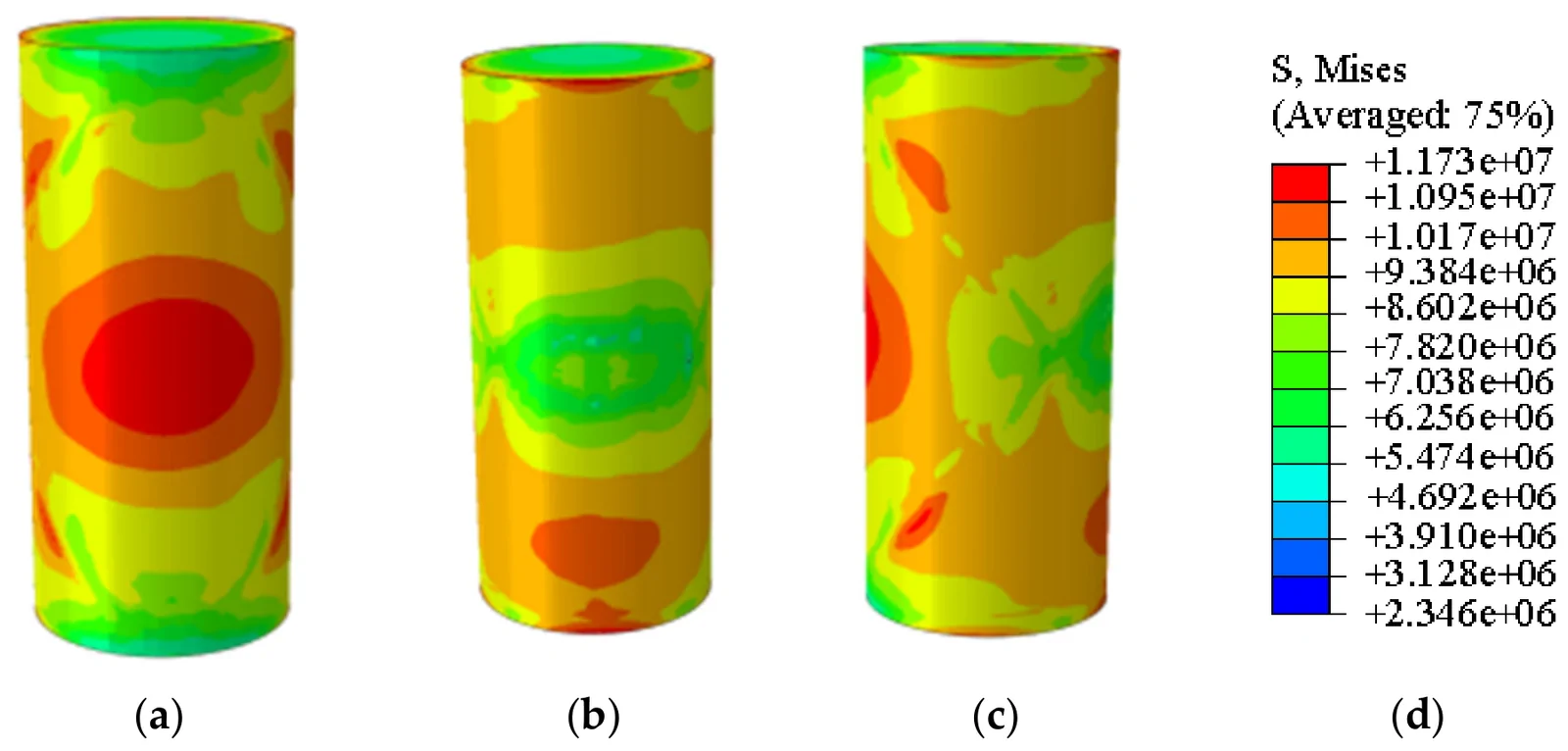
Simulated Critical State of the 35% MgO Specimen. (**a**) Critical State Front Face, (**b**) Critical State Rear Face, (**c**) Critical State Side Face, (**d**) Abaqus Contour Plot Legend.

**Figure 33:**
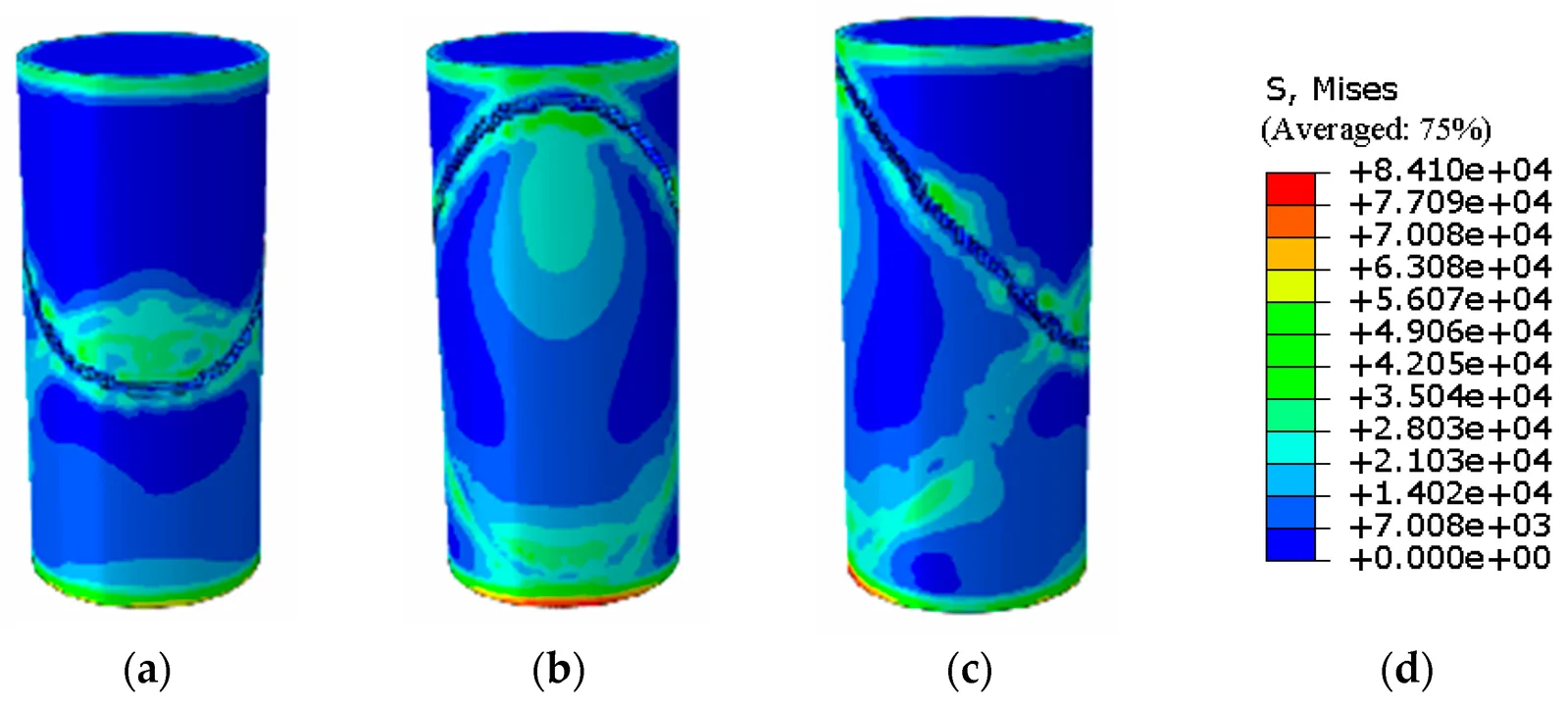
Simulated Failure State of the 35% MgO Specimen. (**a**) Specimen failure front face, (**b**) Specimen failure rear face, (**c**) Specimen failure side face, (**d**) Abaqus Contour Plot Legend.

**Figure 34:**
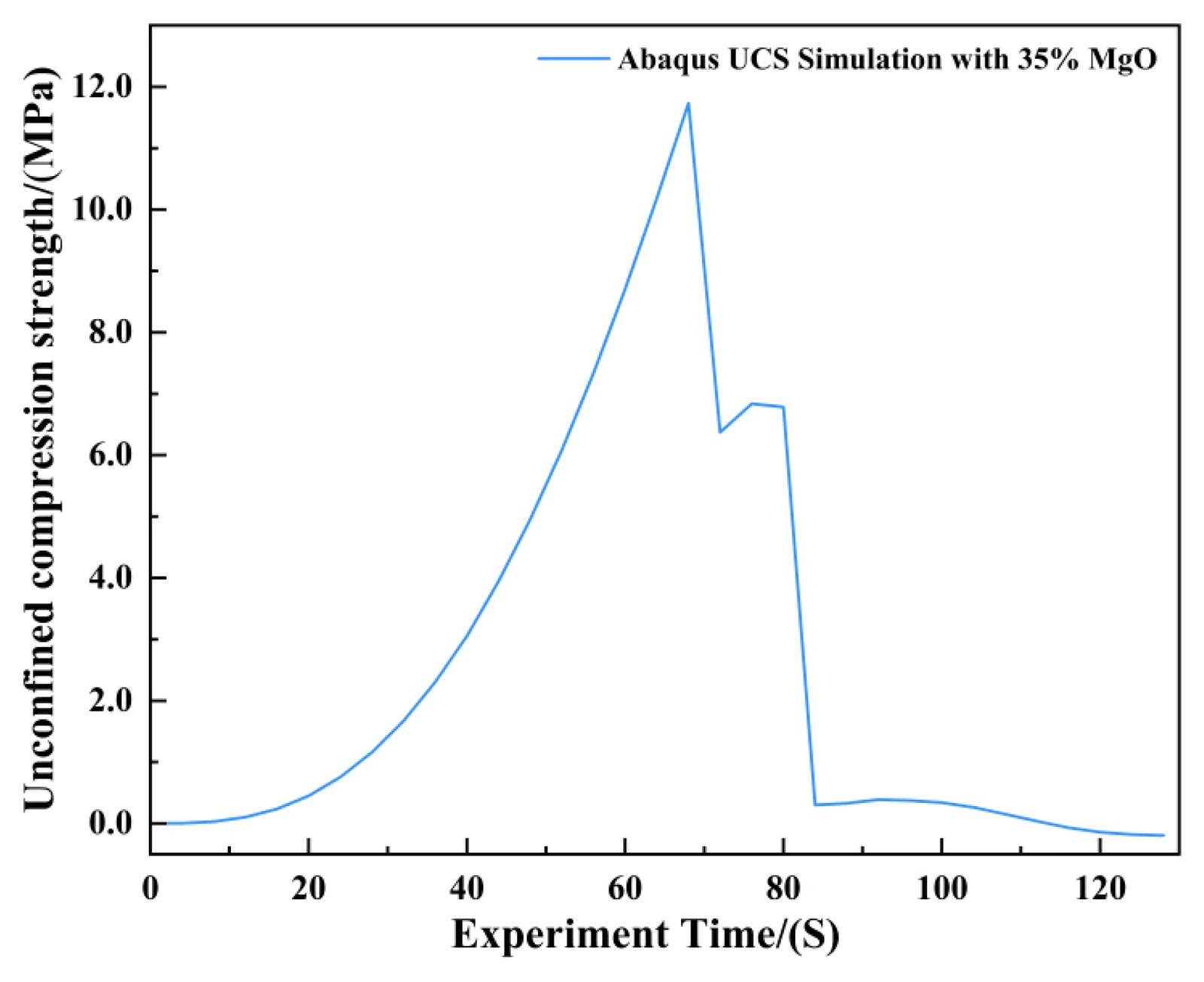
Unconfined Compressive Strength of the 35% MgO Specimen.

In the Abaqus simulation results for the specimen with 35% MgO content, as shown in Figure 32 and Figure 33, more pronounced stress concentration phenomena and a distinct symmetrical distribution pattern were observed, while the unconfined compressive strength reached 11.732 MPa (Figure 34).

**Figure 35:**
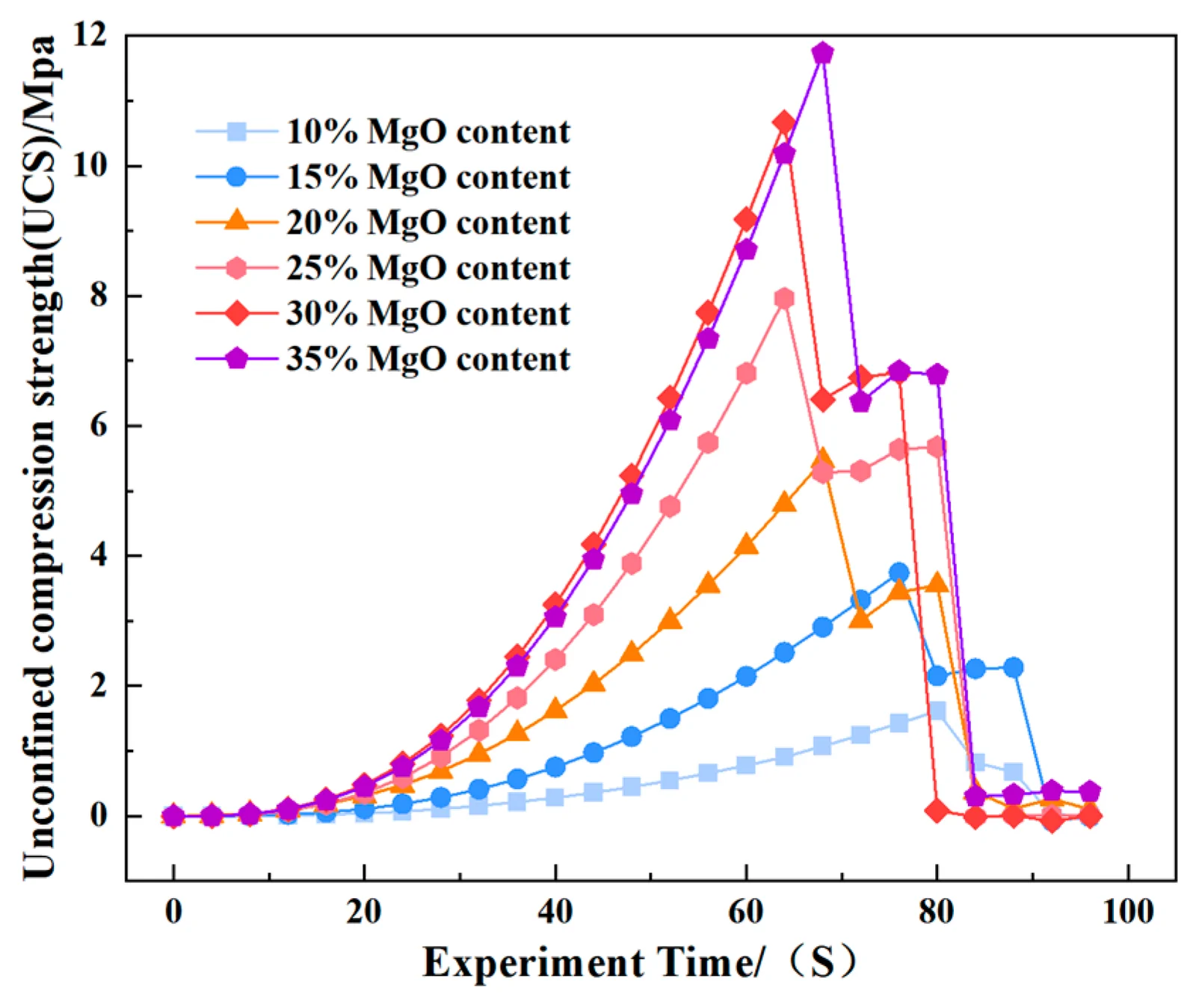
Abaqus-simulated unconfined compressive strength.

As shown in Figure 35, the Abaqus simulation results of unconfined compressive strength indicate that specimens with higher MgO content exhibit smaller strain while sustaining greater stress. Localized failure occurs in specimens with MgO contents ranging from 15% to 35%, whereas complete failure is observed in plain soil and low-content (10%) specimens. Experimental photographs further confirm that higher MgO specimens experience localized failure; however, axial compression continues thereafter, resulting in incomplete final fracture profiles.

### 3.6. Analysis of Compressive Strength and Microstructural Evolution of Soil Samples with Different MgO Contents

This study systematically evaluates the evolution of mechanical properties and carbonation-induced solidification effects in stabilized collapsible loess under varying MgO contents by integrating macroscopic mechanical testing with microstructural characterization. Unconfined compressive strength (UCS) parameters were collected at critical carbonation intervals (0 h, 12 h, and 24 h), and the observed trends in strength development were correlated with corresponding variations in mineral phase composition and microstructure. This parallel macro–micro analysis elucidates the mechanistic linkages between MgO dosage, reaction product evolution, and the resulting engineering performance of the stabilized soil.

Microstructural characterization was conducted using multi-scale analytical techniques: X-ray diffraction was employed to accurately identify the crystalline phase composition and diffraction intensity distribution of carbonation products [29]; field emission scanning electron microscopy was utilized to observe fracture surface morphology and quantitatively analyze the phase transformation pathways of magnesium-based minerals and the evolution of their spatial topological structures.

**Figure 36:**
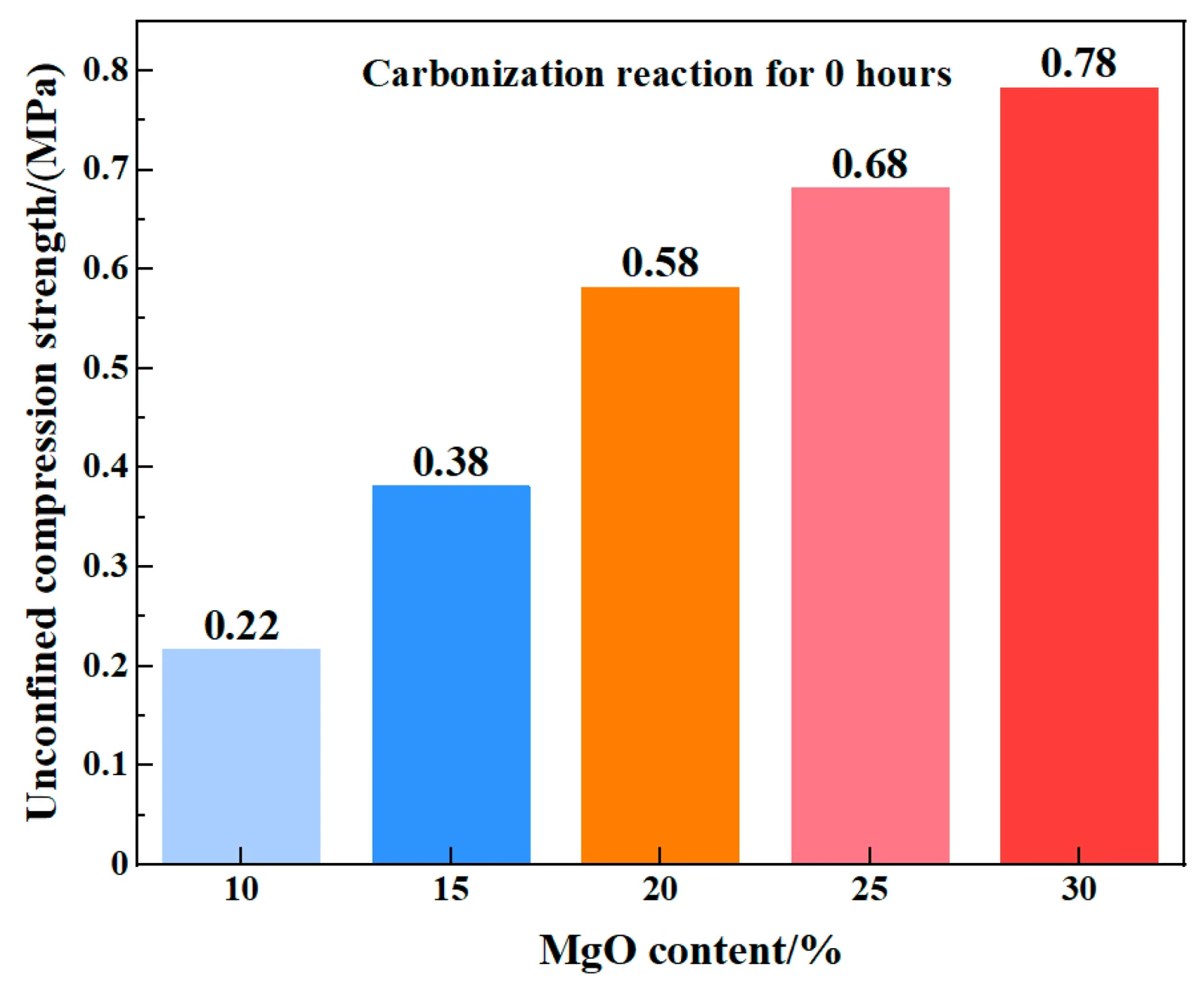
Unconfined compressive strength of samples with different MgO contents at 0 h.

Figure 36 presents experimental results of unconfined compressive strength at the initial carbonation stage. It is clearly observed that significant differences in strength exist between low MgO content samples (10–15% and 15–20%), while higher MgO content samples (20–25% and 25–30%) exhibit a distinct stepped pattern of strength progression. The incremental difference between these steps is approximately 0.1 MPa, showing a significant positive correlation with the mass growth rate.

**Figure 37:**
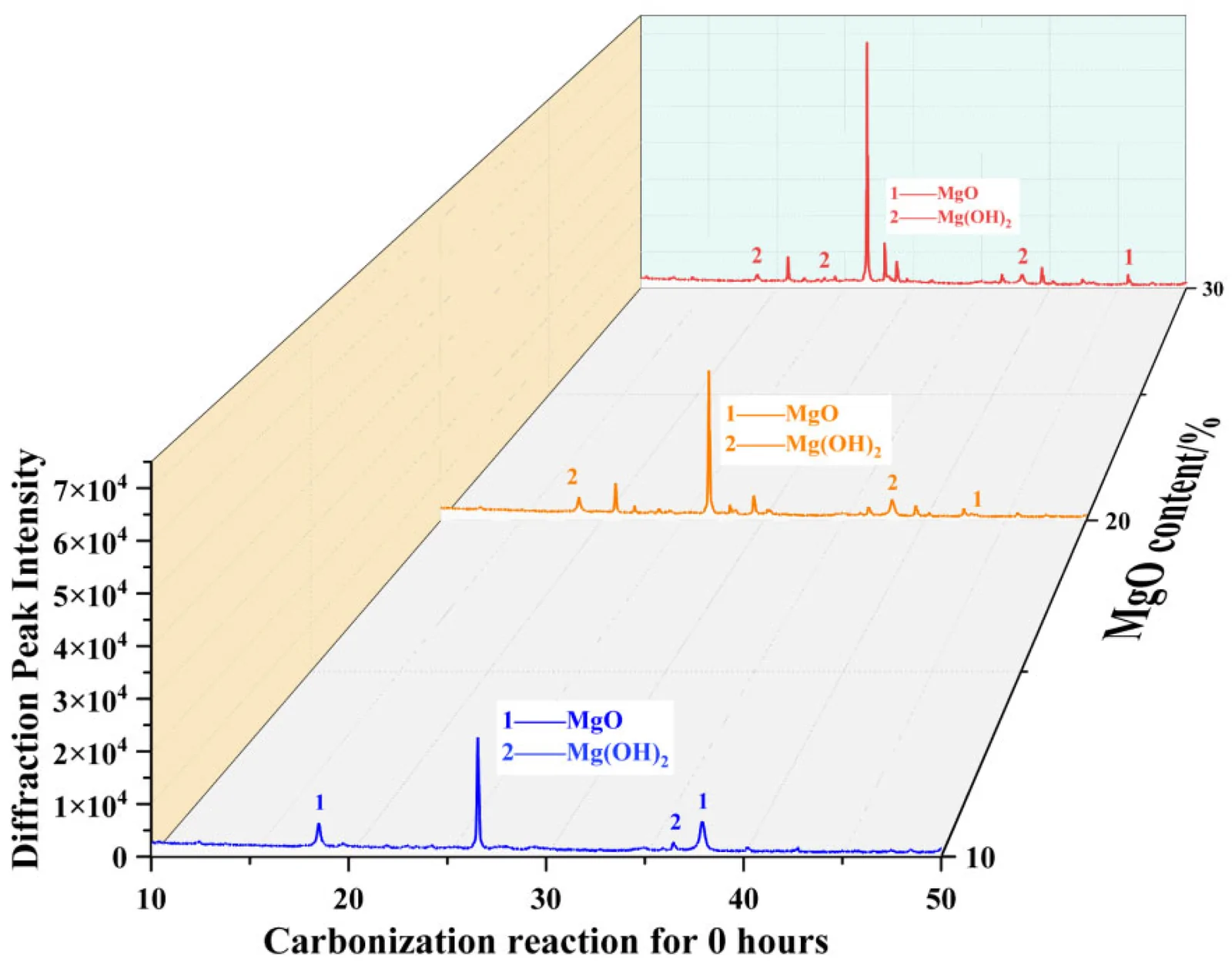
XRD analysis results of samples with different MgO contents at 0 h.

Quantitative X-ray diffraction (XRD) analysis revealed that the specimen with 30% MgO content exhibited the most pronounced multiphase mineral characteristics under uncarbonated conditions (Figure 37). Its diffraction pattern displayed four characteristic peaks, three of which were strong and closely matched the crystallographic planes of magnesium hydroxide. The relative peak intensities were significantly higher compared to those of the 10% MgO specimen.

This observation indicates that increased MgO content markedly promotes the formation of magnesium hydroxide within the system and facilitates heterogeneous nucleation of magnesium-based minerals through an ion migration-precipitation mechanism. Microstructural characterization further confirmed that the cementing effect of magnesium hydroxide, together with the three-dimensional interwoven crystalline framework formed with other magnesium-based minerals, effectively filled the pores. This microstructural evolution collectively constitutes the mechanistic basis for the mechanical enhancement, ultimately demonstrating a dose-dependent relationship between unconfined compressive strength and MgO content.

**Figure 38:**
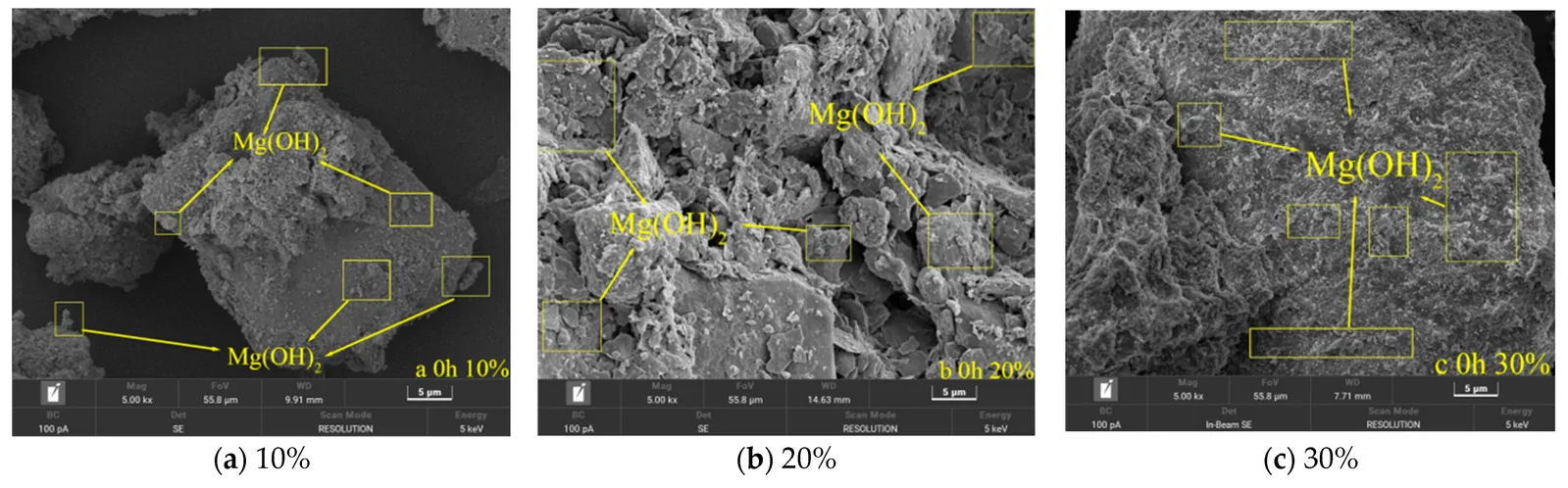
SEM Scanning Results of Different MgO Samples after Carbonization for 0 h.

Morphological characterization by scanning electron microscopy (SEM) (Figure 38) revealed that at the initial carbonation stage (0 h), uncarbonated specimens were primarily composed of flake-like magnesium hydroxide [30], the apparent volume fraction of which increased with MgO content. In the high-MgO system (30% MgO), the Mg(OH)_2_ crystals exhibited pronounced aggregation, forming dense spherical clusters approximately 2–5 μm in diameter [31]. Isolated, discrete crystals constituted only a minor fraction of the observed microstructure in these specimens. In contrast, the low-MgO specimen (10% MgO) was dominated by loosely distributed hexagonal plate-like crystals with minimal aggregation.

This distinct difference in microscale morphology suggests that higher MgO contents promote the formation of a more continuous and interlocked structural framework even before the onset of carbonation. Correlating these observations with the UCS measurements (Figure 36) indicates that the presence of well-developed Mg(OH)_2_ clusters contributes positively to the early-age mechanical performance of the stabilized soil, primarily through enhanced particle interlocking and a reduction in localized porosity within the loess matrix.

Taken together, the XRD phase identification and UCS measurements indicate that the presence of well-developed Mg(OH)_2_ clusters enhances the mechanical response of the stabilized soil. This improvement is attributed to the combined effects of increased interfacial bonding area, reduced localized porosity [32], and the establishment of a load-bearing skeletal network within the loess matrix.

**Figure 39:**
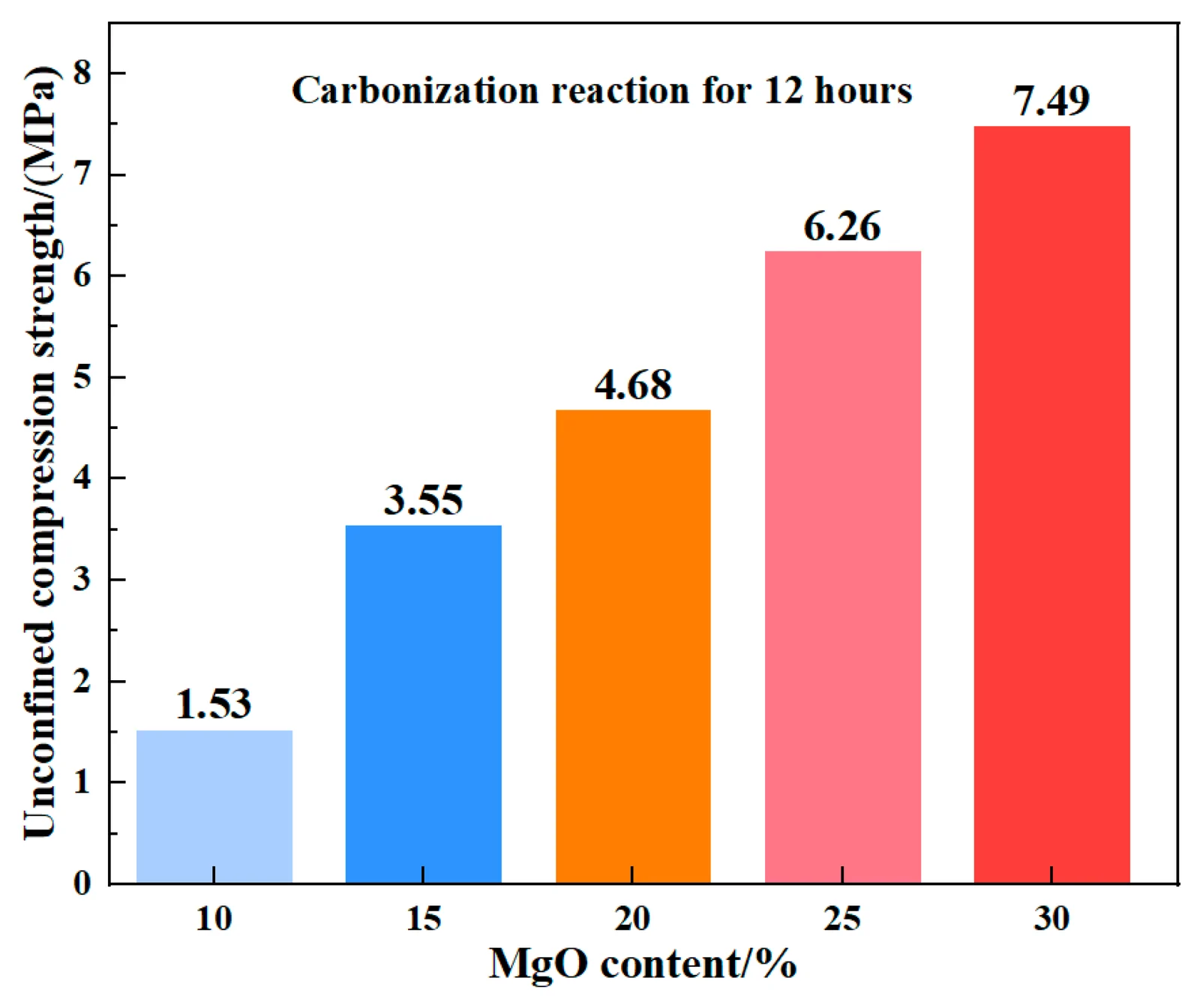
Unconfined compressive strength of samples with different MgO contents at 12 h.

In the 12 h unconfined compressive strength tests, the compressive strength continued to increase with higher MgO content (Figure 39). Unlike the 0 h results, specimens with MgO contents above 15% exhibited a distinct stepped progression in strength, while the maximum strength difference between the 10% and 15% MgO samples reached 2.02 MPa. This behavior can be attributed to the progression of carbonation: under 12 h of carbonation, the 10% MgO specimen had nearly reached complete carbonation, resulting in a limited further increase in unconfined compressive strength.

**Figure 40:**
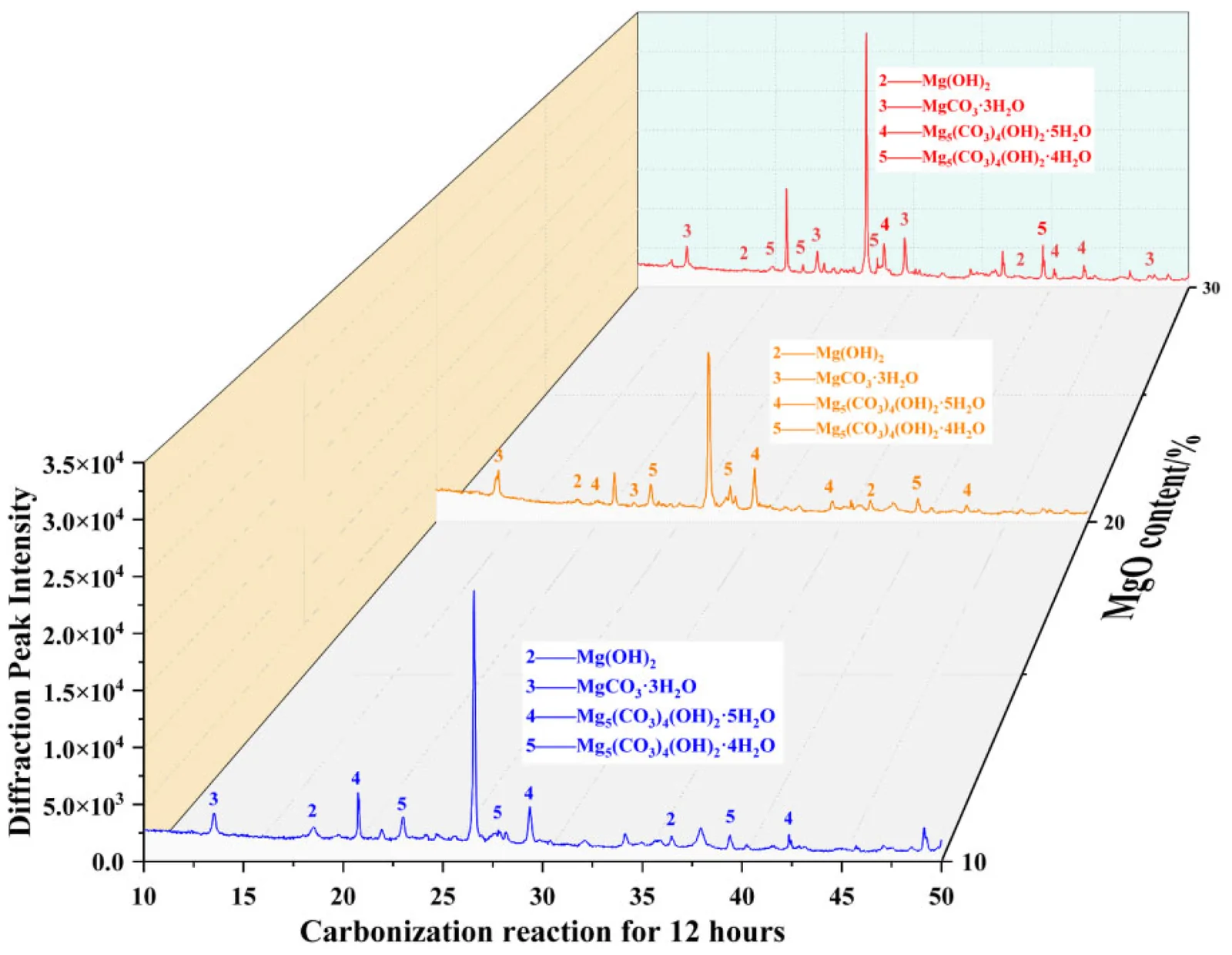
XRD analysis results of samples with different MgO contents at 12 h.

Quantitative X-ray diffraction (XRD) analysis revealed that after 12 h of carbonation (Figure 40), the specimen with 30% MgO content exhibited 13 distinct characteristic diffraction peaks—significantly more than the 20% MgO specimen (11 peaks) and the 10% MgO specimen (10 peaks). Notably, several high-intensity diffraction peaks detected in the 30% MgO specimen closely matched the crystallographic planes of nesquehonite (MgCO_3_·3H_2_O).

Micromechanical analysis indicated that the formation of a three-dimensional interlocking skeletal structure composed of nesquehonite was a primary contributor to the significantly enhanced unconfined compressive strength observed in the 30% MgO specimen, which reached a value approximately 4.9 times higher than that of the 10% MgO specimen. The strengthening effect can be attributed to the combined action of two observable microstructural mechanisms: (1) the physical filling and bridging of interparticle voids and contact regions by the precipitated nesquehonite framework, which densifies the soil matrix and improves load transfer efficiency; and (2) the inherent structural integrity of the highly crystalline carbonate phase, which effectively impedes the initiation and propagation of microcracks within the stabilized material.

These findings elucidate, from a crystallographic perspective, the intrinsic mechanism by which MgO content optimizes mechanical properties through regulation of the phase composition of carbonation products.

**Figure 41:**
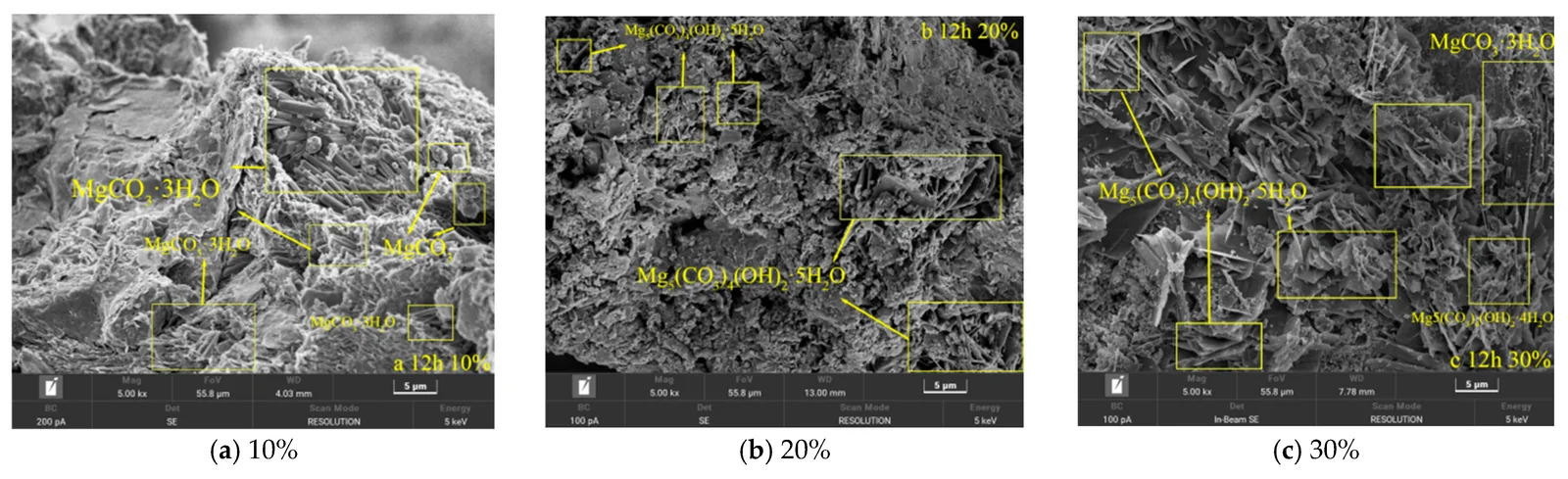
SEM Scanning Results of Different MgO Samples after Carbonization for 12 h.

Scanning electron microscopy (SEM) characterization (Figure 41) revealed significant differences in the micromorphology of specimens with varying MgO contents after 12 h of carbonation. In the low-content system (10% MgO), nesquehonite (MgCO_3_·3H_2_O) appeared as densely distributed plate-like crystals [33] accompanied by a small amount of isolated magnesite (MgCO_3_). In the 20% MgO specimen, dypingite (Mg_5_(CO_3_)_4_(OH)_2_·5H_2_O) formed continuous layered structures [34], exhibiting significantly higher coverage than sporadically distributed hydromagnesite (Mg_5_(CO_3_)_4_(OH)_2_·4H_2_O).

The microstructure of the high-content specimen (30% MgO) exhibited multiphase synergistic evolution: surface reconstruction occurred in nesquehonite crystals, with approximately 20% of the area undergoing transformation into dypingite via a dissolution–recrystallization mechanism, followed by further transition to hydromagnesite [35].

These gradient phase transformation characteristics indicate that MgO content governs the competitive nucleation and growth pathways of magnesium-based minerals by modulating local supersaturation and ion migration rates [36], thereby influencing the macro- and micromechanical response mechanisms of the material.

**Figure 42:**
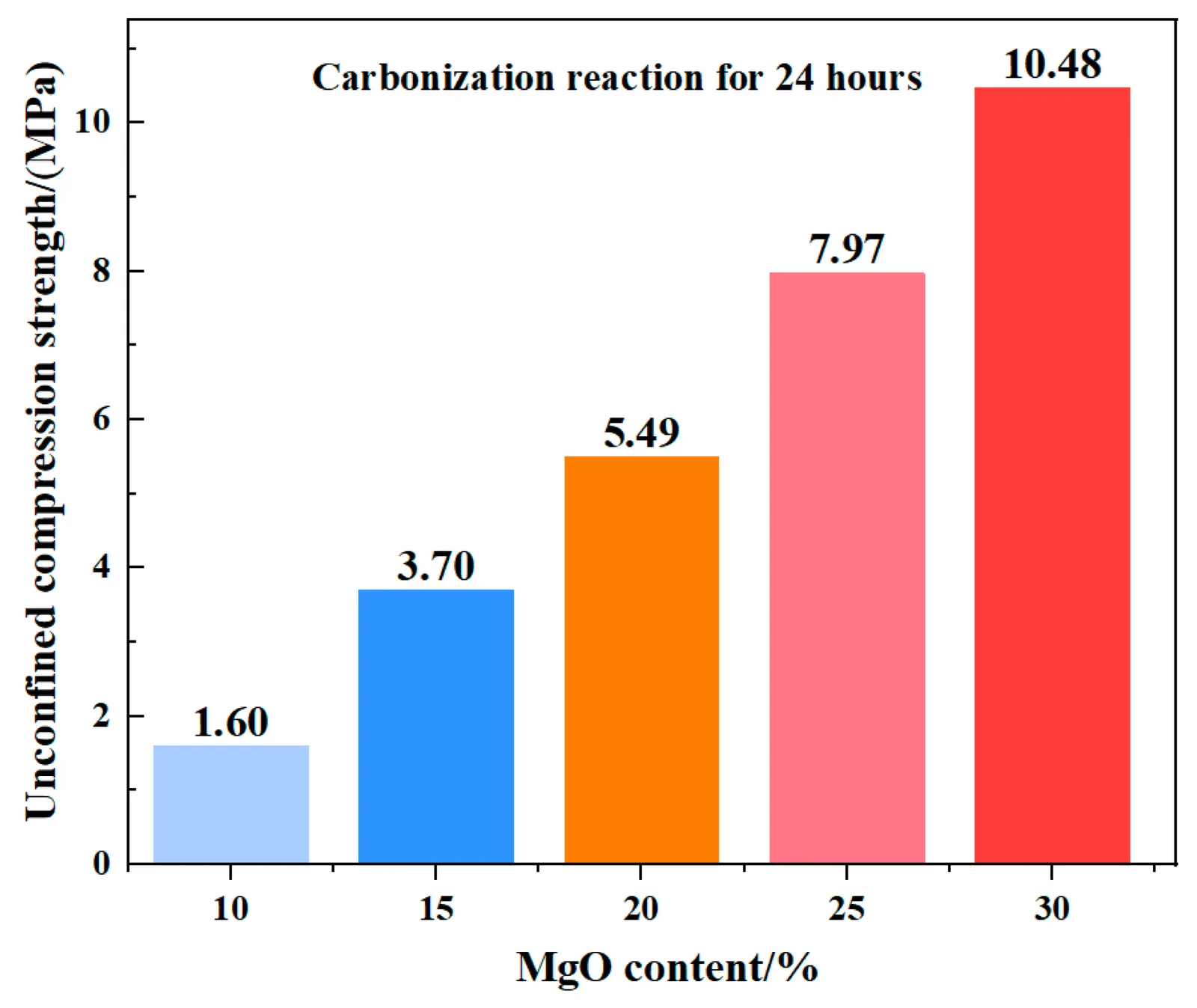
Unconfined compressive strength of samples with different MgO contents at 24 h.

Figure 42 presents the unconfined compressive strength (UCS) of specimens after 24 h of carbonation, while the corresponding 12 h UCS values are shown in Figure 36. A direct comparison of the strength development between these two time points reveals a distinct divergence in behavior as a function of MgO content. Based on the mean values of three replicate tests (Table 4), the UCS increased from 1.532 MPa to 1.597 MPa for the 10% MgO specimen, and from 3.545 MPa to 3.701 MPa for the 15% MgO specimen, corresponding to modest relative gains of 4.2% and 4.4%, respectively. In contrast, the specimens with higher MgO dosages exhibited substantially greater strength development: the 20% MgO specimen increased from 4.684 MPa to 5.494 MPa (+17.3%), the 25% MgO specimen from 6.255 MPa to 7.972 MPa (+27.4%), and the 30% MgO specimen from 7.485 MPa to 10.478 MPa (+40.0%).

This contrasting behavior is consistent with the carbonation progress analysis presented in Section 3.1. The low-MgO systems (10% and 15%) approached carbonation completion within approximately 12 h, as evidenced by the sharp decline in their reaction rates beyond this point. Consequently, only minor additional strength was gained during the subsequent 12 h period. In the high-MgO systems (20–30%), however, the carbonation reaction remained active well beyond 12 h, driven by the continued availability of unreacted MgO and the progressive phase transformation of magnesium carbonates (nesquehonite → dypingite → hydromagnesite). The sustained precipitation and structural reorganization of these carbonate phases, as documented in the XRD and SEM analyses, account for the pronounced late-stage strength enhancement.

Thus, the macro-micro correlation established across the 12 h and 24 h intervals demonstrates that higher MgO contents not only elevate the absolute strength level but also prolong the duration over which significant strength gain occurs, owing to the extended evolution of the carbonate-based cementitious framework.

**Figure 43:**
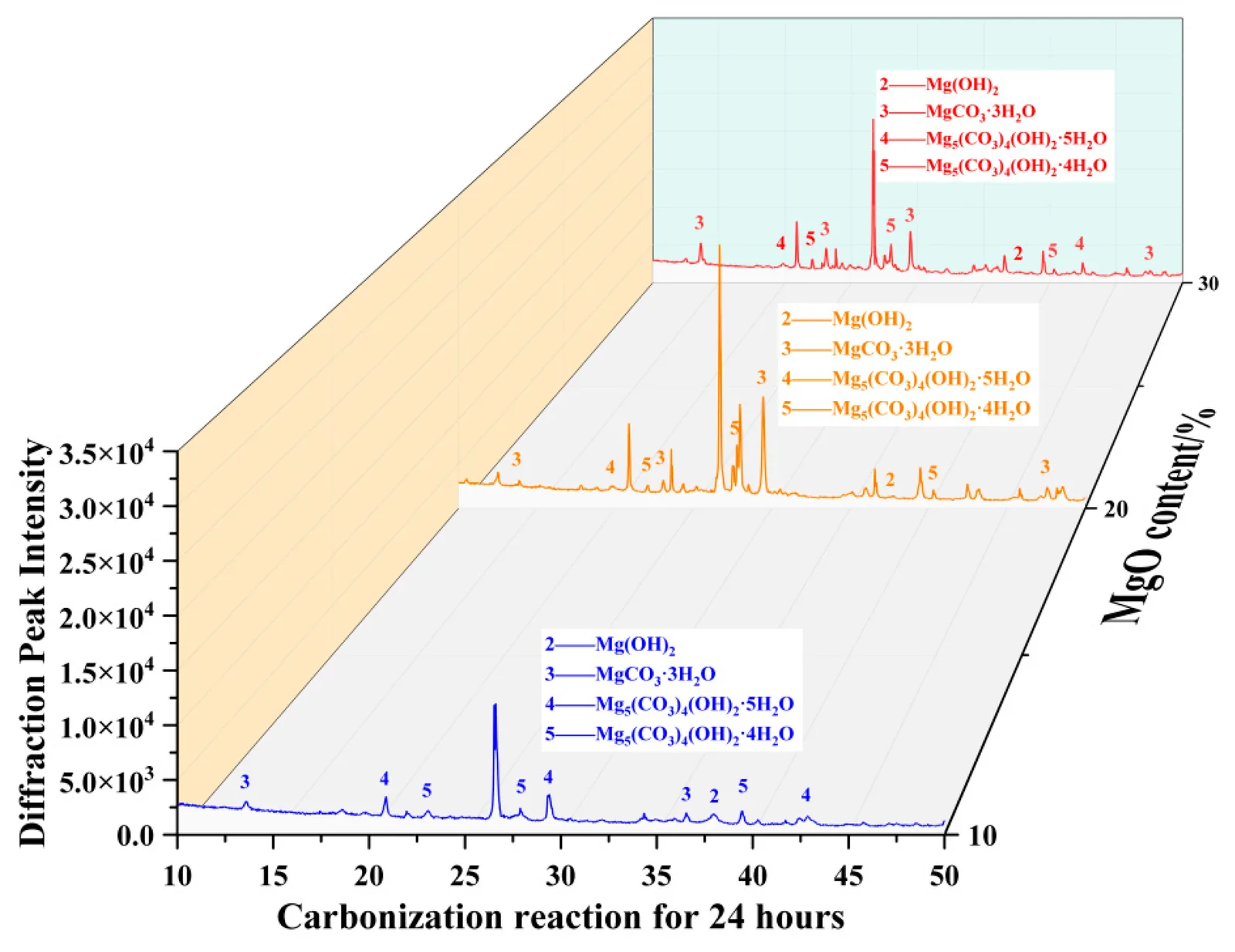
XRD analysis results of samples with different MgO contents at 24 h.

X-ray diffraction (XRD) analysis of specimens after 24 h of carbonation (Figure 43) revealed distinct differences in phase assemblage as a function of MgO content. The diffraction patterns of both the 10% and 20% MgO specimens displayed approximately ten discernible reflections, yet the relative intensity distributions differed markedly. The 10% MgO specimen exhibited generally low peak intensities without a clearly dominant phase, whereas the 20% MgO specimen showed a prominent high-intensity peak corresponding to nesquehonite (MgCO_3_·3H_2_O), indicating that this phase was the predominant crystalline product.

In the high-content specimen (30% MgO), the diffraction pattern exhibited a multiphase character. In addition to reflections attributable to nesquehonite, discernible peaks associated with dypingite (Mg_5_(CO_3_)_4_(OH)_2_·5H_2_O) and hydromagnesite (Mg_5_(CO_3_)_4_(OH)_2_·4H_2_O) were also detected. The sequential appearance of these phases suggests a progressive transformation of magnesium carbonate minerals with increasing MgO availability and extended carbonation. This graded phase evolution is qualitatively consistent with the sustained strength development observed in the high-MgO specimens and supports the interpretation that MgO content exerts a controlling influence on the long-term evolution of the carbonation products [37].

**Figure 44:**
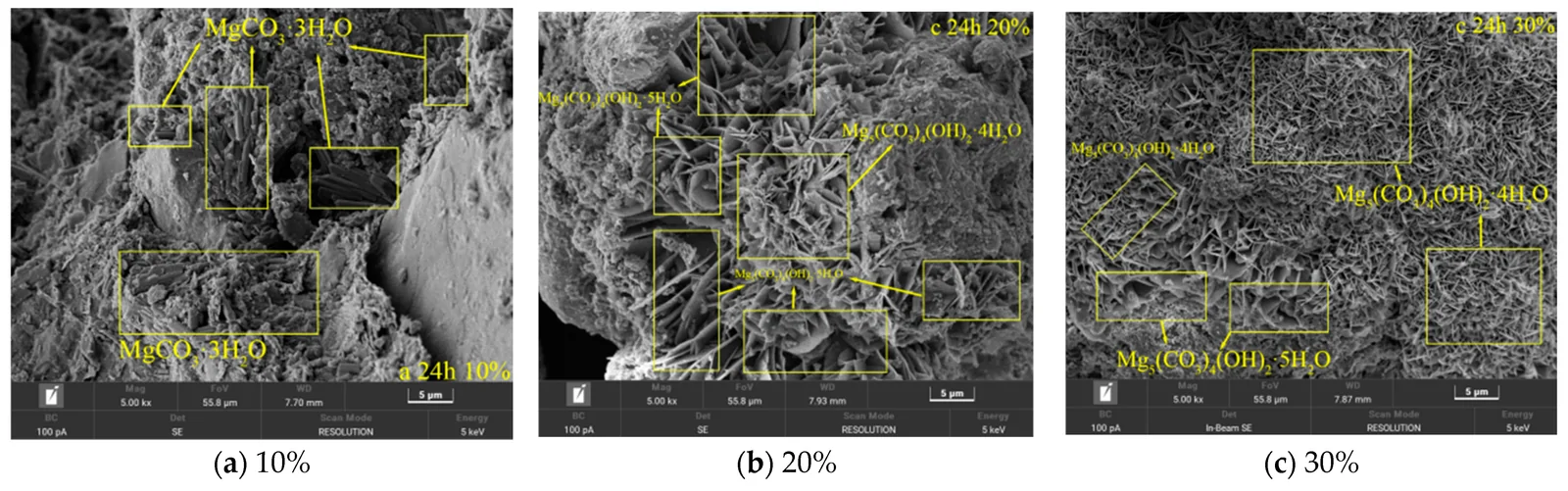
SEM Scanning Results of Different MgO Samples after Carbonization for 24 h.

SEM (Scanning Electron Microscopy) characterization reveals (Figure 44) that after 24 h of carbonation treatment, the distribution of magnesium carbonate mineral phases in samples with different MgO contents exhibits significant differences. In the system with a low content of 10%, nesquehonite (MgCO_3_·3H_2_O) remains the dominant phase, occurring as dense plate-like crystals, which corresponds to the lower unconfined compressive strength values. In the sample with 20% MgO content, hydromagnesite (Mg_5_(CO_3_)_4_(OH)_2_·4H_2_O) forms a continuous network structure through an interface reaction-dominated nucleation mechanism, accompanied by discrete distribution of a small amount of dypingite (Mg_5_(CO_3_)_4_ (OH)_2_·5H_2_O).

The mineral phase composition of the high-content sample (30% MgO) shows significant evolutionary characteristics: approximately 86.5% of Mg^2+^ has been converted to hydromagnesite (Mg_5_(CO_3_)_4_(OH)_2_·4H_2_O), which forms a three-dimensional interwoven framework through the aggregation of flake-like flower-shaped crystals [38]. Residual dypingite is primarily distributed along crystalline boundary regions.

The aforementioned phase distribution characteristics are highly consistent with the mechanical property testing results, confirming that the spatial topological structure of magnesium carbonate minerals (e.g., continuous framework formation, grain boundary strengthening) is a key microscopic mechanism governing the unconfined compressive strength.

**Figure 45:**
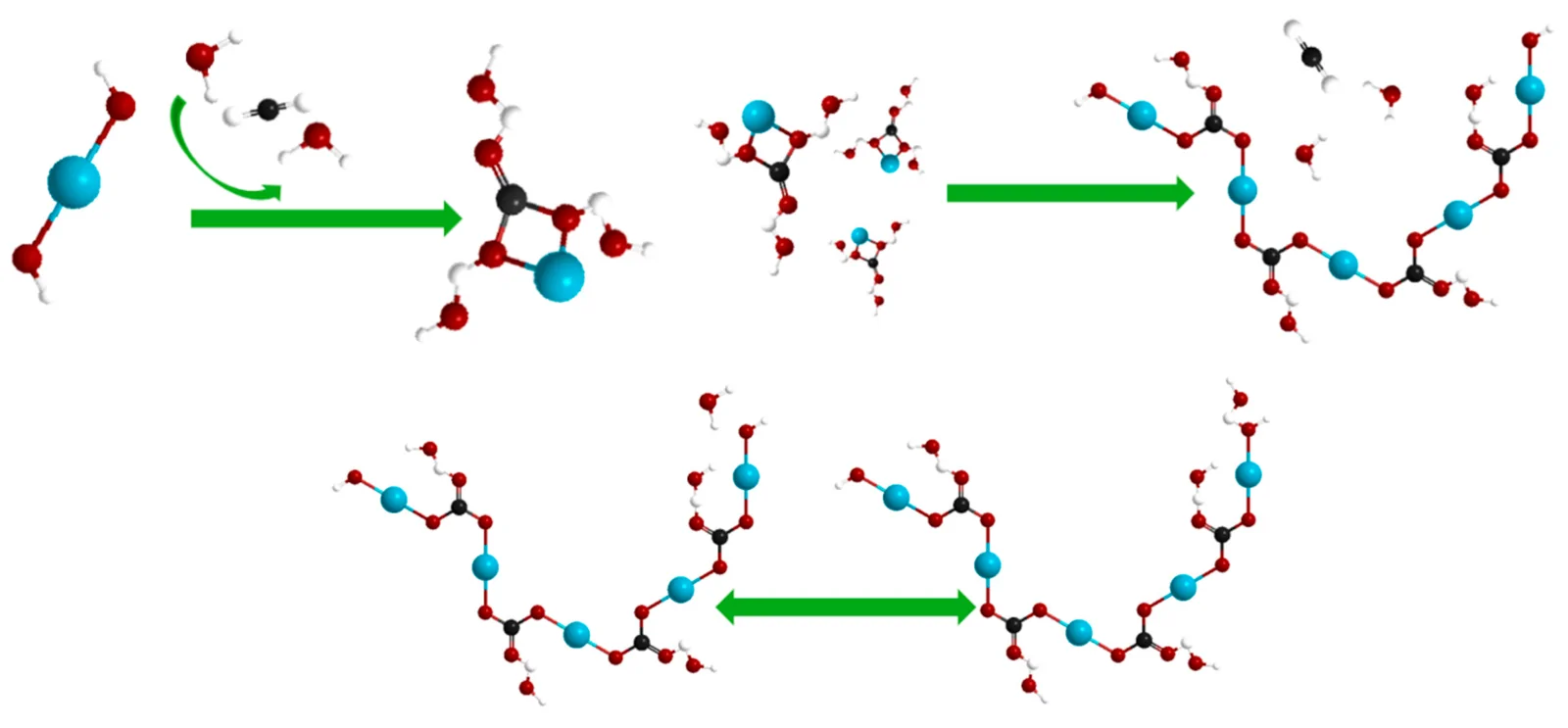
The ball-and-stick model of nesquehonite → dypingite → hydromagnesite. (Note: Unidirectional arrows denote reaction progression, whereas bidirectional arrows indicate reversible reactions. In the ball-and-stick model, the blue spheres represent Mg, the red spheres represent O, the white spheres represent H, and the black spheres represent C.).

This study demonstrates that MgO content exerts a significant influence on the evolution of magnesium-bearing minerals during carbonation, affecting both their spatial distribution and the apparent sequence of phase development [39]. Based on SEM morphological observations and XRD phase identification, the microstructural evidence suggests that systems with higher MgO content (≥20%) promote the formation of aggregated Mg(OH)_2_ clusters, a phenomenon qualitatively consistent with conditions of elevated local supersaturation. This initial aggregation appears to facilitate a progressive transformation of magnesium carbonates, with the mineral assemblage evolving from nesquehonite to dypingite and ultimately to hydromagnesite as carbonation proceeds (Figure 45) [40]. While the precise kinetic pathways and the quantitative contributions of each phase to strength development remain to be fully elucidated, the observed microstructural progression provides a plausible framework for interpreting the macroscopic mechanical enhancement. Specifically, the combined effects of cementitious bonding by Mg(OH)_2_ clusters, pore infilling by nesquehonite frameworks, and the development of an interlocking hydromagnesite skeletal network are proposed as the primary microstructural mechanisms underlying the superior performance of the high-MgO systems.

Correlative XRD and SEM analyses reveal that the aforementioned microstructural evolution exhibits a significant positive correlation with unconfined compressive strength, confirming the intrinsic relationship between MgO content and the synergistic optimization of macro-micro properties through regulation of mineral phase composition and spatial topological characteristics. These findings provide a theoretical basis for component design and performance prediction of carbonated solidification materials.

### 3.7. Analysis of Carbonation Products with Different MgO Contents Based on COMSOL Simulation

The experimental results presented in the preceding sections demonstrate that MgO content governs the sequence and spatial extent of magnesium carbonate phase formation. However, the XRD and SEM analyses provide only localized, post-mortem snapshots of the mineralogy and microstructure. To complement these observations and to gain insight into the spatiotemporal evolution of the carbonation products within the specimen cross-section, a series of reactive transport simulations were performed using COMSOL Multiphysics 6.3. These simulations aim to illustrate how the initial MgO concentration influences the predicted distribution of key phases—specifically magnesite and hydromagnesite—under the diffusion-controlled carbonation conditions employed in the experiments.

**Figure 46:**
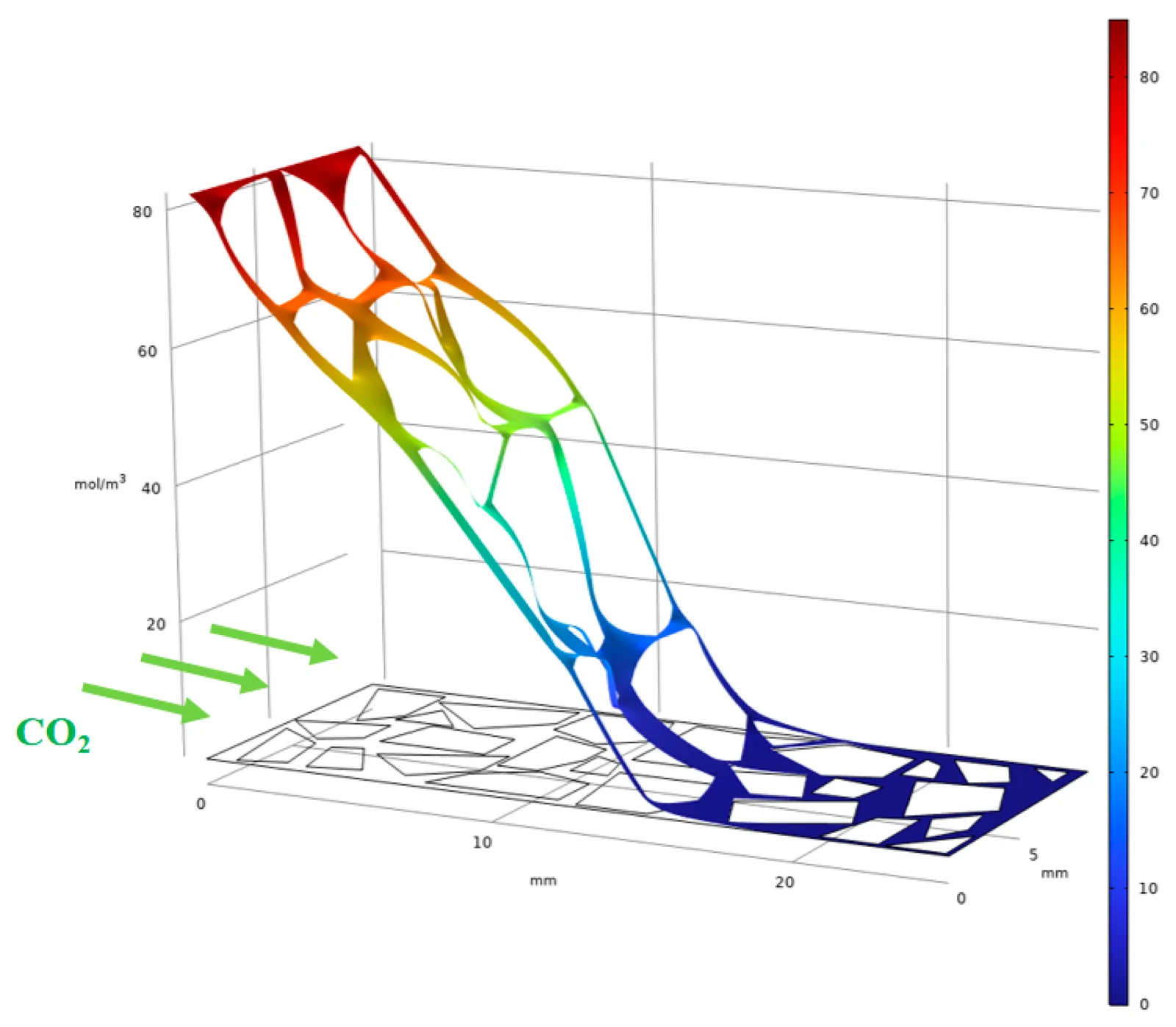
Carbonation schematic diagram. (Note: The image shows color gradient changes from left to right. Red areas represent high carbon dioxide concentrations, while blue areas indicate low carbon dioxide concentrations, visually demonstrating the overall migration path of carbon dioxide).

Based on a simplified real sample, a 2D axisymmetric model was established using the cross-section of a typical carbonated loess specimen. The model measures 25 mm in length and 10 mm in width (Figure 46). The white region represents the loess skeleton composed of solid particles, while the remaining space is defined as a porous reaction zone. This zone is initially filled with water, air, and uniformly dispersed magnesium hydroxide (Mg(OH)_2_), serving as the primary site for carbonation. Carbon dioxide (CO_2_) is introduced from the left boundary at a constant concentration, simulating its diffusion and dissolution within the soil pores.

The molar concentrations reported in Table 5 represent the effective reactive MgO concentration assumed in the COMSOL simulations. These values were derived from the pre-carbonation specimen composition, accounting for the fact that only a fraction of the total added MgO is fully hydrated and available for subsequent carbonation. For each MgO dosage, the total mass of MgO (mtotal) was first calculated from the mix design (MgO content relative to dry soil mass, 23% water content) and the initial specimen volume (V_0_ = 196.35 cm^3^). Based on the hydration behavior observed in the experiments and typical reactivity of the MgO powder used, an activation factor of 0.80 was applied to estimate the reactive portion. The active MgO mass was then converted to moles (M_MgO_ = 40.304 g/mol) and divided by V_0_ to obtain the concentration in mol/m^3^. The resulting values—2617.12, 4936.25, and 7318.23 mol/m^3^—correspond to approximately 80% of the total MgO initially present.

Chemical Reactions: The simulation couples the following three primary homogeneous and heterogeneous chemical reactions:

(1) Main carbonation: Mg(OH)_2_ + CO_2_ = MgCO_3_ + H_2_O
(2) Basic magnesium carbonate formation: 5Mg(OH)_2_ + 4CO_2_ = Mg_5_(CO_3_)_4_(OH)_2_

It should be noted that these two reactions represent a simplified stoichiometric framework for the carbonation process. The actual carbonation products identified in the experiments include hydrated phases such as nesquehonite (MgCO_3_·3H_2_O), dypingite (Mg_5_(CO_3_)_4_(OH)_2_·4H_2_O), and hydromagnesite (Mg_5_(CO_3_)_4_(OH)_2_·5H_2_O), which differ primarily in their degree of hydration. For computational efficiency and to focus on the overall spatial progression of carbonation, only the two representative global reactions shown above were implemented in the COMSOL model. The simulated “magnesite” and “hydromagnesite” (basic magnesium carbonate) phases are therefore best interpreted as qualitative indicators of advanced carbonation zones, rather than exact quantitative predictors of specific hydrated phase distributions.

A total of six comparative simulation cases were established to systematically investigate the influence of two key variables: MgO content (10%, 20%, 30%) and carbonation duration (4 h, 12 h, 20 h). All simulations employed identical boundary and initial environmental conditions to ensure comparability of results, aiming to elucidate the differences in the carbonation process and product formation patterns of MgO at varying incorporation levels [41].

**Figure 47:**
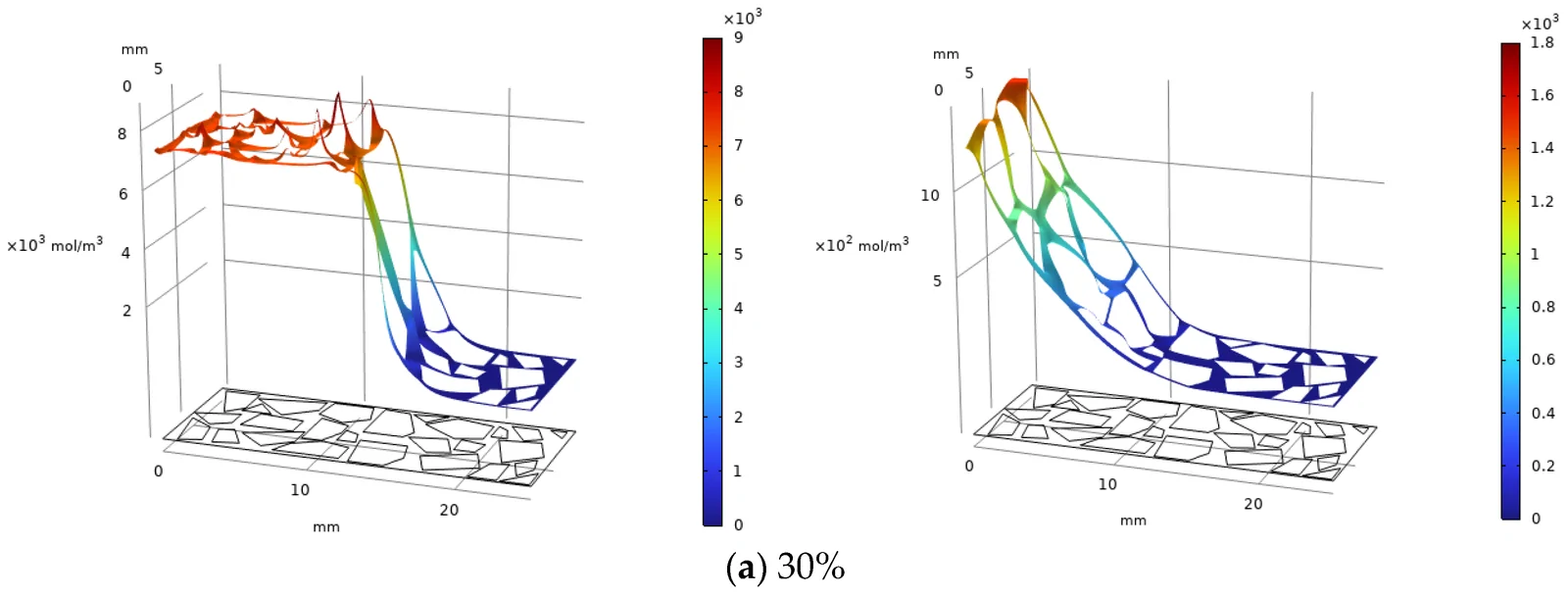

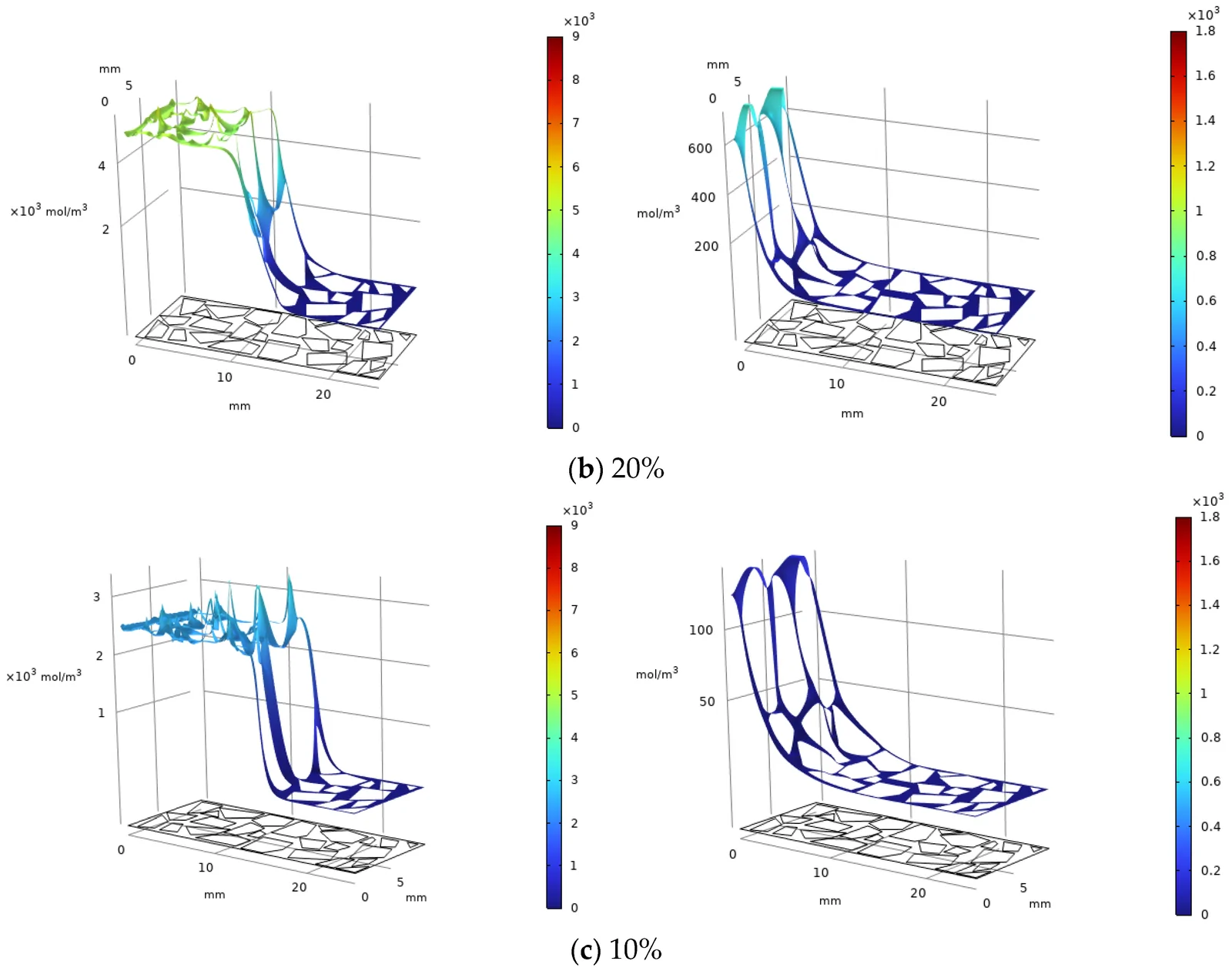
Magnesite (**L**) & Hydromagnesite (**R**)—20 h carbonation, varying MgO contents.

Figure 47 illustrates the simulated spatial distribution of carbonation products after 20 h for specimens with initial MgO contents of 10%, 20%, and 30%. The simulation results show a clear trend: as the MgO content decreases, the predicted peak concentration of magnesite progressively diminishes, and the depth of the carbonated zone becomes correspondingly shallower. This qualitative trend is consistent with the experimental observation that higher MgO dosages sustain more extensive and prolonged carbonation reactions.

The formation of hydromagnesite likewise diminishes with decreasing MgO content. The peak concentration in the 30% specimen is about twice that of the 20% specimen, with a notably greater carbonation depth, while the peak concentration region in the 20% specimen is roughly five times that in the 10% specimen. MgO content directly governs the yield and depth of carbonation products. Higher content significantly enhances carbonate formation efficiency and CO_2_ sequestration, proving crucial for optimized carbonation reinforcement [42].

**Figure 48:**
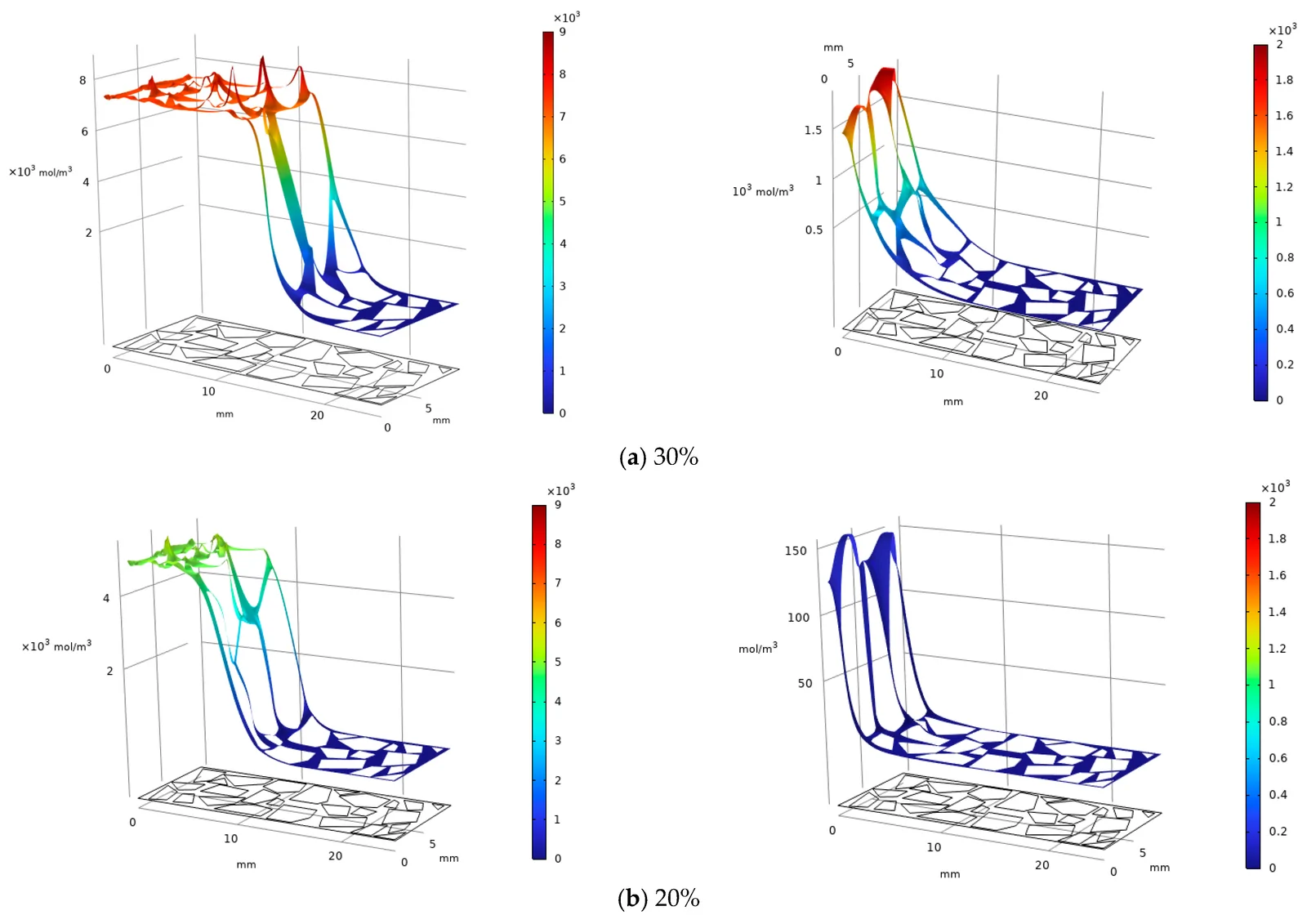

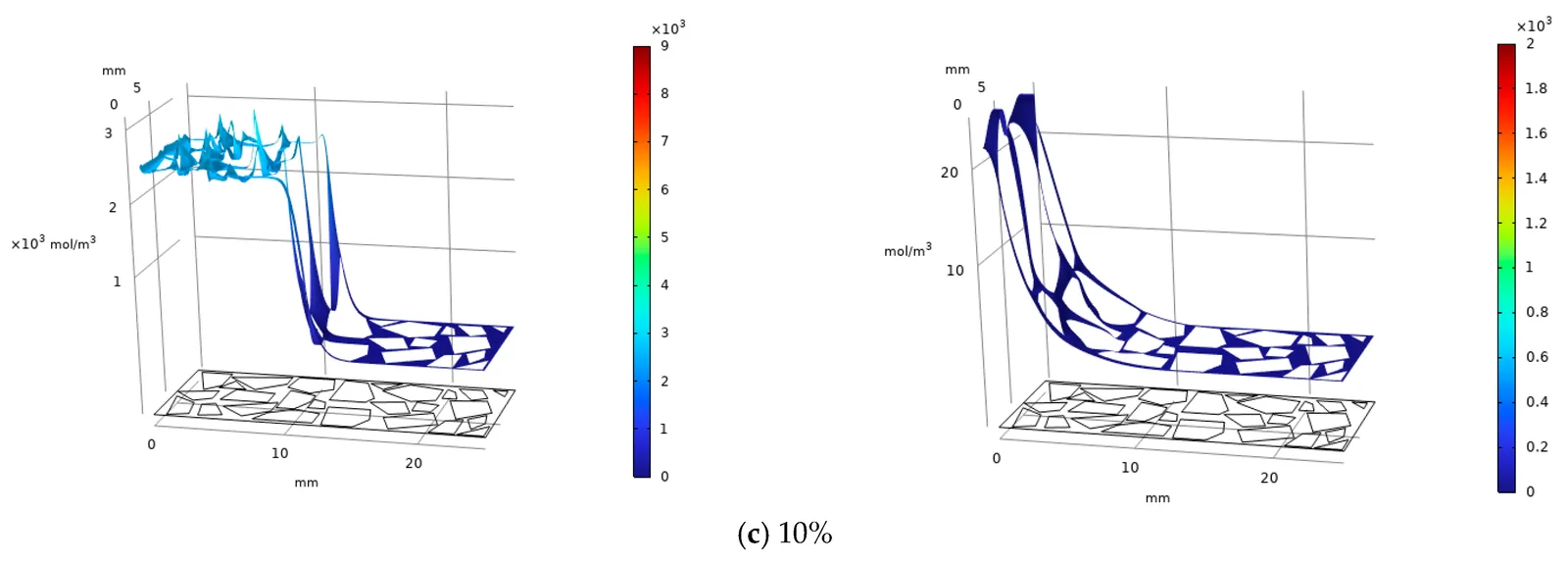
Magnesite (**L**) & Hydromagnesite (**R**)—12 h carbonation, varying MgO contents.

Figure 48 below illustrates the formation of carbonation products after 12 h for specimens with different MgO contents (10%, 20%, and 30%). The yield of magnesite shows a trend similar to that after 20 h of carbonation, though slightly reduced across all contents. In contrast, the formation of hydromagnesite decreases significantly compared to the 20 h results, with peak values for the 10% and 20% specimens dropping by approximately 80% (about 1/5 of the original). After 12 h, the peak hydromagnesite concentration in the 30% specimen is 13 times that of the 20% specimen, while the peak in the 20% specimen is about 120 mol/m^3^ higher than that in the 10% specimen. Under 12 h carbonation, hydromagnesite formation is more time-sensitive, and higher MgO content substantially preserves its production advantage.

**Figure 49:**
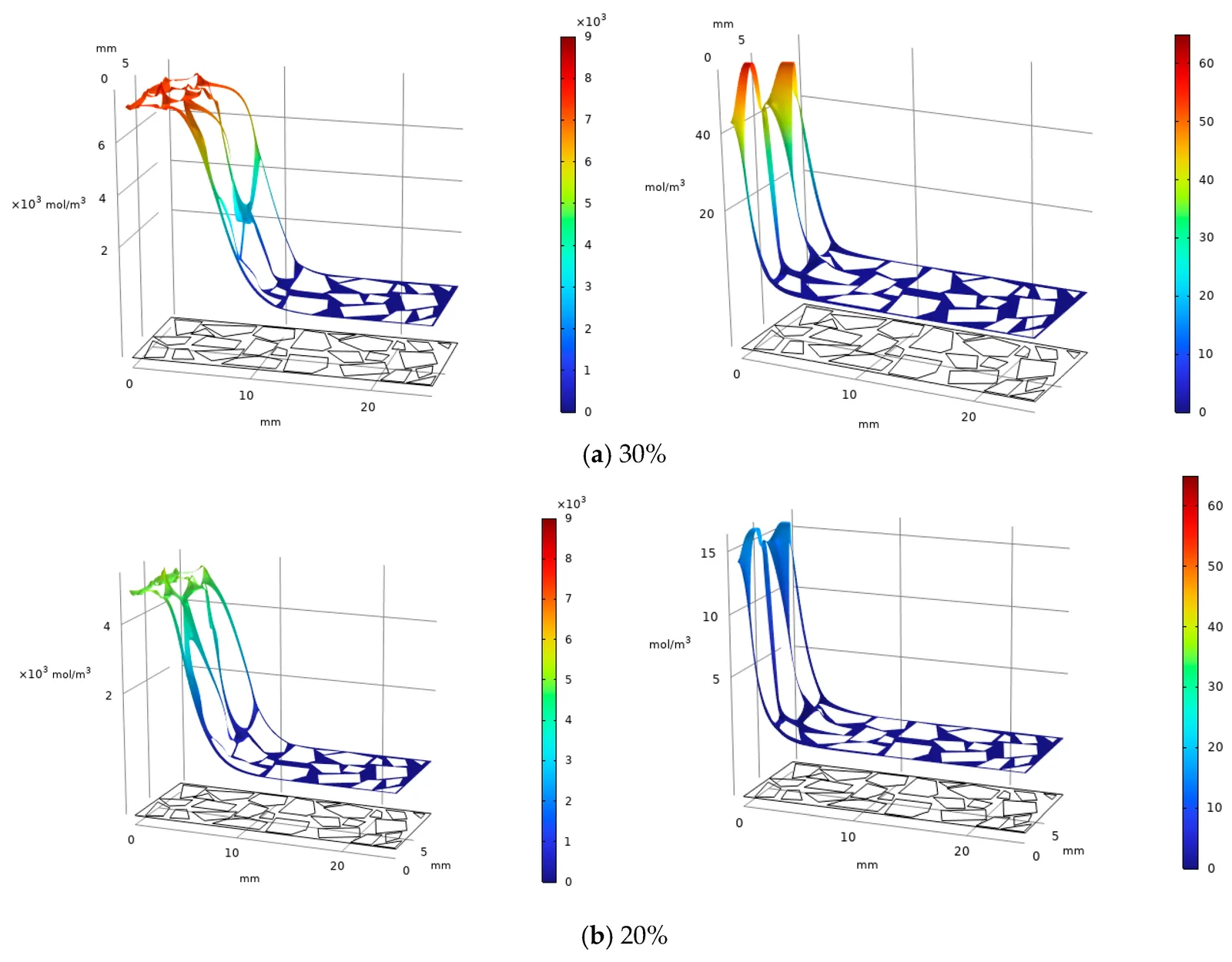

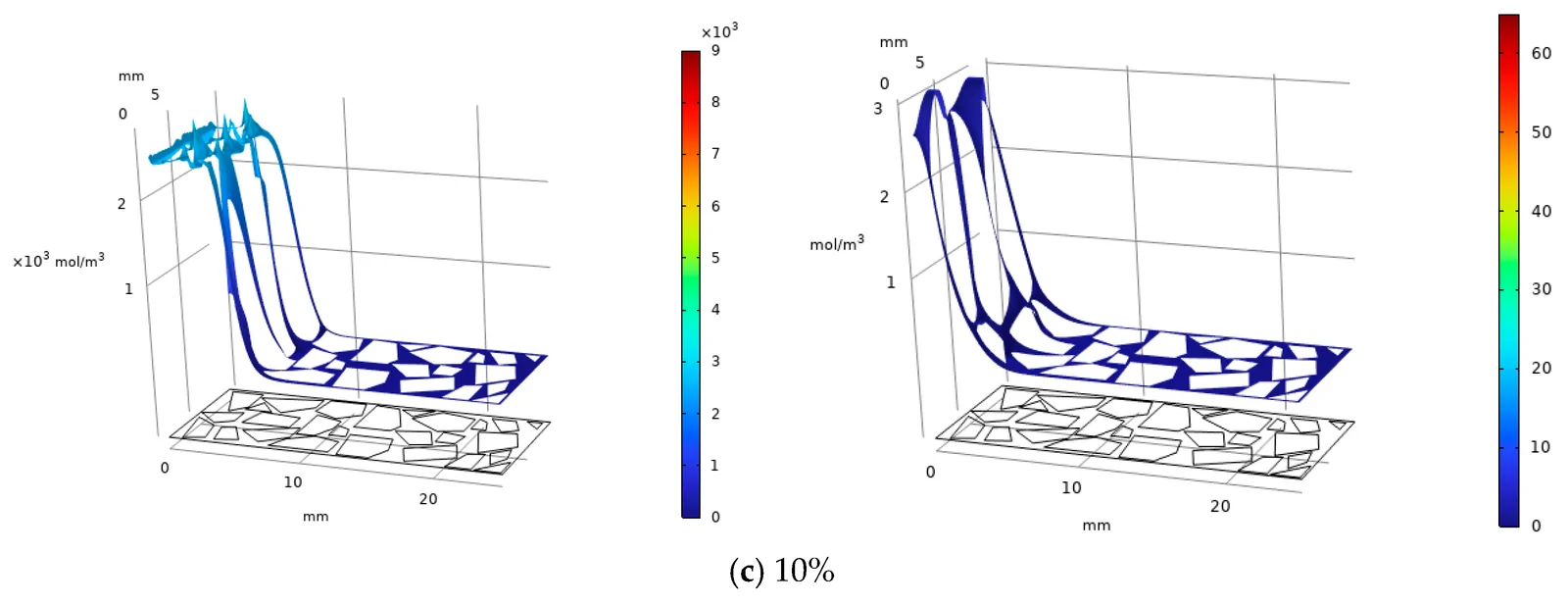
Magnesite (**L**) & Hydromagnesite (**R**)—4 h carbonation, varying MgO contents.

Figure 49 below illustrates the formation of carbonation products after 4 h for specimens with different MgO contents (10%, 20%, and 30%). The peak regions of magnesite show little change compared to those at 12 h, but the carbonation depth in all specimens decreases significantly relative to the 12 h and 20 h results. The formation of hydromagnesite drops sharply, with the 30%, 20%, and 10% specimens all exhibiting orders-of-magnitude reduction compared to the 12 h outcomes. Nevertheless, the 30% specimen still maintains the highest peak concentration of hydromagnesite at about 60 mol/m^3^, while the 20% and 10% specimens remain below 16 mol/m^3^ and 3 mol/m^3^, respectively. Short-term carbonation significantly suppresses product formation, especially for hydromagnesite, yet higher MgO content still preserves a relative production advantage.

### 3.8. Crack Initiation and Propagation Mechanisms

This study examines the qualitative influence of MgO content (10–30%) on the surface cracking behavior of carbonated specimens through visual inspection and comparative macro-micro analysis [43].

**Figure 50:**
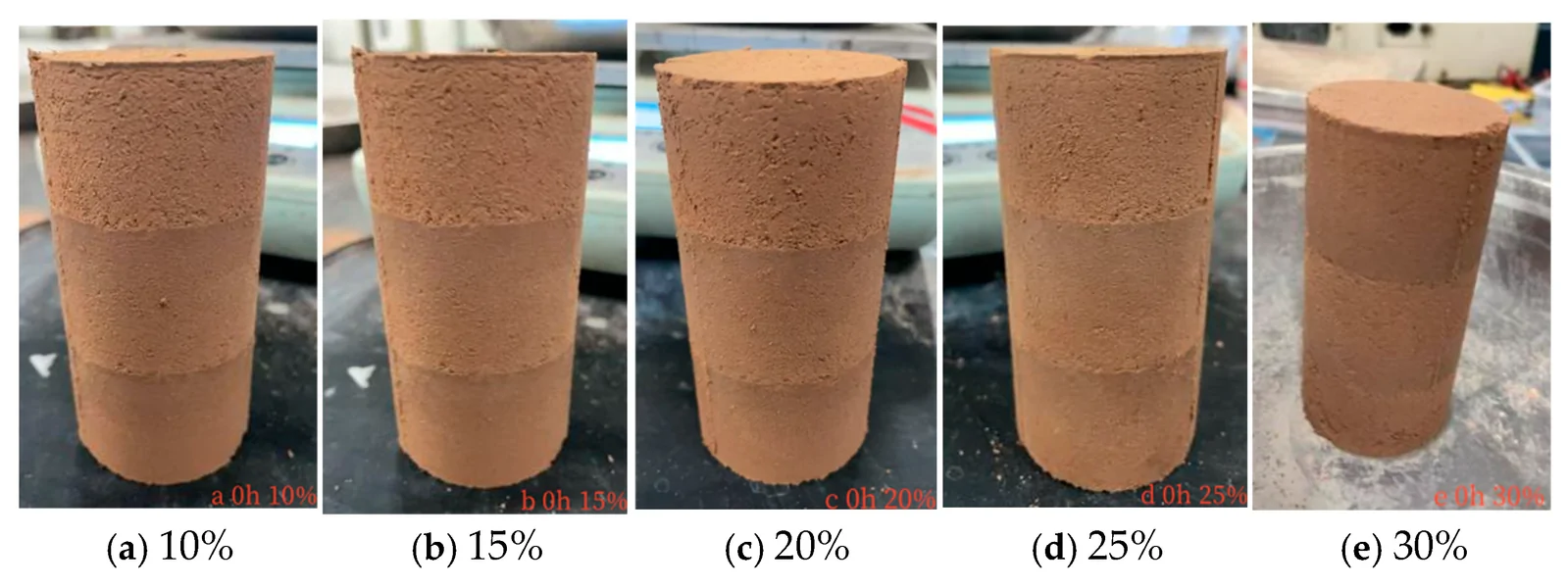
Apparent Diagram of the Sample at the Carbonization Duration of 0 h.

At the initial stage (0 h, Figure 50), visual examination revealed that specimens with higher MgO content exhibited noticeably smoother surfaces than those with lower content, with the contrast most apparent between the 10% and 30% MgO samples. No visible cracks were observed on any specimen at this stage.

**Figure 51:**
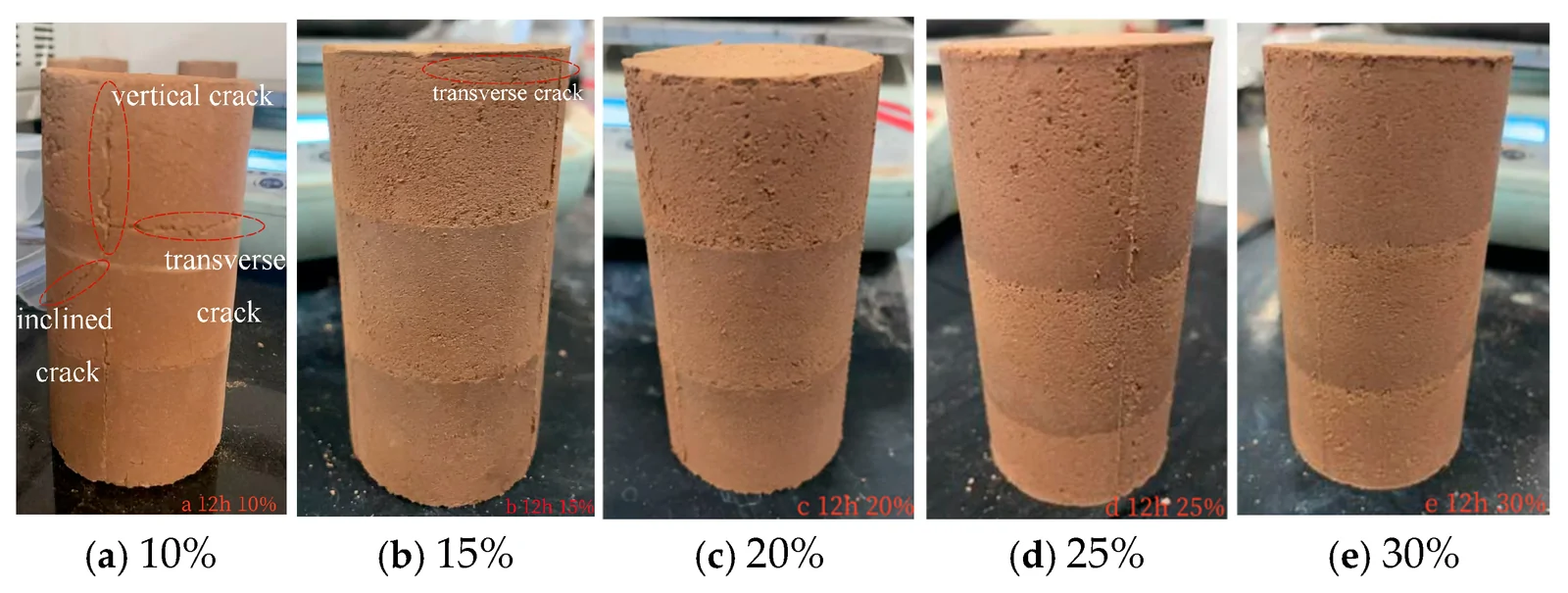
Apparent Diagram of the Sample at the Carbonization Duration of 12 h.

After 12 h of carbonation (Figure 51), fine surface cracks became visible on the 10% and 15% MgO specimens, whereas specimens with 20–30% MgO remained largely crack-free upon visual inspection. This qualitative difference suggests that the more abundant precipitation of magnesium carbonate minerals in high-MgO specimens forms a denser surface layer that suppresses the initiation of visible cracks.

**Figure 52:**
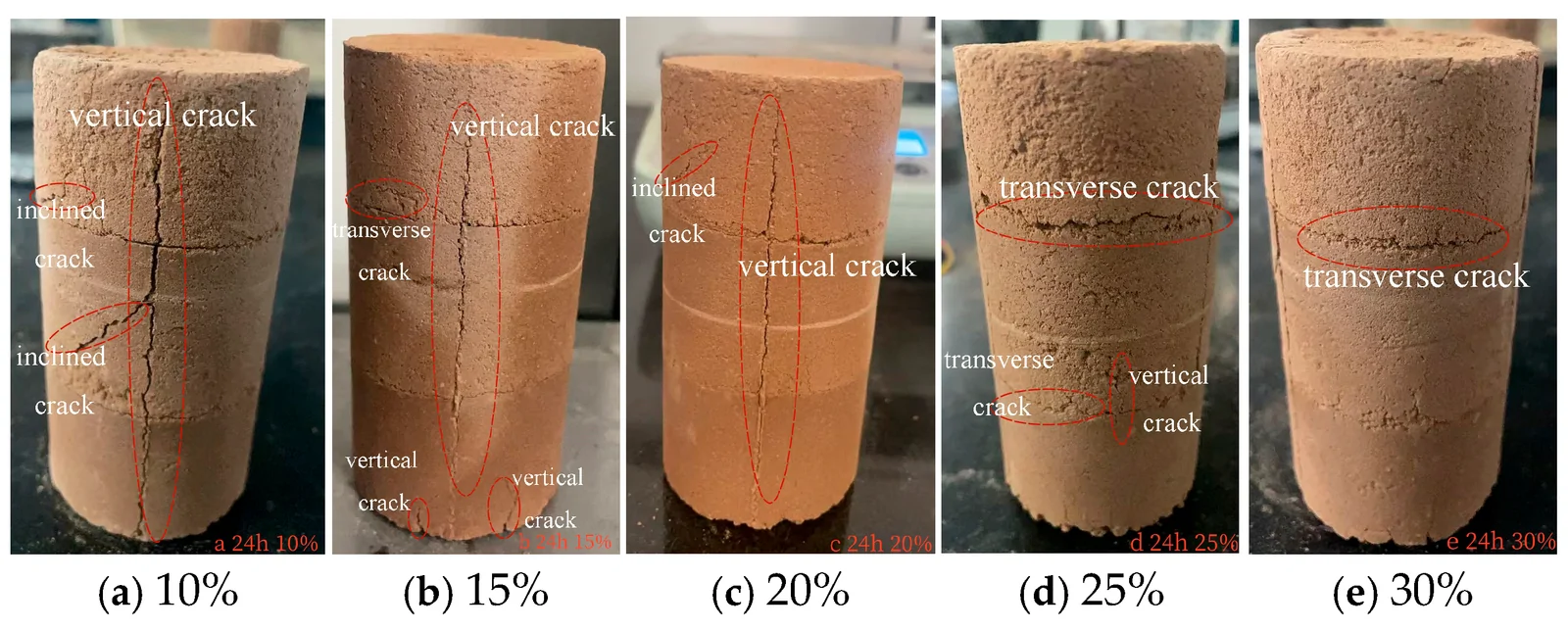
Apparent Diagram of the Sample at the Carbonization Duration of 24 h.

After 24 h of carbonation (Figure 52), the trend persisted: specimens with lower MgO content (10–15%) displayed more numerous and deeper visible cracks, while those with higher content (25–30%) exhibited markedly fewer and shallower surface defects. These visual observations are consistent with the microstructural evidence presented in Section 3.6, where high MgO contents were associated with a denser, more interlocked carbonate framework.

Specifically, in samples with low MgO content (10%), the dominant mineral phase was nesquehonite, reflecting a lower degree of carbonation and a relatively porous microstructure, which led to numerous deep cracks. When MgO content increased to 20%, the main mineral phase shifted to magnesite, distributed extensively throughout the material, indicating more complete carbonation and a denser microstructure that significantly reduced crack formation. Further increasing MgO content to 30% promoted the formation of more stable magnesium salt minerals, resulting in a highly densified microstructure, markedly improved mechanical strength, and further suppression of crack generation.

In summary, increased MgO content promotes the formation of magnesite and hydromagnesite at the microscopic level, resulting in a more stable material structure. Macroscopically, this manifests as reduced crack density and decreased crack depth. This direct correlation between microscopic mineral phase evolution and macroscopic mechanical properties further confirms the critical role of MgO content in enhancing carbonation efficiency and material durability.

## 4. Conclusions

This study elucidates the regulatory role of MgO content in the carbonation curing of collapsible loess, establishing quantitative linkages among MgO dosage, phase evolution, and mechanical performance. The key findings are summarized as follows.

Higher MgO content (20–30%) fundamentally alters the carbonation mechanism by sustaining elevated local supersaturation, which drives a sequential mineral transformation—nesquehonite → dypingite → hydromagnesite. This gradient phase evolution is absent in low-MgO systems and constitutes the microstructural basis for the observed mechanical enhancement. At 24 h of carbonation, the 30% MgO specimen achieved a UCS of 10.48 MPa—approximately 6.6 times that of the 10% MgO control and 2.8 times that of the 15% MgO specimen. Notably, high-MgO specimens exhibited substantial late-stage strength gains, whereas low-MgO counterparts showed marginal improvement due to premature reaction saturation.

The strengthening mechanism operates through three synergistic stages: (i) early-age framework formation via Mg(OH)_2_ agglomeration; (ii) intermediate pore-infilling by a three-dimensional nesquehonite network; and (iii) advanced development of an interlocking hydromagnesite skeleton that densifies the matrix and inhibits microcrack propagation. Abaqus simulations accurately reproduced the experimental UCS response, while COMSOL modeling confirmed that higher MgO content significantly enhances both the yield and penetration depth of the critical hydromagnesite phase.

Several limitations warrant acknowledgment. The accelerated carbonation regime (≥50% CO_2_) deviates substantially from natural conditions, potentially altering phase stability and reaction kinetics. Furthermore, the use of homogenized, remolded specimens disregards the inherent macro-porous fabric and vertical jointing of in situ loess. Long-term durability under freeze–thaw and wet–dry cycles also remains uninvestigated. Addressing these gaps through field trials and extended environmental exposure testing is essential for translating this low-carbon stabilization technology into robust engineering practice.

## Figures and Tables

**Table 1:** Physical and index properties of the collapsible loess (Mean ± SD, *n* = 3).

Property	Symbol	Unit	Mean Value	Standard Deviation (±)
Initial Moisture Content	ω	%	3.27	0.15
Specific Gravity	Gs	—	2.724	0.008
Curvature Coefficient	Cc	—	4.082	0.120
Liquid Limit	$\omega_{L}$	%	24.66	0.85
Plastic Limit	$\omega_{P}$	%	19.03	0.60
Plasticity Index	$I_{p}$	%	5.63	0.25
Maximum Dry Density	$\rho_{d,\max}$	g/cm^3^	1.895	0.012
Optimum Moisture Content	$\omega_{\mathrm{opt}}$	%	12.66	0.18
Collapsibility Coefficient	δs	—	0.0648	0.0015

**Table 2:** The basic physical parameters set for the plain soil and different MgO contents.

MgO Content	Density (g/cm^3^)	Diameter (mm)	Height (mm)	Young’s Modulus (MPa)	Poisson’s ratio	Internal Friction Angle (°)	Cohesion (kPa)
soil	1.890	50	100	8.28	0.30	28.61	31.16
10%	1.948	52	104	90	0.29	29.0	476
15%	1.989	52	104	210	0.28	29.5	1096
20%	2.018	51.2	102.5	320	0.28	30.0	1577
25%	2.038	51.2	102.5	480	0.27	30.5	2267
30%	2.058	51	102	660	0.27	31	2997
35%	2.080	51	102	750	0.26	31.5	3251

**Table 3:** Experimental vs. Simulated UCS and Relative Errors for Various MgO Contents.

MgO Content	Experimental UCS (MPa)	Simulated UCS (MPa)	Relative Error
10%	1.568	1.616	+3.06%
15%	3.681	3.742	+1.66%
20%	5.388	5.466	+1.45%
25%	7.939	7.960	+0.26%
30%	10.478	10.670	+1.83%

**Table 4:** Effect of MgO Content on UCS Growth of Specimens at 12 h and 24 h.

MgO Content	12 h UCS (MPa)	24 h UCS (MPa)	Absolute Increase (MPa)	Relative Growth Rate (%)
10%	1.532	1.597	0.065	4.2%
15%	3.545	3.701	0.156	4.4%
20%	4.684	5.494	0.810	17.3%
25%	6.255	7.972	1.717	27.4%
30%	7.485	10.478	2.993	40.0%

**Table 5:** Parameters for Different MgO Contents.

MgO Content	Total MgO Mass (g)	Total MgO Conc. (mol/m^3^)	Active Fraction	Active MgO Conc. (mol/m^3^)
10%	20.71	~3271	0.80	2617.12
20%	39.06	~6170	0.80	4936.25
30%	57.92	~9148	0.80	7318.23

## Data Availability

The original contributions presented in this study are included in the article. Further inquiries can be directed to the corresponding author.

## References

[1] Kondo A., Kurosawa R., Ryu J., Matsuoka M., Takeuchi M. (2021). Investigation on the Mechanisms of Mg(OH)_2_ Dehydration and MgO Hydration by Near-Infrared Spectroscopy. J. Phys. Chem. C.

[2] Shang Z., Du G., Zhang D., Liu S., Guo Q., Xia H., Qian X. (2020). A Review of Carbonated Reactive MgO-stabilized Soil. MATEC Web Conf..

[3] Onyekwena C.C., Xue Q., Li Q., Umeobi H.I., Ghaffar A., Fasihnikoutalab M.H. (2023). Advances in the carbonation of MgO-based binder and CO_2_ utilization in the construction industry. Clean Technol. Environ. Policy.

[4] Miao T., Liu Z., Niu Y. (2002). Unified Catastrophic Model for Collapsible Loess. J. Eng. Mech..

[5] Chen K., Song Y., Chen Y., Lai Y., Kong Z.L. (2026). Strength and leaching characteristics of lead-zinc contaminated red clay stabilized by CO_2_-enhanced active MgO carbonation. J. Environ. Chem. Eng..

[6] Li W., Qin J., Yi Y. (2021). Carbonating MgO for treatment of manganese- and cadmium-contaminated soils. Chemosphere.

[7] Zhang Y., Ong Y.J., Yi Y. (2022). Comparison between CaO- and MgO-activated ground granulated blast-furnace slag (GGBS) for stabilization/solidification of Zn-contaminated clay slurry. Chemosphere.

[8] Ji H., Fan X., Ding F. (2025). Mechanical Characteristics of Soft Clay Solidified by Incorporating Granulated Blast Furnace Slag, Magnesium Oxide, and Building Gypsum. Materials.

[9] Kong X., Zhang Z., Liang Y., Wang X., Liu M. (2023). Experimental study on solidified dredged sediment with MgO and industrial waste residue. Constr. Build. Mater..

[10] Zucha W.J., Bernard E., Kuhn R., Plötze M., Puzrin A. (2025). Effects of MgO-based cementitious binder on smectites. Appl. Clay Sci..

[11] Li Z., Zhao Z., Shi H., Li J., Zhao C., Wang P. (2022). Experimental Study on PVA-MgO Composite Improvement of Sandy Soil. Materials.

[12] Hu C., Shen K., Qin Y., Qian X., Wang F. (2024). Sustainable MgO-calcined clay cementitious material: Reaction mechanism, strength development and performance enhancement via initial CO_2_ stirring. Sustain. Mater. Technol..

[13] Unluer C., Al-Tabbaa A. (2013). Impact of hydrated magnesium carbonate additives on the carbonation of reactive MgO cements. Cem. Concr. Res..

[14] Panesar D.K., Mo L. (2013). Properties of binary and ternary reactive MgO mortar blends subjected to CO_2_ curing. Cem. Concr. Compos..

[15] Vandeperre L.J., Liska M., Al-Tabbaa A. (2008). Microstructures of reactive magnesia cement blends. Cem. Concr. Compos..

[16] Liska M., Al-Tabbaa A. (2008). Performance of magnesia cements in pressed masonry units with natural aggregates: Production parameters optimization. Constr. Build. Mater..

[17] Shi Y.M., Chen F.Z., Zhang Y.H. (2012). Analysis of Collapsible Loess Microstructure at Fractal Theory Based. Appl. Mech. Mater..

[18] Cai J., Dong Y.B. (2011). Micro-Structure Study on Collapsibility Loess with SEM Method. Appl. Mech. Mater..

[19] Wang N. (2018). Analysis of Temperature Variation Characteristics in Horinger County over the Past Three Decades. Mod. Agric. Mach..

[20] Bian H., Wei J. (2024). Bearing Characteristics and Negative Skin Friction Preventive Measures for Highway Bridge Pile Foundations in Collapsible Loess Areas Under Water Immersion. Water.

[21] (2020). Test Methods of Soils for Highway Engineering.

[22] Liao Y.D., Jiang C.H., Feng X.G. (2012). An Empirical Correlation between Unconfined Compression Strength and Curing Time for Cement-Soil. Appl. Mech. Mater..

[23] Handy R., White D. (2008). Evolution of geotechnical soil testing II. Laboratory tests. Int. J. Geotech. Eng..

[24] Li Y., Li B., Lv Y., Zhou J., Qiao H., Chang C., Dong J., Wen J., Wang Q., Zheng W. (2025). Improved CO_2_ retention performance of magnesium oxychloride cement through the synergistic effect of solid waste MgO. Constr. Build. Mater..

[25] Mármol G., García-Lodeiro I. (2025). CO_2_ reactor-curing for early-strength MgO-based cementitious systems. Case Stud. Constr. Mater..

[26] Li B., Min F., Zhou X., Zhang N., Wang X., Yao Z. (2024). Strength characteristics and solidification carbonization mechanism of MgO based shield tunneling centrifugal waste silt. Constr. Build. Mater..

[27] Song Y., Chen K., Yang C., Chen Y., Lai Y., Li H. (2025). Strength Characteristics and Microcementation Effect of Red Clay Improved by Activated MgO Carbonation. Indian Geotech. J..

[28] Avinash B., Kumar R.S. (2022). Bearing Capacity Evaluation of Shallow Foundations on Stabilized Layered Soil using ABAQUS. Stud. Geotech. Mech..

[29] Mo L., Panesar D.K. (2012). Effects of accelerated carbonation on the microstructure of Portland cement pastes containing reactive MgO. Cem. Concr. Res..

[30] Lv Y., Bai L., Ma Y., Zhao L. (2024). Investigation of Crystallization Growth Characteristics of Mg(OH)_2_ Crystals under Unconstrained Conditions. Materials.

[31] Su L., Li H., Yu J.K. (2006). Relationship between activity of magnesium oxide and its microstructure. J. Mater. Metall..

[32] Juan C., Liang Z.H., Zhao W.L. (2016). Synthesis of Porous Basic Magnesium Carbonate Crystallographic Materials with Flower-Like Structure. Key Eng. Mater..

[33] Cheng W., Li Q., Wang Y., Fang L., Cheng F. (2023). Formation and Phase Transformation of MgCO_3_·3H_2_O Whiskers in the Presence of Sodium Dodecyl Sulfate. ACS Omega.

[34] Power I.M., Wilson S., Thom J.M., Dipple G.M., Southam G. (2007). Biologically induced mineralization of dypingite by cyanobacteria from an alkaline wetland near Atlin, British Columbia, Canada. Geochem. Trans..

[35] Sutradhar N., Sinhamahapatra A., Roy B., Bajaj H.C., Mukhopadhyay I., Panda A.B. (2011). Preparation of MgO nano-rods with strong catalytic activity via hydrated basic magnesium carbonates. Mater. Res. Bull..

[36] Zhu Y., German A., Wyrzykowski M., Toropovs N., Winnefeld F., Lura P., Griffa M. (2025). Low-carbon MgO/hydromagnesite binders—Effect of moisture state on the evolution of mechanical properties. Cem. Concr. Res..

[37] Ichiro G.Y., Atsushi K., Satoru O. (2022). Structural variations of amorphous magnesium carbonate during nucleation, crystallization, and decomposition of nesquehonite MgCO_3_·3H_2_O. Phys. Chem. Miner..

[38] Teir S., Kuusik R., Fogelholm C.-J., Zevenhoven R. (2007). Production of magnesium carbonates from serpentinite for long-term storage of CO_2_. Int. J. Miner. Process..

[39] Yang P., Bracco J.N., Camacho Meneses G., Yuan K., Stubbs J.E., Boamah M.D., Brahlek M., Sassi M., Eng P.J., Boebinger M.G. (2025). Carbonation of MgO Single Crystals: Implications for Direct Air Capture of CO_2_. Environ. Sci. Technol..

[40] Kong X., Wang X., Zhang Z., Sun A., Yang L., Zhang F., Xie B., Li Y. (2024). Microscopic Mechanism and Road Performance Analysis of MgO Carbonation–Solidification of Dredged Sediment. Sustainability.

[41] Tong L.Y., Liu Q.F., Gruyaert E., Alderete N.M., Xiong Q.X., De Belie N. (2026). Experimental and numerical study on carbonation of blast-furnace slag concrete considering the microstructural evolution. Cem. Concr. Res..

[42] Choi S.-J., Kim Y.-U., Oh T.-G., Cho B.-S. (2020). Compressive Strength, Chloride Ion Penetrability, and Carbonation Characteristic of Concrete with Mixed Slag Aggregate. Materials.

[43] Hwang K.Y., Kim J.Y., Phan H.Q.H., Ahn J.Y., Kim T.Y., Hwang I. (2018). Effect of CO_2_ concentration on strength development and carbonation of a MgO-based binder for treating fine sediment. Environ. Sci. Pollut. Res..

