# Correction: Yang et al. The Mechanical Properties and Microstructural Evolution Mechanism of Carbonation-Cured Loess with Varying MgO Content. *Materials* 2026, *19*, 2107

**DOI:** 10.3390/ma19214702

**Published:** 2026-11-05

**Authors:** Kaiyuan Yang, Longqi Liu, Zhenhao Fan, Xinting Lu, Changqing Jia, Xingcan Mu, Bin Liu, Jianbin Zheng

**Affiliations:** 1 Transportation Institute, Inner Mongolia University, Hohhot 010020, China; yang15225536396@yeah.net (K.Y.);; 2 Key Laboratory of Green Resource Utilization of Civil Engineering Waste, Universities of Inner Mongolia Autonomous Region, Hohhot 010020, China; 3 Inner Mongolia Engineering Research Center of Testing and Strengthening for Bridges, Inner Mongolia University, Hohhot 010020, China; 4 State Grid (Jinhua) Integrated Energy Service Company, No. 98 Jiefang East Road, Jinhua 321000, China; 5 General Office, Baotou Open University, Baotou 014010, China

In the original publication [1], there was an error regarding the affiliation of Mr. Zhenhao Fan; the affiliation has been updated from “China Electronics Technology Group Corporation No. 29 Research Institute to Transportation Institute” to “Transportation Institute, Inner Mongolia University, Hohhot 010020, China”.

Correspondingly, the Section “Conflicts of Interest” has been updated to following version:

Author Xinting Lu was employed by State Grid (Jinhua) Integrated Energy Service Company. The remaining authors declare that the research was conducted in the absence of any commercial or financial relationships that could be construed as a potential conflict of interest.

The authors state that the scientific conclusions are unaffected. This correction was approved by the Academic Editor. The original publication has also been updated.
